# Whole-Cell Biocatalysis for the Production of Structurally Diverse Methoxydihydrochalcones: Broad Activity of the *Yarrowia* Clade

**DOI:** 10.3390/molecules31061049

**Published:** 2026-03-22

**Authors:** Paweł Chlipała, Marcelina Mazur, Anna Kancelista, Zbigniew Lazar, Tomasz Janeczko

**Affiliations:** 1 Department of Food Chemistry and Biocatalysis, Wrocław University of Environmental and Life Sciences, 50-375 Wrocław, Poland; marcelina.mazur@upwr.edu.pl; 2 Department of Biotechnology and Food Microbiology, Wrocław University of Environmental and Life Sciences, 51-630 Wrocław, Poland; anna.kancelista@upwr.edu.pl (A.K.); zbigniew.lazar@upwr.edu.pl (Z.L.)

**Keywords:** *Yarrowia* clade, whole-cell biocatalysis, *Yarrowia lipolytica*, methoxychalcone, dihydrochalcone, ene-reductase, substrate specificity, green chemistry

## Abstract

Whole-cell biocatalysis presents a sustainable and efficient approach for the selective reduction in α,β-unsaturated bonds in flavonoid derivatives. This study investigates the capability of yeast strains from the *Yarrowia* clade to catalyze the chemoselective reduction of 4′-methoxychalcone (**1a**) to its dihydro derivative. All tested strains exhibited similarly high hydrogenation activity, indicating a broadly conserved enoate reductase function within the clade. Among them, *Yarrowia lipolytica* KCh 71, previously reported and well characterized in the literature, was selected for preparative-scale transformation of a diverse series of synthetic methoxychalcones bearing additional methoxy groups in positions C-2, C-3, C-4, C-5, and C-6 of ring B. All derivatives were effectively converted into the corresponding dihydrochalcones, with yields ranging from 62% to 92%. Among the tested derivatives, the 2′,4′,6′-trimethoxy chalcone (**7a**) did not undergo biotransformation under our conditions, whereas mono- and di-methoxy derivatives (**2a**–**6a**) were efficiently reduced. These results confirm the broad substrate tolerance, high efficiency, and potential scalability of *Y. lipolytica* KCh 71, supporting its potential as a whole-cell biocatalyst for the sustainable synthesis of bioactive dihydrochalcones. The consistently high hydrogenation activity observed across 21 tested strains suggests the involvement of evolutionarily conserved enoate reductases. Bioinformatic analysis supports that the *Yarrowia* clade possesses a robust complement of Old Yellow Enzymes (OYE), providing a reliable enzymatic basis for the observed chemoselective reductions. All *Yarrowia* tested strains showed the same general transformation type, although the extent and rate of conversion differed among strains, and *Y. lipolytica* KCh 71 was one of the most tolerant. The broad reduction in α,β-unsaturated chalcones is consistent with the action of flavoenzymatic ene-reductases, particularly Old Yellow Enzyme (OYE)–like reductases. Bioinformatic analysis of *Yarrowia* genomes reveals putative OYE homologs, supporting this mechanistic interpretation, although the specific enzymes were not identified in this study.

## 1. Introduction

Whole-cell biocatalysis has emerged as a pivotal tool in modern biotechnology, offering a sustainable and efficient method for producing a wide range of valuable chemicals [1]. Unlike isolated enzymes, whole cells serve as self-sufficient biocatalytic systems, capable of performing complex, multi-step reactions within a stable environment. These systems offer significant advantages, such as the ability to regenerate cofactors and maintain enzyme activity over longer periods [2]. However, the success of whole-cell biocatalysis largely depends on selecting the most suitable biocatalyst, one that ensures optimal substrate conversion, high catalytic efficiency, and robust performance under industrial conditions [3]. The choice of biocatalyst is, therefore, a critical factor in maximizing product yield and achieving cost-effective production processes.

One promising group of microorganisms in this context is the *Yarrowia* clade, particularly *Yarrowia lipolytica*, which has gained increasing attention for its versatility in industrial applications [4]. *Yarrowia* species are capable of metabolizing a broad spectrum of substrates, including complex organic compounds such as lipids [5], hydrocarbons [6], and other hydrophobic substances [7]. This metabolic flexibility, coupled with their ability to produce valuable metabolites like polyols and organic acids [8] or enzymes like lipases [9], makes them attractive candidates for various biotechnological applications. In industries such as biofuel production [10], pharmaceuticals [11], and nutraceuticals, *Yarrowia* species have been utilized to improve efficiency and sustainability. Moreover, *Yarrowia lipolytica*–based processes are regarded as safe for industrial and food applications. Regulatory agencies, including the U.S. Food and Drug Administration (FDA), have granted GRAS (“Generally Recognized as Safe”) status to several commercial-scale processes involving this species [12,13].

In particular, the biocatalytic production of bioactive compounds like 4′-methoxychalcones and 4′-methoxydihydrochalcones holds great promise. These compounds are natural derivatives of the flavonoid biosynthesis pathway, predominantly found in plants [14]. 4′-methoxychalcones have been widely studied for their potent biological activities, including anti-inflammatory [15,16], antioxidant [16,17] and antimicrobial effects [18,19]. Meanwhile, 4′-methoxydihydrochalcones, their hydrogenated counterparts, exhibit similar bioactivities and are recognized for their potential in managing metabolic disorders. Both classes of compounds offer significant pro-health benefits, such as cardioprotective [20], neuroprotective [21], and anti-diabetic [22] properties, positioning them as valuable candidates for pharmaceutical and nutraceutical development.

Methoxy substituents act as electron-donating groups that delocalize electron density into the conjugated α,β-unsaturated carbonyl system. Spectroscopic and quantum-chemical studies show that such resonance donation lowers the C=C and C=O stretching frequencies and bathochromically shifts UV–Vis absorbance bands, indicating partial weakening of the double bond [23]. This modulation of bond order affects hydride transfer by flavoenzymatic ene-reductases (Old Yellow Enzyme family): electron-withdrawing groups generally increase reactivity in hetero-Michael additions, whereas strongly donating or sterically hindering groups can slow the reaction [24]. Nonetheless, moderate electron donation combined with increased hydrophobicity can improve substrate binding in the OYE active site, which may explain why methoxylated chalcones are reduced efficiently in our whole-cell system.

Given the increasing demand for these bioactive compounds, developing efficient and scalable biocatalytic processes for their production is of great importance. This study aims to explore the potential of whole-cell biocatalysis, particularly using *Yarrowia* clade species, in optimizing the conversion of 4′-methoxychalcones to 4′-methoxydihydrochalcones. By selecting the most efficient biocatalyst, this work seeks to enhance substrate conversion efficiency, improve yields, and ultimately contribute to the sustainable production of these valuable compounds. In this study, we first compared the catalytic capabilities of closely related *Yarrowia* clade species in the hydrogenation of 4′-methoxychalcone. The screening revealed generally similar biotransformation efficiencies, with *Y. lipolytica* KCh 71 showing one of the highest conversion under the applied conditions. This strain was selected for preparative-scale transformations of additional methoxychalcone derivatives due to its superior catalytic performance, robustness in whole-cell biotransformations, and its well-documented use in chalcone reduction in previous studies. In particular, we have previously employed *Y. lipolytica* KCh 71 for the hydrogenation of other chalcone derivatives. Using the same strain here allows direct comparison with those earlier results and provides continuity across our research program.

Given the increasing demand for these bioactive compounds, developing efficient and scalable biocatalytic processes is essential. Whole-cell biocatalysis using non-conventional yeasts of the *Yarrowia* clade offers a promising strategy because these organisms efficiently reduce the C=C bond in various substituted chalcones; for instance, *Y. lipolytica* has been reported to transform methoxy-, bromo-, furyl- and thienyl-chalcones to the corresponding dihydrochalcones [25,26,27], often at higher rates than other yeasts [25,26,28]. *Y. lipolytica* is also recognized as an established industrial microorganism, authorized as a novel food in the EU, and can achieve conversions above 90% within a few hours for hydrogenation of chalcone substrates. Strain KCh 71, in particular, has been described as a practical and robust biocatalyst delivering fast, high-selectivity reductions across diverse substitution patterns [27,29]. Based on these literature reports highlighting its broad substrate specificity, operational robustness and regulatory acceptance, we selected *Y. lipolytica* KCh 71 as the biocatalyst for preparative-scale work in this study.

## 2. Results and Discussion

This study aims to select the most suitable and the most efficient biocatalyst among species of the *Yarrowia* clade (Table 1). The primary goal was to find a strain that most effectively transforms the tested 4′-methoxychalcone (**1a**) into the corresponding dihydrochalcone (**1c**).

**Figure 1:**
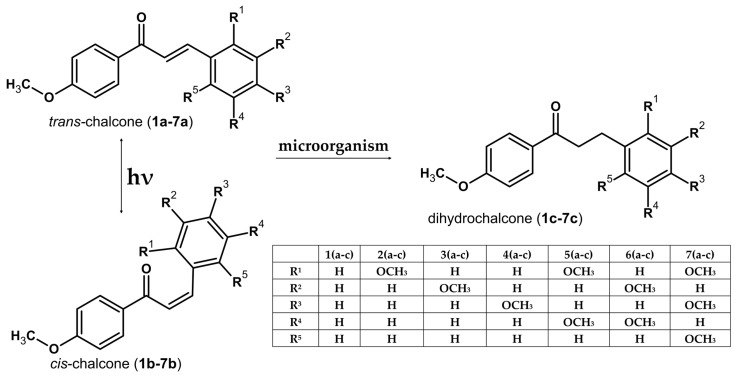
Light-induced *trans* → *cis* isomerisation of chalcones (**1a**–**7a**) solutions to corresponding *cis*-chalcones (**1b**–**7b**), and their subsequent transformations to 4′-methoxydihydrochalcones (**1c**–**7c**) using *Y. lipolytica* KCh 71. The transformation is chemoselective and involves reduction in the α,β-unsaturated C=C bond of the chalcone linker. Compound labels “a” denote chalcone substrates, whereas labels “c” denote the hydrogenated products (dihydrochalcones). R^1^–R^5^ indicate the methoxy substitution pattern on ring B (positions C-2, C-3, C-4 and C-5, respectively).

Our previous works proved that either *Y. lipolytica* or other yeast species like *Saccharomyces cerevisiae* or *Rhodotorula rubra* are capable of reducing the double C=C bond in prop-2-en-1-on linker in differently substituted chalcones [25,29]. These works demonstrated that either hydroxychalcones and bromochalcones or furylchalcones and thienylchalcones had been effectively transformed into their corresponding dihydrochalcones by *Y. lipolytica*. The 2‘-hydroxychalcones tested were generally transformed similarly or faster than the similarly substituted 4′-hydroxychalcones and both groups of compounds mentioned were more efficiently transformed than chalcones containing a bromine atom attached to the B ring of chalcone [29,30]. The double C=C bond in furylchalcone and thienylchalcone was reduced with the use of *Y. lipolytica* on higher rates [26] than in bromochalcones [25] and similar to hydroxychalcones [28,29]. The formation of *cis*-chalcone (**2**) was observed as in the case of 4′-hydroxychalcones [29]. All tested *Yarrowia* clade species catalyzed the same general transformation of 4′-methoxychalcone, namely chemoselective reduction in the α,β-unsaturated C=C bond; however, the extent and rate of conversion differed among strains (Figure 1). This suggests that the enoate reductase activity responsible for C=C bond saturation is broadly conserved in this clade indicating that it is likely a conserved metabolic trait across the clade. Comparable findings were previously described for isolated OYE-like enzymes from *Y. lipolytica*, which effectively catalyze reductions in α,β-unsaturated ketones under mild and cofactor-free conditions [25,26,28,29,30]. These results collectively confirm that the *Yarrowia* whole-cell system maintains an efficient internal redox environment enabling such transformations. Given the similar performance across strains, *Y. lipolytica* KCh 71 was chosen for preparative-scale transformations of a series of methoxychalcone derivatives bearing one to three additional methoxy substituents in various positions on ring B. This strain was selected due to its proven robustness, catalytic efficiency, and extensive documentation in the literature [27,29,30].

The biotransformation of 4′-methoxychalcone (**1a**) to 4′-methoxydihydrochalcone (**1c**) by *Yarrowia* clade species, as detailed in Table 2, showcases the efficiency of whole-cell biocatalysis in reducing α,β-unsaturated bonds, achieving high conversion rates (e.g., 94.6% for *Y. lipolytica* ClY29-26-3 and 84.3% for *Candida galli* CBS9722 at 24 h). This process, driven by enoate reductases (likely OYE homologues), targets the C=C bond in the prop-2-en-1-one linker of chalcones, yielding bioactive flavonoid derivatives with pharmaceutical and nutraceutical potential. The *cis*-chalcone (**1b**) is neither an intermediate nor a byproduct but an isomer of (**1a**) [29] formed through a non-yeast-mediated process, possibly during substrate preparation or chemical handling prior to biocatalysis.

Relative composition (%) of the reaction mixture during the reduction in *trans*-4′-methoxychalcone (**1a**) by strains of the *Yarrowia* clade (UPLC/UHPLC-DAD). Biotransformations were performed in growing cultures in Sabouraud medium (3% glucose, 1% peptone) in 300 mL Erlenmeyer flasks containing 100 mL medium, shaken at 131 rpm and 25 °C. Substrate was added as a DMSO stock solution (10 mg in 1 mL DMSO; final DMSO ~1% *v*/*v*). Samples were collected 1 h, 12 h and 24 h after substrate addition. Values are mean ± SD (n = 3). **1a**—*trans*-4′-methoxychalcone; **1b**—*cis*-4′-methoxychalcone; **1c**—4′-methoxy dihydrochalcone.

A series of methoxylated chalcones (**2a**–**7a**) were synthesized via Claisen–Schmidt condensation and subjected to biotransformation with *Y. lipolytica* KCh 71 under identical whole-cell conditions used for compound 1. The substrates included derivatives with methoxy groups at C-2 (**2a**), C-3 (**3a**), C-4 (**4a**), C-2 and C-5 (**5a**), C-3 and C-5 (**6a**), and C-2, C-4, and C-6 (**7a**) positions of ring B. Chalcones **2a**–**6a** were effectively reduced to the corresponding dihydrochalcones, reaching near-complete conversion within the first hour. Although conversions were high, isolated yields (62–92%) were lower due to recovery losses during extraction/evaporation and preparative TLC. In addition, only the highest-purity TLC fractions were pooled for structural characterization; fractions containing co-extractives and endogenous yeast metabolites were intentionally discarded, which inevitably reduced the overall mass recovery. No conversion was observed for the 2′,4′,6′-trimethoxy derivative (**7a**). These results are consistent with the electron-donating nature of methoxy groups in *ortho* and *para* positions, enhancing substrate activation for enolate reductase catalysis. Our bioinformatic analysis suggests that this activity is likely mediated by a conserved family of Old Yellow Enzymes (OYEs).

The investigation was initiated using the crystal structure of Old Yellow Enzyme 3 (OYE3) from *Saccharomyces cerevisiae* (PDB: 5V4V), which corresponds to the reference protein NP_015154.1. Given that the *Yarrowia* clade lacks extensive protein annotations, we employed a multi-step homology-based search. An initial BLASTp (BLASTP 2.17.0+) analysis against the non-redundant database identified the protein KAG5370926.1 (from *Yarrowia* sp. E02) as a high-confidence ortholog, providing a sequence bridge to the *Yarrowia* genus.

To further explore the genomic diversity of this clade, we performed a tBLASTn search against the Whole Genome Shotgun (WGS) database. This allowed for the identification of OYE-encoding loci directly from unannotated genomic assemblies. Specifically, we retrieved full-length sequences from *Yarrowia osloensis* CBS 10146 (contig YAOS0S02, ULGU01000002.1) and *Yarrowia bubula* CBS 12934 (contig YABU0S02, ULGX01000002.1).

Comparative analysis between the *Yarrowia* sequences and the OYE3 template revealed significant evolutionary divergence. The identified sequence for *Y. osloensis* shared 68.4% identity, while the sequence for *Y. bubula* showed 50.7% identity. Despite this sequence-level divergence, the query coverage remained at 98–100% for all retrieved orthologs. This nearly total coverage confirms the strict maintenance of the TIM-barrel architecture and the essential active-site geometry across the tested clade [31].

This observation is consistent with structural work on OYE/PETNR family enzymes showing that the substrate-binding pocket and entrance channel can act as steric ‘gates’, restricting access and productive orientation of bulky α,β-unsaturated substrates. Structure-guided mutagenesis of PETNR and related ene-reductases has demonstrated that substitutions at pocket/entrance residues can modulate substrate accommodation and catalytic performance. Moreover, recent engineering studies on OYE homologues explicitly targeted steric hindrance at the entrance/pocket region to enable conversion of more sterically demanding substrates. Therefore, the three methoxy substituents in 7a likely prevent the reactive C=C bond from adopting the geometry required for hydride transfer in an OYE/PETNR-like active site [32,33,34,35]. Similar steric effects were reported to suppress OYE-mediated reductions in highly substituted chalcones and enones [34,36,37,38,39,40,41,42,43,44,45,46,47].

The *Yarrowia*-mediated reduction of 4′-methoxychalcone (**1a**) to (**1c**) demonstrates robust performance, with strains like *Y. lipolytica* ClY29-26-3 achieving 94.6% conversion to (**1c**) and only 0.8% unreacted (**1a**) at 24 h. In contrast, *Y. yakushimensis* CBS10253 yields 39.3% (**1c**), with 50.4% of the *cis*-chalcone isomer (**1b**) and 10.3% unreacted (**1a**), suggesting that (**1b**) is present in the substrate mixture and less effectively reduced by this strain. Previous studies show *Y. lipolytica* reduces other chalcones, such as 2′-hydroxychalcones (up to 95%) [28], furylchalcones (85–90%) and thienylchalcones (80–90%) [26], outperforming bromochalcones (50–70%) [25] due to electron-donating groups enhancing substrate affinity. The 4′-methoxyl group in (**1a**) likely stabilizes the transition state, facilitating reduction, as seen in OYE reductions in electron-rich substrates [34].

Comparatively, *Saccharomyces cerevisiae* and *Rhodotorula rubra* achieve lower conversions (50–80%) for chalcones [29], often due to alcohol dehydrogenase (ADH) activity forming alcohols [36]. *Y. lipolytica* KCh 71 reduces 2′-hydroxychalcones to its dihydrochalcone with comparable conversion after 7 days [28], as *Y. lipolytica* ClY29-26-3 94.6% for (**1c**) after just 24 h. *Yarrowia* efficient membrane transport and lipid metabolism enhance hydrophobic chalcone uptake [37], providing an advantage over other yeasts.

Enals, such as cinnamaldehyde and citral, are reduced by OYE1-3 (old yellow enzyme) from *S. cerevisiae* or *Saccharomyces pastorianus* to (S)-α-methyldihydrocinnamaldehyde derivatives (e.g., Lilial™) with 100% conversion and >95% enantiomeric ratio (er) in biphasic systems (20% t-BuOMe) [38]. Similarly, (E/Z)-citral is converted to (S)- or (R)-citronellal with 100% and 69% conversion, respectively and >95% ee using isolated OYEs [39]. These high conversions align with *Yarrowia*’s performance for (**1a**), but whole-cell *Yarrowia* systems eliminate the need for cofactor supplementation, reducing costs [2]. Unlike enal reductions, where ADHs in *S. cerevisiae* produce saturated alcohols (20–30%) [36], *Yarrowia*’s chalcone reduction avoids alcohol formation, with (**1b**) present as an isomer in the substrate mixture rather than a yeast-generated product [29].

OYE1 and pentaerythritol tetranitrate reductase (PETNR) from *Enterobacter cloacae* reduce (R)-carvone to dihydrocarvone with >90% conversion and 98% ee [40], comparable to *Yarrowia*’s 89.6% for (**1c**). Strains with high (**1b**) levels (e.g., *Y. yakushimensis*, 71.50% at 24 h) may have lack of sufficient cofactor pools, a limitation also noted in OYE reductions in nitriles (50–80% conversion) due to weak substrate activation [41]. PETNR also reduced ketoisophorone to (2*R*)-levodione with 99% of yield and 49% of ee [39].

*Yarrowia lipolytica’s* high conversion rates (up to 94.6% for (**1c**)) and stereoselectivity make it a superior platform for producing dihydrochalcones, surpassing *S. cerevisiae* and rivaling isolated OYE systems for enals and enones. Its GRAS (Generally Recognized As Safe) [42] status and metabolic versatility [43] support pharmaceutical and nutraceutical applications, leveraging dihydrochalcone’s anti-inflammatory [44] and anti-diabetic [45] properties. While enal and nitroalkene reductions achieve high ee, they often require purified enzymes or cascades, increasing costs. *Yarrowia lipolytica* whole-cell approach simplifies processes, but optimizing reduction in both (**1a**) and its isomer (**1b**) is critical for industrial scalability, particularly in strains less efficient with (**1b**).

Future studies should characterize *Yarrowia*’s OYE homologues to understand substrate specificity for (**1a**) and (**1b**), enabling targeted engineering to enhance reduction in both isomers but chalcone **7a** did not undergo biotransformation. Kinetic studies could identify rate-limiting steps, and computational modeling of chalcone-enzyme interactions could optimize substrate design. Exploring *Yarrowia* clade for other α,β-unsaturated substrates (e.g., maleimides, nitriles) could broaden its biotechnological applications, building on its success with chalcones.

*Yarrowia* clade species excel in reducing the α,β-unsaturated bond of 4′-methoxychalcone, achieving conversions up to 94.6% that rival OYE-mediated reductions in enals, enones and nitroalkenes (70–100%) [46,47]. The *cis*-chalcone (**1b**), as an isomer of (**1a**) highlights the importance of substrate preparation in biotransformation outcomes. By optimizing reduction in both isomers through strain engineering and bioprocess improvements, *Yarrowia* can advance sustainable production of bioactive compounds, bridging laboratory and industrial biocatalysis. Taken together, these findings highlight the remarkable catalytic potential of *Yarrowia* clade species as sustainable whole-cell biocatalysts for chalcone hydrogenation. Their high efficiency, stability, and broad substrate scope emphasize their technological relevance for future applications in the biotransformation of flavonoids and other α,β-unsaturated compounds.

## 3. Materials and Methods

### 3.1. Substrates

**Figure 2:**
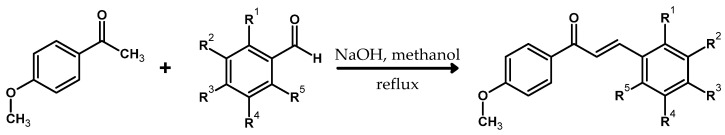
Synthesis scheme of 4′-methoxychalcones via Claisen-Schmidt condensation. R^1–5^ = -OCH_3_.

Chalcones for biotransformation were prepared through Claisen–Schmidt condensation using 4-methoxyacetophenone and benzaldehyde with methoxyl group(s) (Figure 2) at appropriate positions, sourced from Sigma-Aldrich (St. Louis, MO, USA).

To synthesize the chalcones, 50 mmoles each of acetophenone and benzaldehyde were dissolved in 150 mL methanol in a 500 mL round-bottom flask. Subsequently, 30 mL water and 5.0 g sodium hydroxide (NaOH) were added slowly. The mixture was refluxed for 2 h, with reaction progress tracked via thin-layer chromatography (TLC). Once complete, the mixture was poured into a 1 L beaker, neutralized with 1 M HCl, stirred thoroughly to precipitate the chalcone, and left for 24 h. The crude chalcone was isolated by vacuum filtration, then purified by recrystallization in ethanol: dissolved under reflux, cooled to room temperature, and left for 24 h to crystallize. The crystals were collected via vacuum filtration with a Büchner funnel, dried, and analyzed by NMR to confirm purity and structure. The resulting compounds (**1a**–**8a**)—including 4′-methoxychalcone and its derivatives with additional methoxy groups in various positions of ring B—were used as biotransformation substrates (see Table 3). The purity and structures of all synthesized chalcones were confirmed by ^1^H NMR, ^13^C NMR (Table 3), HMBC, HMQC, and COSY analyses. Our goal was to obtain highly purified chalcone substrates for biotransformation, rather than to optimize synthetic yields. For this reason we recrystallized the crude products to maximize purity, monitored the reactions by TLC to ensure complete conversion, and did not determine isolated yields. The purity of each chalcone was verified by comprehensive NMR analysis and by UPLC (near-quantitative purity), with the full spectra provided in the Supplementary Materials.

4′-methoxychalcone (**1a**): ^1^H NMR (400 MHz, acetone) δ 8.19–8.14 (m, 2H, H-2′ and H-6′), 7.88 (d, 1H, *J* = 15.6 Hz, H-β), 7.85–7.80 (m, 2H, H-2 and H-6), 7.76 (d, *J* = 15.6 Hz, 1H, H-α), 7.49–7.43 (m, 3H, H-3 and H-4 and H-5), 7.11–7.05 (m, 2H, H-3′ and H-5′), 3.91 (s, 3H, C-4′-OCH_3_).

2,4′-dimethoxychalcone (**2a**): ^1^H NMR (600 MHz, Acetone) δ 8.15–8.12 (m, 3H, H-β, H-2′ and H-6′), 7.86 (dd, *J* = 7.7, 1.6 Hz, 1H, H-6), 7.86 (d, *J* = 15.7 Hz, 1H, H-α), 7.41 (ddd, *J* = 8.3, 7.1, 1.6 Hz, 1H, H-4), 7.09 (d, *J* = 8.3, Hz, 1H, H-3), 7.08–7.05 (m, 2H, H-3′ and H-5′), 7.01 (s, *J* = 7.5 Hz, 1H, H-5), 3.94 (s, 3H, C-2-OCH_3_), 3.90 (s, 3H, C-4′-OCH_3_).

3,4′-dimethoxychalcone (**3a**): ^1^H NMR (600 MHz, Acetone) δ 8.18–8.14 (m, 2H, H-2′ and H-6′), 7.88 (d, *J* = 15.6 Hz, 1H, H-α), 7.73 (d, *J* = 15.6 Hz, 1H, H-β), 7.41–7.39 (m, 1H, H-2), 7.39–7.37 (m, 1H, H-4), 7.36 (s, *J* = 7.5 Hz, 1H, H-5), 7.09–7.05 (m, 2H, H-3′ and H-5′), 7.01 (dt, *J* = 7.1, 2.3 Hz, 1H, H-6), 3.91 (s, 3H, C-4′-OCH_3_), 3.86 (s, 3H, C-3-OCH_3_).

4,4′-dimethoxychalcone (**4a**): ^1^H NMR (600 MHz, Acetone) δ 8.17–8.11 (m, 2H, H-2′ and H-6′), 7.79–7.75 (m, 2H, H-2 and H-6), 7.75 (d, *J* = 15.6 Hz, 1H, 1H, H-α), 7.88 (d, *J* = 15.6 Hz, 1H, H-β), 7.08–7.04 (d, m, 2H, H-3′ and H-5′), 7.02–6.99 (d, m, H-3 and H-5), 3.90 (s, 3H, C-4′-OCH_3_), 3.86 (s, 3H, C-4-OCH_3_).

2,5,4′-trimethoxychalcone (**5a**): ^1^H NMR (600 MHz, Acetone) δ 8.13–8.09 (m, 2H, H-2′ and H-6′), 8.07 (d, *J* = 15.7 Hz, 1H, H-β), 7.80 (d, *J* = 8.6 Hz, 1H, H-3), 7.74 (d, *J* = 15.7 Hz, 1H, H-α), 7.07–7.03 (m, 2H, H-3′ and H-5′), 6.64 (d, *J* = 2.4 Hz, 1H, H-6), 6.61 (dd, *J* = 8.6, 2.4 Hz, 1H, H-4), 3.95 (s, 3H, C-2-OCH_3_), 3.89 (s, 3H, C-4′-OCH_3_), 3.87 (s, 3H, C-5-OCH_3_).

3,5,4′-trimethoxychalcone (**6a**): ^1^H NMR (600 MHz, Acetone) δ 8.18–8.11 (m, 2H, H-2′ and H-6′), 7.86 (d, *J* = 15.5 Hz, 1H, H-α), 7.68 (d, *J* = 15.6 Hz, 1H, H-β), 7.09–7.05 (m, 2H, H-3′ and H-5′), 6.99 (d, *J* = 2.1 Hz, 2H, H-2 and H-6), 6.57 (t, *J* = 2.2 Hz, 1H, H-4), 3.91 (s, 3H, C-4′-OCH_3_), 3.85 (s, 6H, C-3′ and C-5′-OCH_3_).

2,4,6,4′-tetramethoxychalcone (**7a**): ^1^H NMR (600 MHz, Acetone) δ 8.23 (d, *J* = 15.7 Hz, 1H, H-β), 8.05–8.03 (m, 2H, H-2′ and H-6′), 7.98 (d, *J* = 15.7 Hz, 1H, H-α), 7.06–7.02 (m, 2H, H-3′ and H-5′), 6.32 (s, 2H H-3 and H-5), 3.96 (s, 6H, C-2 and C-6-OCH_3_), 3.89 (s, 3H, C-4′-OCH_3_), 3.88 (s, 3H, C-4-OCH_3_).

### 3.2. Microorganisms

The experimental material comprised 21 yeast strains representing the genera *Yarrowia* and *Candida*. The specific strains utilized in this study were: *Yarrowia lipolytica* (W-29, 24lIV, 24lI, H222, ClY29-26-3, A101, KCh 71), *Y. phangngaensis* (CBS10407), *Y. parophonii* (CBS12427), *Y. bubula* (CBS12934), *Y. keelungensis* (CBS11062), *Y. brassicae* (CBS15225), *Y. deformans* (CBS2071), *Y. divulgata* (CBS11013), *Y. yakushimensis* (CBS10253), *Y. porcina* (CBS12932), *Candida osloensis* (CBS10146), *C. hispaniensis* (CBS9996), *C. alimentaria* (CBS10151), *C. galli* (CBS9722), and *C. hollandica* (CBS4855). With the exception of strain KCh 71, which was obtained from the Department of Food Chemistry and Biocatalysis, all strains were provided by the Department of Biotechnology and Food Microbiology at the Wrocław University of Environmental and Life Sciences (Wrocław, Poland). The storage conditions and characteristics for these isolates have been previously documented [20,25,41]. The strains were stored in a 25% glycerol solution at −80 °C and periodically inoculated onto solid YPG medium (Yeast extract-Peptone-Glycerol) medium and stored at 4 °C. Using a sterilized inoculation loop, the yeast cells were transferred from the YPG agar slants into 300 mL conical flasks containing 100 mL of sterile liquid medium. The cultures were then incubated at 25 °C for 48 h on a rotary shaker (131 rpm) to prepare pre-incubation cultures.

### 3.3. Screening

A total of 0.5 mL of inoculum of the tested yeast strains from the pre-incubation culture was transferred to 300 mL Erlenmeyer flasks for analytical-scale biotransformation. Each flask contained 100 mL of Sabouraud culture medium (3% glucose, 1% peptone) and was incubated for two days at 25 °C on a rotary shaker set to 131 rpm. After this time, 10 mg of the substrate was dissolved in 1 mL of DMSO (dimethyl sulfoxide) and added to the biocatalyst culture. Samples were collected after 1, 3, 6, 12 and 24 h. Portions of 10 mL of the transformation mixture were taken out and extracted with 10 mL of ethyl acetate. The extracts were dried over MgSO_4_, concentrated in vacuo and analyzed using Ultra Performance Liquid Chromatography (ULPC) and thin-layer chromatography (TLC). All experiments were conducted in triplicate as three independent biological replicates to confirm data reproducibility. Substrate stock solutions (in DMSO) and biotransformation samples were handled under standard laboratory lighting conditions and were not protected from light (the experiments were performed before we introduced light-exclusion precautions for chalcone biotransformations). Under these conditions, the *cis*/*trans* isomer (1a/1b) could be detected by UPLC as part of the reaction mixture composition. Because light exposure was not controlled, we cannot unambiguously assign whether *trans* → *cis* isomerisation occurred during the biotransformation or during subsequent sample handling.

### 3.4. Preparative Scale

Preparative biotransformations with methoxychalcones **2a**–**7a** were performed only with *Y. lipolytica* KCh 71, were carried out in 2 L Erlenmeyer flasks containing 500 mL of culture medium (3% glucose, 1% peptone). The yeast strain *Y. lipolytica* KCh 71 was cultured for three days at 25 °C on a rotary shaker set to 131 rpm. Then, 100 mg of substrate dissolved in 2 mL DMSO was added. After seven days, the product was extracted three times with 300 mL ethyl acetate, dried with anhydrous magnesium sulfate, and evaporated under reduced pressure. The biotransformation products were purified by preparative TLC and characterized using TLC, UPLC, and NMR (Table 4). Only fractions corresponding to the pure target product (as assessed by TLC and UPLC-DAD) were combined and used for yield calculation and NMR characterization.

4′-methoxydihydrochalcone (**1c**): ^1^H NMR (600 MHz, Acetone) δ 8.02–7.99 (m, 2H, H-2′ and H-6′), 7.30 (dd, *J* = 8.1, 1.4 Hz, 2H, H-2 and H-6), 7.28–7.26 (m, 2H, H-3 and H-5), 7.19–7.15 (m, 1H, H-4), 7.03–6.99 (m, 2H, H-3′ and H-5′), 3.88 (s, 3H, C-4′-OCH_3_), 3.32–3.27 (m, 2H, H-α), 3.03–2.98 (m, 2H, H-β).

2,4′-dimethoxydihydrochalcone (**2c**): ^1^H NMR (600 MHz, Acetone) δ 8.01–7.97 (m, 2H, H-2′ and H-6′), 7.21 (dd, *J* = 7.4, 1.6 Hz, 1H, H-6), 7.18 (td, *J* = 7.4, 1.6 Hz, 1H, H-4), 7.04–7.00 (m, 2H, H-3′ and H-5′), 6.95 (d, *J* = 8.0 Hz, 1H, H-3), 6.86 (td, *J* = 7.4, 1.0 Hz, 1H, H-5), 3.88 (s, 3H, C-4′-OCH_3_), 3.84 (s, 3H, C-2-OCH_3_), 3.23–3.17 (m, 2H, H-α), 2.99–2.94 (m, 2H, H-β).

3,4′-dimethoxydihydrochalcone (**3c**): ^1^H NMR (600 MHz, Acetone) δ 8.02–7.98 (m, 2H, H-3′ and H-5′), 7.18 (t, *J* = 7.9 Hz, 1H, H-5), 7.03–6.99 (m, 2H, H-2′ and H-6′), 6.90–6.87 (m, 1H, H-2), 6.87–6.85 (m, 1H, H-4), 6.74 (ddd, *J* = 8.2, 2.5, 0.5 Hz, 1H, H-6), 3.87 (s, 3H, C-4′-OCH_3_), 3.76 (s, 3H, C-3-OCH_3_), 3.31–3.26 (m, 2H, H-α), 2.99–2.95 (m, 2H, H-β).

4,4′-dimethoxydihydrochalcone (4c): ^1^H NMR (600 MHz, Acetone) δ 8.02–7.95 (m, 2H, H-2′ and H-6′), 7.22–7.17 (d, m, 2H, H-2 and H-6), 7.02–6.96 (m, 2H, H-3′ and H-5′), 6.89–6.77 (m, 2H, H-3 and H-5), 3.87 (s, 3H, C-4′-OCH_3_), 3.75 (s, 3H, C-4-OCH_3_), 3.27–3.21 (m, 2H, H-α), 2.95–2.91 (t, m, 2H, H-β).

2,5,4′-trimethoxydihydrochalcone (**5c**): ^1^H NMR (600 MHz, Acetone) δ 8.02–7.94 (m, 2H, H-2′ and H-6′), 7.09 (d, *J* = 8.2 Hz, 1H, H-3), 7.03–6.97 (m, 2H, H-3′ and H-5′), 6.52 (d, *J* = 2.4 Hz, 1H, H-6), 6.43 (dd, *J* = 8.2, 2.4 Hz, 1H, H-4), 3.87 (s, 3H, C-4′-OCH_3_), 3.83 (s, 3H, C-2-OCH_3_), 3.76 (s, 3H, C-5-OCH_3_), 3.17–3.13 (m, 2H, H-α), 2.91–2.86 (m, 2H, H-β).

3,5,4′-trimethoxydihydrochalcone (**6c**): ^1^H NMR (600 MHz, Acetone) δ 8.03–7.98 (m, 2H, H-2′ and H-6′), 7.05–6.99 (m, 2H, H-3′ and H-5′), 6.47 (d, *J* = 2.2 Hz, 2H H-2 and H-6), 6.31 (t, *J* = 2.3 Hz, 1H, H-4), 3.88 (s, 3H, C-4′-OCH_3_), 3.75 (s, 6H, C-3-OCH_3_ and C-5-OCH_3_), 3.30–3.26 (m, 2H, H-α), 2.95–2.91 (m, 2H, H-β).

### 3.5. TLC and NMR Analysis

Biotransformation’s course was monitored using TLC plates (SiO_2_, DC Alufolien Kieselgel 60 F254 (0.2 mm thick), Merck, Darmstadt, Germany). The products were separated using preparative TLC plates (Silica Gel GF, 20 × 20 cm, 500 µm, Analtech, Newark, DE, USA) and developed in a mixture of cyclohexane and ethyl acetate (9:0.5, *v*/*v*) as the eluent. The product was observed (without additional visualization) under the UV lamp at a wavelength of 254 nm. The NMR analysis was performed using a DRX 600 MHz Bruker spectrometer (Bruker, Billerica, MA, USA). The prepared samples were dissolved in deuterated DMSO and acetone-d_6_. The performed analyses include ^1^H-NMR, ^13^C-NMR, HMBC (two-dimensional analysis), HMQC (heteronuclear correlation), and COSY (correlation spectroscopy) (all NMR spectra are in Supplementary Materials).

### 3.6. UPLC

The biotransformation was analyzed using a UPLC-DAD method on a Dionex Ulti-mate 3000 UHPLC+ system (Thermo Fisher Scientific, Waltham, MA, USA). The system was equipped with a DGP-3600A dual pump liquid control compartment, a TCC-3200 thermostatted column module, a WPS-3000 autosampler, a diode array detector (DAD), and an analytical Cosmosil Cholester Waters column (5 μm, 4.6 × 250 mm, Nacalai Tesque Inc., Kyoto, Japan) maintained at 28 °C. Separation was performed using two mobile phases: (A) 0.1% formic acid in water and (B) 0.1% formic acid in acetonitrile, with the following gradient program: 0–13 min, 25–95% B; 13–15 min, 95% B; 15–17 min, 95–25% B; and 17–20 min, 25% B, at a flow rate of 0.7 mL/min. System control and data acquisi-tion were carried out using Chromeleon 6.80 software (Dionex, Sunnyvale, CA, USA). Substrates and products were identified by comparison with authentic standards at a de-tection wavelength of 280 nm, based on retention times and UV spectra.

### 3.7. Bioinformatics Analysis

The investigation was initiated using the structural template of OYE3 from *Saccharomyces cerevisiae* S288C (PDB: 5V4V), corresponding to the reference protein sequence NP_015154.1. Due to the limited characterization of the *Yarrowia* genus, a multi-step homology search strategy was employed.

Initial protein-protein BLAST (BLASTp) searches were conducted against the National Center for Biotechnology Information (NCBI) non-redundant (nr) protein database, which identified the protein KAG5370926.1 from *Yarrowia* sp. E02 as a high-confidence ortholog. This sequence served as a primary anchor for further genomic mining. Subsequently, to identify OYE-encoding loci in unannotated genomic data, tBLASTn (protein-to-translated nucleotide) searches were performed against the Whole Genome Shotgun (WGS) database. The search space was taxonomically restricted to the *Yarrowia* clade (taxid:4951).

High-stringency criteria were applied to ensure functional orthology, focusing on a Query Coverage (QC) threshold of 98–100% to guarantee the integrity of the protein fold. This in silico analysis supports the presence of putative OYE-like ene-reductases across the *Yarrowia* clade; however, it does not constitute experimental identification of the enzyme(s) responsible for the observed whole-cell activity [12].

## 4. Conclusions

This study demonstrates that ene-reductase activity is broadly conserved across *Yarrowia* clade species, enabling fast and selective saturation of α,β-unsaturated double bonds in methoxychalcones. Across 21 tested strains, conversions of 4′-methoxychalcone (**1a**) were consistently high within hours (Table 2), and *Y. lipolytica* KCh 71 proved to be a particularly efficient biocatalyst for structurally diverse derivatives. Mono- and di-methoxy substrates (**2a**–**6a**) were reduced almost quantitatively (>96% within 24 h; high isolated yields after 7 days), whereas the 2,4,6,4′-tetramethoxy chalcone (**7a**) remained unreacted, indicating a steric limitation for heavily ortho-substituted molecules.

Compared with conventional yeasts that often reduce both C=C and C=O functionalities, *Yarrowia* clade species displayed remarkable chemoselectivity, reducing only the C=C bond. The observed *cis*-isomers resulted from photoisomerization rather than enzymatic activity. The obtained reaction rates and space–time yields are comparable to those of isolated OYE/PETNR enzymes, but the process is simpler, cofactor-independent, and potentially scalable. The preparative runs were extended to 7 days primarily to secure sufficient material for NMR characterization; no dedicated operational stability experiments (e.g., cell recycling or activity decay measurements) were performed, and stability is discussed here only in the practical sense that biotransformation capability was retained over the experimental time window.

Future research should focus on identifying and characterizing individual OYE homologues responsible for this activity, exploring enzyme engineering strategies to expand substrate tolerance, and evaluating the biological activities of the resulting dihydrochalcones. Overall, *Y. lipolytica* KCh 71 represents a versatile and sustainable whole-cell platform for the biocatalytic production of bioactive dihydrochalcones.

## Figures and Tables

**Table 1:** *Yarrowia* clade species used in this study.

Number	Strain	Abbreviation
1	*Yarrowia lipolytica* W-29 (control)	YALI
2	*Candida osloensis* CBS10146	YAOS
3	*Yarrowia phangngaensis* CBS10407	YAPH
4	*Yarrowia parophonii* CBS12427	YAPA
5	*Candida hispaniensis* CBS9996	OLHI
6	*Yarrowia bubula* CBS12934	YABU
7	*Candida alimentaria* CBS10151	YAAL
8	*Yarrowia keelungensis* CBS11062	YAKE
9	*Yarrowia brassicae* CBS15225	YABR
10	*Yarrowia deformans* CBS2071	YADE
11	*Yarrowia divulgata* CBS11013	YADI
12	*Yarrowia yakushimensis* CBS10253	YAYA
13	*Yarrowia lipolytica* 24lIV	24lIV
14	*Yarrowia lipolytica* 24lI	24lI
15	*Yarrowia lipolytica* KCh 71	KCh71
16	*Yarrowia lipolytica* H222	H222
17	*Yarrowia lipolytica* ClY29-26-3	ClY29-26-3
18	*Yarrowia lipolytica* A101	A101
19	*Candida galli* CBS9722	YAGA
20	*Yarrowia porcina* CBS12932	YAPO
21	*Candida hollandica* CBS4855	YAHO

**Table 2:** Content (%) of reaction mixture of reduction 4′-methoxychalcone by *Yarrowia* clade species in time based on UHPLC data.

Strain	1 h			12 h			24 h		
	1a	1b	1c	1a	1b	1c	1a	1b	1c
YALI	10.9 ± 7.7	12.6 ± 9.6	76.6 ± 8.5	3.0 ± 0.8	16.2 ± 3.4	80.7 ± 4.2	2.4 ± 0.5	13.9 ± 2.8	83.7 ± 3.3
YAOS	15.2 ± 1.6	11.6 ± 3.2	73.3 ± 4.7	5.0 ± 0.4	21.9 ± 2.4	73.1 ± 2.0	3.4 ± 0.4	19.5 ± 2.4	77.1 ± 2.8
YAPH	10.0 ± 6.4	15.8 ± 4.6	74.2 ± 2.8	3.6 ± 0.4	21.5 ± 2.9	74.9 ± 3.3	2.5 ± 0.0	14.1 ± 0.4	83.4 ± 0.4
YAPA	16.0 ± 4.9	12.6 ± 2.3	71.4 ± 3.7	4.1 ± 1.1	17.1 ± 3.3	78.8 ± 4.5	2.7 ± 0.5	15.6 ± 2.8	81.7 ± 3.3
OLHI	62.1 ± 3.2	5.3 ± 1.5	32.6 ± 1.6	11.2 ± 1.1	37.0 ± 2.0	51.8 ± 1.0	4.9 ± 1.0	25.8 ± 5.1	69.2 ± 6.0
YABU	21.8 ± 2.9	14.4 ± 2.8	63.7 ± 1.3	6.6 ± 1.8	29.6 ± 0.4	63.8 ± 1.4	4.8 ± 1.3	26.5 ± 6.7	68.7 ± 8.0
YAAL	20.3 ± 1.3	17.9 ± 7.3	61.9 ± 6.4	3.9 ± 0.8	22.8 ± 3.7	73.3 ± 4.5	1.6 ± 0.6	9.1 ± 2.9	89.3 ± 3.6
YAKE	7.0 ± 6.1	11.4 ± 9.1	81.5 ± 7.8	3.2 ± 0.8	15.0 ± 4.0	81.8 ± 4.7	1.4 ± 0.5	8.7 ± 3.5	89.9 ± 4.0
YABR	10.8 ± 2.7	40.7 ± 7.6	48.5 ± 7.5	7.4 ± 0.2	30.0 ± 4.2	62.6 ± 4.5	3.8 ± 0.3	22.0 ± 1.9	74.2 ± 2.2
YADE	6.9 ± 5.5	18.9 ± 4.2	74.2 ± 9.7	3.8 ± 1.3	22.1 ± 6.5	74.1 ± 7.8	2.5 ± 1.1	13.7 ± 5.8	83.9 ± 6.9
YADI	16.7 ± 4.5	21.7 ± 7.9	61.6 ± 9.3	3.6 ± 1.2	19.8 ± 5.1	76.7 ± 6.3	2.1 ± 1.3	12.1 ± 7.8	85.8 ± 9.2
YAYA	37.7 ± 6.2	32.1 ± 2.1	30.3 ± 8.2	14.5 ± 1.2	62.1 ± 0.8	23.3 ± 1.2	10.3 ± 1.9	50.4 ± 6.7	39.3 ± 6.9
24lIV	27.9 ± 18.3	22.3 ± 6.8	49.8 ± 11.7	9.8 ± 1.8	33.1 ± 3.4	57.1 ± 4.6	3.1 ± 1.1	17.9 ± 6.2	79.0 ± 7.2
24lI	17.6 ± 7.4	29.1 ± 3.0	53.3 ± 9.0	5.2 ± 0.5	30.4 ± 2.1	64.4 ± 2.6	5.5 ± 0.6	31.8 ± 2.8	62.7 ± 3.4
KCh 71	9.0 ± 5.1	21.4 ± 10.7	69.7 ± 5.6	3.3 ± 0.3	20.4 ± 2.2	76.3 ± 2.5	2.9 ± 0.0	16.1 ± 0.5	81.0 ± 0.5
H222	24.5 ± 2.1	28.7 ± 5.2	46.9 ± 4.2	8.7 ± 0.2	50.7 ± 1.1	40.7 ± 1.3	6.5 ± 0.8	37.7 ± 4.8	55.9 ± 5.6
ClY29-26-3	4.0 ± 2.1	7.5 ± 3.9	88.5 ± 6.0	1.6 ± 0.1	9.7 ± 0.5	88.7 ± 0.6	0.8 ± 0.1	4.6 ± 1.0	94.6 ± 1.1
A101	6.7 ± 1.8	23.5 ± 5.8	69.8 ± 4.7	2.6 ± 0.7	16.1 ± 3.1	81.3 ± 3.8	1.5 ± 0.5	8.3 ± 3.0	90.3 ± 3.5
YAGA	8.3 ± 0.3	16.4 ± 9.3	75.3 ± 9.5	3.5 ± 1.5	20.2 ± 8.0	76.2 ± 9.6	2.3 ± 1.1	13.4 ± 6.6	84.3 ± 7.8
YAPO	9.3 ± 3.7	31.2 ± 9.5	59.5 ± 5.8	5.7 ± 0.3	31.3 ± 1.3	63.0 ± 1.6	5.3 ± 1.1	30.3 ± 5.3	64.4 ± 6.4
YAHO	7.4 ± 5.6	32.5 ± 8.7	60.1 ± 14.0	4.0 ± 2.0	24.7 ± 10.8	71.3 ± 12.8	1.5 ± 0.9	8.5 ± 4.9	89.9 ± 5.8

**Table 3:** ^13^C NMR (600 MHZ, acetone) of compounds **1a**–**7a**.

		Compound					
Atom No.	1a	2a	3a	4a	5a	6a	7a
C=O	188.16	188.44	188.22	188.13	188.44	188.25	189.49
C-α	122.88	122.89	123.08	120.35	120.21	123.27	121.81
C-β	143.95	139.00	143.99	143.91	139.21	144.14	135.37
C-1	136.25	124.73	137.59	128.80	117.66	138.12	106.90
C-1′	132.01	132.19	131.95	132.23	132.48	131.94	132.92
C-2	129.40	159.06	114.05	131.17	161.20	107.21	162.63
C-2′/C-6′	131.63	131.76	131.65	131.48	131.36	131.66	131.16
C-3	129.80	112.35	161.13	115.23	130.93	162.21	91.65
C-3′/C-5′	114.75	114.70	114.73	114.66	114.62	114.54	114.57
C-4	129.80	132.60	122.06	162.58	106.91	103.32	164.32
C-4′	164.53	164.40	164.52	164.33	164.17	164.53	163.96
C-5	129.80	121.56	130.77	115.23	164.18	162.21	91.65
C-6	129.40	129.37	117.10	131.17	99.02	107.21	162.63
C-2 OCH_3_	—	56.06	—	—	56.12	—	56.36
C-3 OCH_3_	—	—	55.96	—	—	55.83	—
C-4 OCH_3_	—	—	—	55.78	—	—	55.86
C-4′ OCH_3_	55.97	55.93	55.70	55.93	56.09	55.97	55.88
C-5 OCH_3_	—	—	—	—	55.89	55.83	—
C-6 OCH_3_	—	—	—	—	—	—	56.36

**Table 4:** ^13^C NMR (600 MHZ, acetone) of compounds **1c**–**7c**.

		Compound				
Atom No.	1c	2c	3c	4c	5c	6c
C=O	197.75	198.21	197.76	197.87	198.36	197.80
C-α	40.41	39.00	40.31	40.67	39.30	40.26
C-β	30.89	26.32	30.93	30.03	25.77	31.22
C-1	142.71	130.44	144.24	134.47	131.09	144.99
C-1′	131.06	131.06	131.02	131.05	122.5	131.06
C-2	129.33	158.52	115.02	130.21	159.30	107.32
C-2′/C-6′	131.06	131.06	131.04	131.02	131.04	131.06
C-3	129.18	111.23	160.78	114.56	–	161.92
C-3′/C-5′	114.57	114.57	114.55	114.54	114.54	114.57
C-4	126.70	128.25	121.51	158.97	105.09	98.59
C-4′	164.41	164.36	164.37	164.34	164.31	164.40
C-5	129.18	121.21	130.12	114.56	160.59	161.92
C-6	129.33	130.73	112.09	130.21	130.99	107.32
C-2 OCH_3_	-	55.63	–	–	55.67	–
C-3 OCH_3_	-	–	55.33	–	–	55.46
C-4 OCH_3_	-	–	–	55.41	–	–
C-4′ OCH_3_	55.91	55.90	55.89	55.88	55.89	55.91
C-5 OCH_3_	-	–	–	–	55.54	55.46
C-6 OCH_3_	-	–	–	–	–	–

## Data Availability

Data are contained within this article and Supplementary Materials.

## Supplementary Materials

The following supporting information can be downloaded at: https://www.mdpi.com/article/10.3390/molecules31061049/s1. Figures S1–S20 (UV-Vis Spectra of chalcones and dihydrochalcones); Figures S21–S84 (NMR spectra of chalcones and dihydrochalcones); Figure S85. Time-course of the biotransformation of 1a to 1c; Table S1. Comparative analysis of identified *Yarrowia* OYE sequences retrieved from the WGS database, showing high structural conservation across the clade.
