# Supplementary material for: Whole-Cell Biocatalysis for the Production of Structurally Diverse Methoxydihydrochalcones: Broad Activity of the Yarrowia Clade
Source: Molecules. 2026 Mar 22;31(6):1049. doi: 10.3390/molecules31061049 (PMC13029650; doi:10.3390/molecules31061049)
Supplement: Supplementary file 1 [file molecules-31-01049-s001.zip › molecules-4185495-supplementary.pdf]

Supplementary materials

Whole-Cell Biocatalysis for the Production of Structurally Diverse Methoxydihydrochalcones: Broad Activity of the Yarrowia Clade and Application of Yarrowia lipolytica KCh 71

Paweł Chlipała [1,*], Marcelina Mazur [1], Anna Kancelista [2], Zbigniew Lazar [2] and Tomasz Janeczko [1,*]

[1]Department of Food Chemistry and Biocatalysis, Wrocław University of Environmental and Life Sciences,
50-375 Wrocław, Poland; marcelina.mazur@upwr.edu.pl (M.M.)

[2]Department of Biotechnology and Food Microbiology, Wrocław University of Environmental and Life Sciences,
51-630 Wrocław, Poland; anna.kancelista@upwr.edu.pl (A.K.), zbigniew.lazar@upwr.edu.pl (Z.L.)
*Correspondence: pawel.chlipala@upwr.edu.pl (P.C.), tomasz.janeczko@upwr.edu.pl (T.J)

Figure S1 UV-Vis spectrum of *trans*-4′-methoxychalcone (**1a**). 3
Figure S2 UV-Vis spectrum of *cis*-4′-methoxychalcone (**1b**). 3
Figure S3 UV-Vis spectrum of 4′-methoxydihydrochalcone (**1c**). 4
Figure S4 UV-Vis spectrum of *trans*-2,4′-dimethoxychalcone (**2a**). 4
Figure S5 UV-Vis spectrum of *cis*-2,4′-dimethoxychalcone (**2b**). 5
Figure S6 UV-Vis spectrum of 2,4′-dimethoxydihydrochalcone (**2c**). 5
Figure S7 UV-Vis spectrum of *trans*-3,4′-dimethoxychalcone (**3a**). 6
Figure S8 UV-Vis spectrum of *cis*-3,4′-dimethoxychalcone (**3b**). 6
Figure S9 UV-Vis spectrum of 3,4′-dimethoxydihydrochalcone (**3c**). 7
Figure S10 UV-Vis spectrum of *trans*-4,4′-dimethoxychalcone (**4a**). 7
Figure S 11 UV-Vis spectrum of *cis*-4,4′-dimethoxychalcone (**4b**). 8
Figure S 12 UV-Vis spectrum of 4,4′-dimethoxydihydrochalcone (**4c**). 8
Figure S13 UV-Vis spectrum of *trans*-2,5,4′-trimethoxychalcone (**5a**). 9
Figure S14 UV-Vis spectrum of *cis*-2,5,4′-trimethoxychalcone (**5b**). 9
Figure S15 UV-Vis spectrum of 2,5,4′-trimethoxydihydrochalcone (**5c**). 10
Figure S16 UV-Vis spectrum of *trans*-3,5,4′-trimethoxychalcone (**6a**). 10
Figure S17 UV-Vis spectrum of *cis*-3,5,4′-trimethoxychalcone (**6b**). 11
Figure S18 UV-Vis spectrum of 3,5,4′-trimethoxydihydrochalcone (**6c**). 11
Figure S19 UV-Vis spectrum of *trans*-2,4,6,4′-tetramethoxychalcone (**7a**). 12
Figure S20 UV-Vis spectrum of *cis*-2,4,6,4′-tetramethoxychalcone (**7b**). 12
Figure S21 ^{1}H NMR spectrum of 4′-methoxychalcone (**1a**) (400 MHZ; acetone-d$_6$). 13
Figure S22 ^{13}C NMR spectrum of 4′-methoxychalcone (**1a**) (400 MHZ; acetone-d$_6$). 13
Figure S23 COSY NMR spectrum of 4′-methoxychalcone (**1a**) (400 MHZ; acetone-d$_6$). 14
Figure S24 HSQC NMR spectrum of 4′-methoxychalcone (**1a**) (400 MHZ; acetone-d$_6$). 14
Figure S25 HMBC NMR spectrum of 4′-methoxychalcone (**1a**) (400 MHZ; acetone-d$_6$). 15
Figure S26 ^{1}H NMR spectrum of 2,4′-dimethoxychalcone (**2a**)(600 MHZ; acetone-d$_6$). 15
Figure S 27 ^{13}C NMR spectrum of 2,4′-dimethoxychalcone (**2a**) (600 MHZ; acetone-d$_6$). 16
Figure S28 HSQC NMR spectrum of 2,4′-dimethoxychalcone (**2a**) (600 MHZ; acetone-d$_6$). 16
Figure S29 HMBC NMR spectrum of 2,4′-dimethoxychalcone (**2a**) (600 MHZ; acetone-d$_6$). 17
Figure S30 ^{1}H NMR spectrum of 3,4'-dimethoxychalcone (**3a**) (600 MHz; acetone-d$_6$). 17
Figure S31 ^{13}C NMR spectrum of 3,4'-dimethoxychalcone (**3a**) (600 MHz; acetone-d$_6$). 18
Figure S32 COSY NMR spectrum of 3,4'-dimethoxychalcone (**3a**) (600 MHz; acetone-d$_6$). 18
Figure S33 HSQC NMR spectrum of 3,4'-dimethoxychalcone (**3a**) (600 MHz; acetone-d$_6$). 19
Figure S34 HMBC NMR spectrum of 3,4'-dimethoxychalcone (600 MHz; acetone-d6). 19
Figure S35 ^{1}H NMR spectrum of 4,4'-dimethoxychalcone (**4a**) (600 MHz; acetone-d$_6$). 20
Figure S36 ^{13}C NMR spectrum of 4,4'-dimethoxychalcone (**4a**) (600 MHz; acetone-d$_6$). 20
Figure S37 COSY NMR spectrum of 4,4'-dimethoxychalcone (**4a**) (600 MHz; acetone-d$_6$). 21
Figure S38 HSQC NMR spectrum of 4,4'-dimethoxychalcone (600 MHz; acetone-d6). 21
Figure S39 HMBC NMR spectrum of 4,4'-dimethoxychalcone (**4a**) (600 MHz; acetone-d$_6$). 22
Figure S40 ^{1}H NMR spectrum of 2,5,4'-trimethoxychalcone (**5a**) (600 MHz; acetone-d$_6$). 22
Figure S41 ^{13}C NMR spectrum of 2,5,4'-trimethoxychalcone (**5a**) (600 MHz; acetone-d$_6$). 23

Figure S42 COSY NMR spectrum of 2,5,4'-trimethoxychalcone (**5a**) (600 MHz; acetone-d_6). 23
Figure S43 HSQC NMR spectrum of 2,5,4'-trimethoxychalcone (**5a**) (600 MHz; acetone-d_6). 24
Figure S44 HMBC NMR spectrum of 2,5,4'-trimethoxychalcone (**5a**) (600 MHz; acetone-d_6). 24
Figure S45 ^{1}H NMR spectrum of 3,5,4'-trimethoxychalcone (**6a**) (600 MHz; acetone-d_6). 25
Figure S46 ^{13}C NMR spectrum of 3,5,4'-trimethoxychalcone (**6a**) (600 MHz; acetone-d_6). 25
Figure S47 COSY NMR spectrum of 3,5,4'-trimethoxychalcone (**6a**) (600 MHz; acetone-d_6). 26
Figure S48 HSQC NMR spectrum of 3,5,4'-trimethoxychalcone (**6a**) (600 MHz; acetone-d_6). 26
Figure S49 HMBC NMR spectrum of 3,5,4'-trimethoxychalcone (**6a**) (600 MHz; acetone-d_6). 27
Figure S50 ^{1}H NMR spectrum of 2,4,6,4'-tetramethoxychalcone (**7a**) (600 MHz; acetone-d_6). 27
Figure S51 ^{13}C NMR spectrum of 2,4,6,4'-tetramethoxychalcone (**7a**) (600 MHz; acetone-d_6). 28
Figure S52 COSY NMR spectrum of 2,4,6,4'-tetramethoxychalcone (**7a**) (600 MHz; acetone-d_6). 28
Figure S53 HSQC NMR spectrum of 2,4,6,4'-tetramethoxychalcone (**7a**) (600 MHz; acetone-d_6). 29
Figure S54 HMBC NMR spectrum of 2,4,6,4'-tetramethoxychalcone (**7a**) (600 MHz; acetone-d_6). 29
Figure S55 ^{1}H NMR spectrum of 4'-methoxydihydrochalcone (**1c**) (600 MHz; acetone-d_6). 30
Figure S56 ^{13}C NMR spectrum of 4'-methoxydihydrochalcone (**1c**) (600 MHz; acetone-d_6). 30
Figure S57 COSY NMR spectrum of 4'-methoxydihydrochalcone (**1c**) (600 MHz; acetone-d6). 31
Figure S58 HSQC NMR spectrum of 4'-methoxydihydrochalcone (**1c**) (600 MHz; acetone-d_6). 31
Figure S59 HMBC NMR spectrum of 4'-methoxydihydrochalcone (**1c**) (600 MHz; acetone-d_6). 32
Figure S60 ^{1}H NMR spectrum of 2,4'-dimethoxydihydrochalcone (**2c**) (600 MHz; acetone-d_6). 32
Figure S61 ^{13}C NMR spectrum of 2,4'-dimethoxydihydrochalcone (**2c**) (600 MHz; acetone-d_6). 33
Figure S62 COSY NMR spectrum of 2,4'-dimethoxydihydrochalcone (**2c**) (600 MHz; acetone-d_6). 33
Figure S63 HSQC NMR spectrum of 2,4'-dimethoxydihydrochalcone (**2c**) (600 MHz; acetone-d_6). 34
Figure S64 HMBC NMR spectrum of 2,4'-dimethoxydihydrochalcone (**2c**) (600 MHz; acetone-d_6). 34
Figure S65 ^{1}H NMR spectrum of 3,4'-dimethoxydihydrochalcone (**3c**) (600 MHz; acetone-d_6). 35
Figure S66 ^{13}C NMR spectrum of 3,4'-dimethoxydihydrochalcone (**3c**) (600 MHz; acetone-d_6). 35
Figure S67 COSY NMR spectrum of 3,4'-dimethoxydihydrochalcone (**3c**) (600 MHz; acetone-d_6). 36
Figure S68 HSQC NMR spectrum of 3,4'-dimethoxydihydrochalcone (**3c**) (600 MHz; acetone-d_6). 36
Figure S69 HMBC NMR spectrum of 3,4'-dimethoxydihydrochalcone (**3c**) (600 MHz; acetone-d_6). 37
Figure S70 ^{1}H NMR spectrum of 4,4'-dimethoxydihydrochalcone (**4c**) (600 MHz; acetone-d_6). 37
Figure S71 ^{13}C NMR spectrum of 4,4'-dimethoxydihydrochalcone (**4c**) (600 MHz; acetone-d_6). 38
Figure S72 COSY NMR spectrum of 4,4'-dimethoxydihydrochalcone (**4c**) (600 MHz; acetone-d_6). 38
Figure S73 HSQC NMR spectrum of 4,4'-dimethoxydihydrochalcone (**4c**) (600 MHz; acetone-d_6). 39
Figure S74 HMBC NMR spectrum of 4,4'-dimethoxydihydrochalcone (**4c**) (600 MHz; acetone-d_6). 39
Figure S75 ^{1}H NMR spectrum of 2,5,4'-trimethoxydihydrochalcone (**5c**) (600 MHz; acetone-d_6). 40
Figure S76 ^{13}C NMR spectrum of 2,5,4'-trimethoxydihydrochalcone (**5c**) (600 MHz; acetone-d_6). 40
Figure S77 COSY NMR spectrum of 2,5,4'-trimethoxydihydrochalcone (**5c**) (600 MHz; acetone-d_6). 41
Figure S78 HSQC NMR spectrum of 2,5,4'-trimethoxydihydrochalcone (**5c**) (600 MHz; acetone-d_6). 41
Figure S79 HMBC NMR spectrum of 2,5,4'-trimethoxydihydrochalcone (**5c**) (600 MHz; acetone-d_6). 42
Figure S80 ^{1}H NMR spectrum of 3,5,4'-trimethoxydihydrochalcone (**6c**) (600 MHz; acetone-d_6). 42
Figure S81 ^{13}C NMR spectrum of 3,5,4'-trimethoxydihydrochalcone (**6c**) (600 MHz; acetone-d_6). 43
Figure S82 COSY NMR spectrum of 3,5,4'-trimethoxydihydrochalcone (**6c**) (600 MHz; acetone-d_6). 43
Figure S83 HSQC NMR spectrum of 3,5,4'-trimethoxydihydrochalcone (**6c**) (600 MHz; acetone-d_6). 44
Figure S84 HMBC NMR spectrum of 3,5,4'-trimethoxydihydrochalcone (**6c**) (600 MHz; acetone-d_6). 44
Figure S85 Time-course of the biotransformation of **1a** to **1c**. Compound **1b** represents a photo-induced isomer of **1a**, formed independently of microbial activity. 45
Table S1 Comparative analysis of identified *Yarrowia* OYE sequences retrieved from the WGS database, showing high structural conservation across the clade. 46

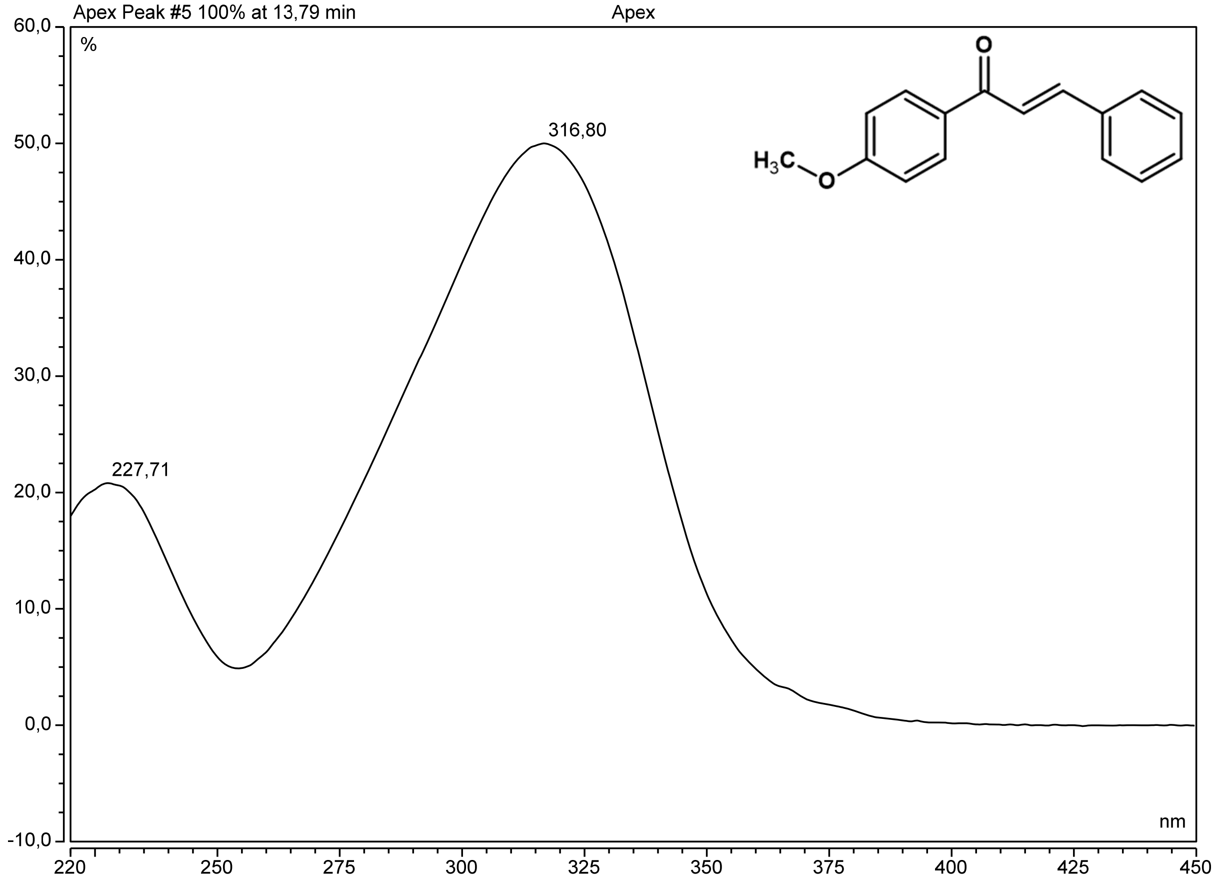

Figure S1 UV-Vis spectrum of *trans*-4′-methoxychalcone (**1a**).

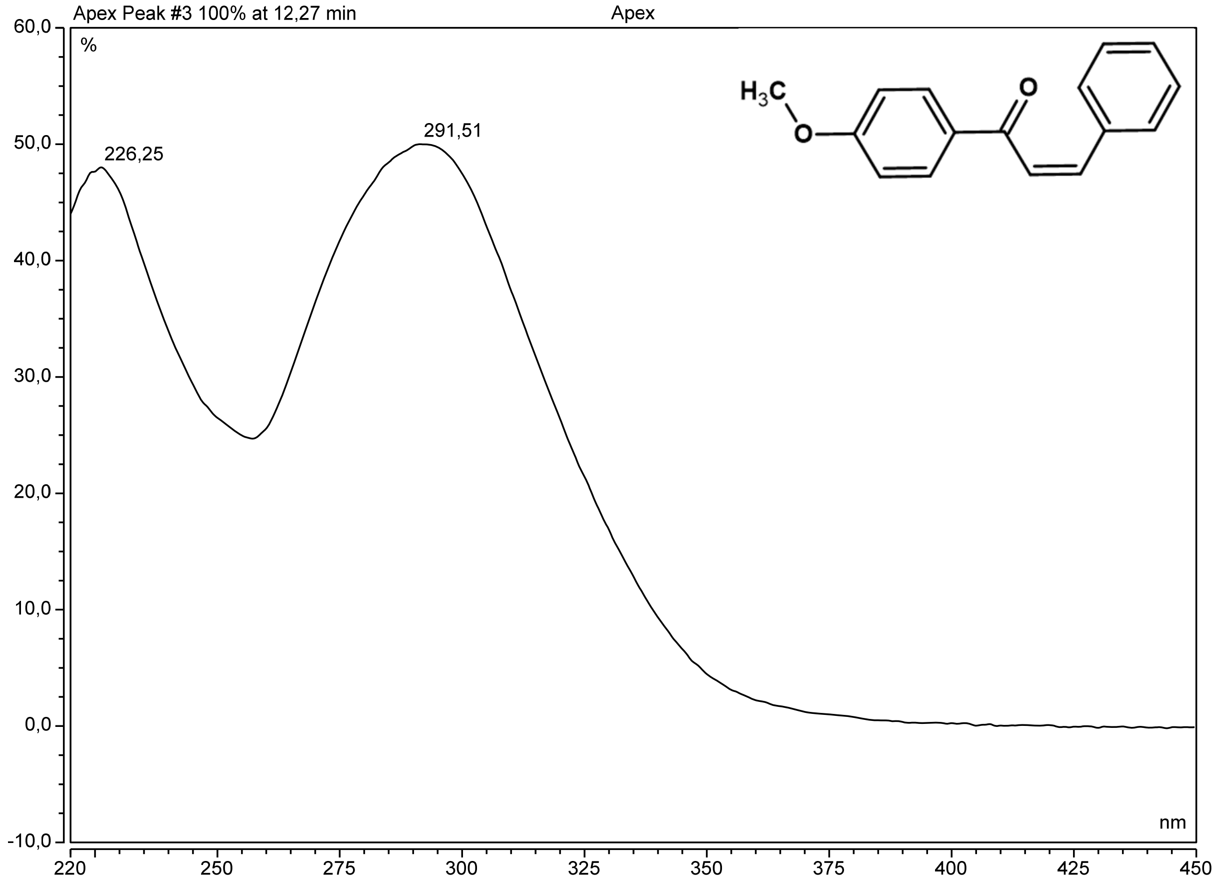

Figure S2 UV-Vis spectrum of *cis*-4′-methoxychalcone (**1b**).

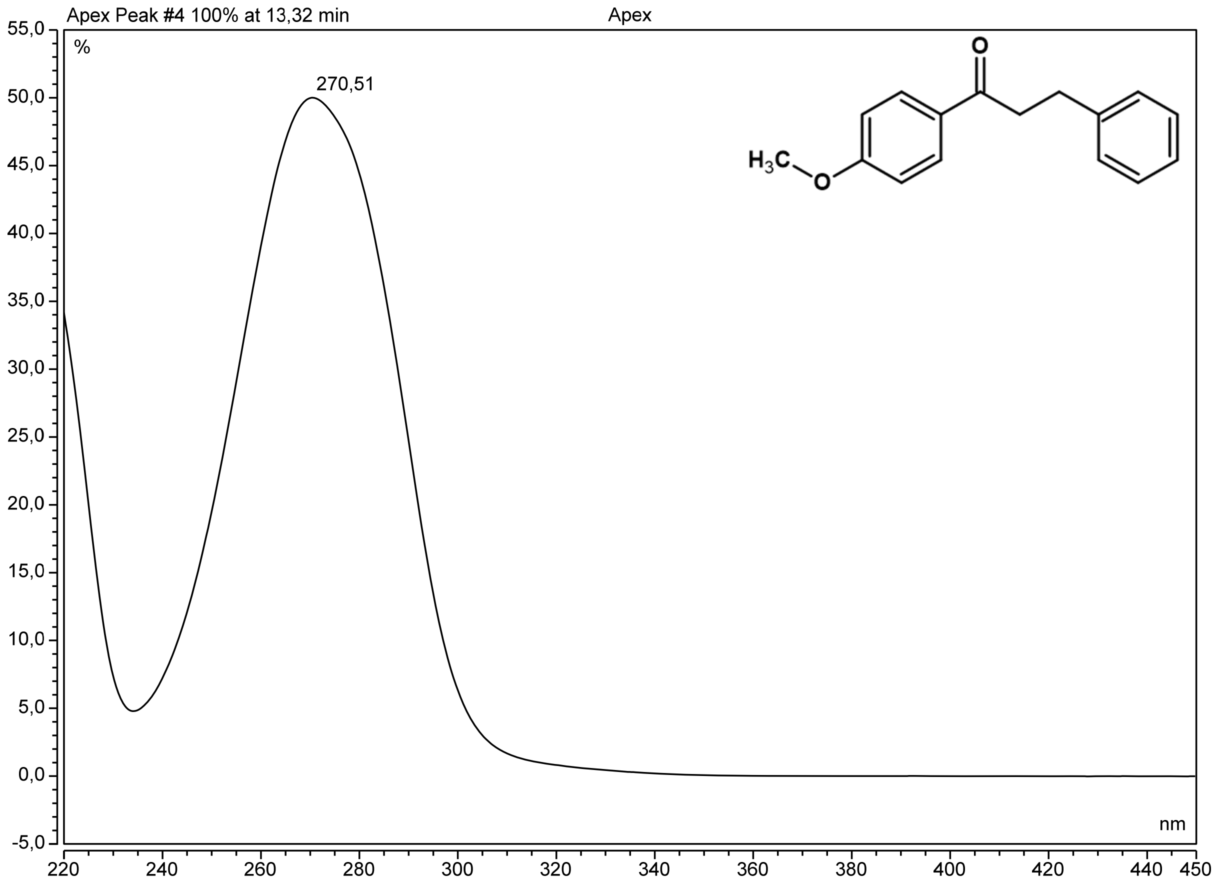

Figure S3 UV-Vis spectrum of 4′-methoxydihydrochalcone (**1c**).

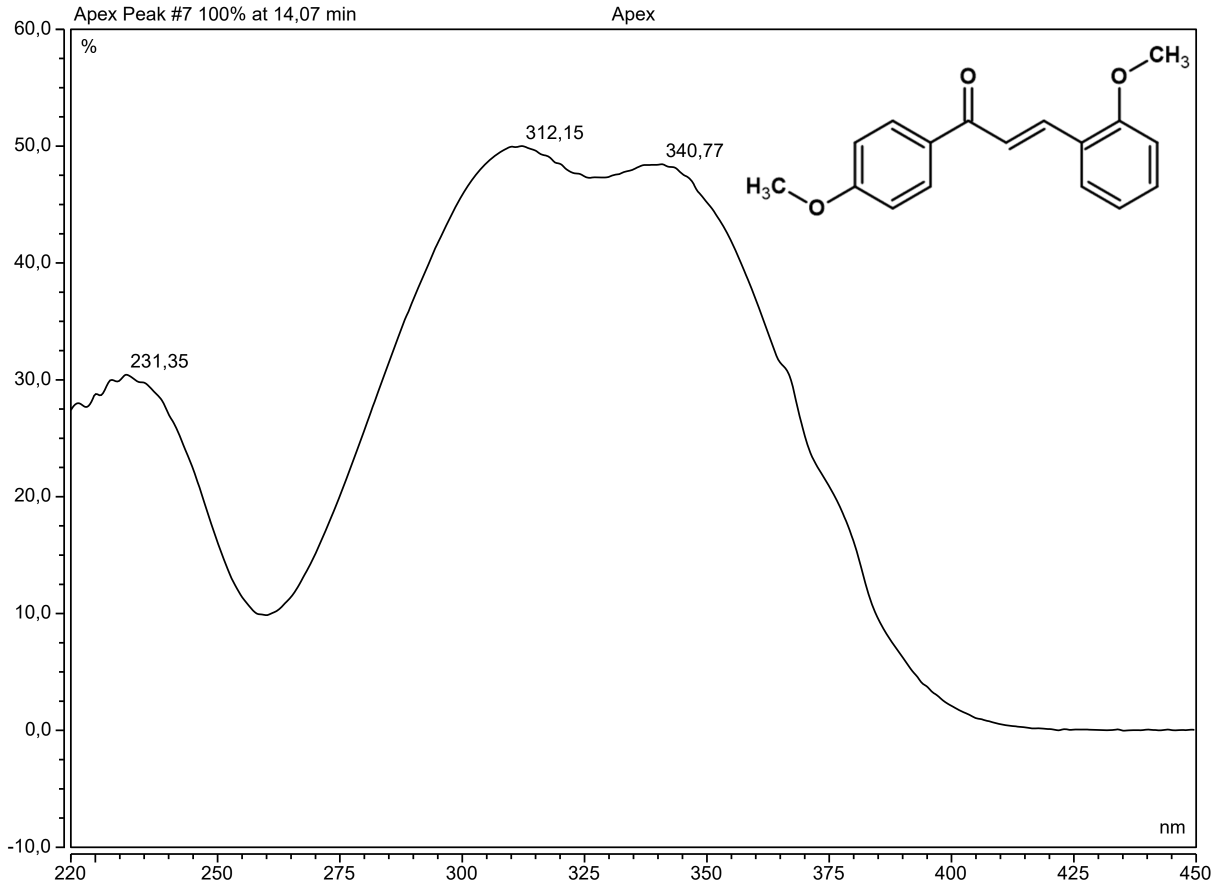

Figure S4 UV-Vis spectrum of *trans*-2,4′-dimethoxychalcone (**2a**).

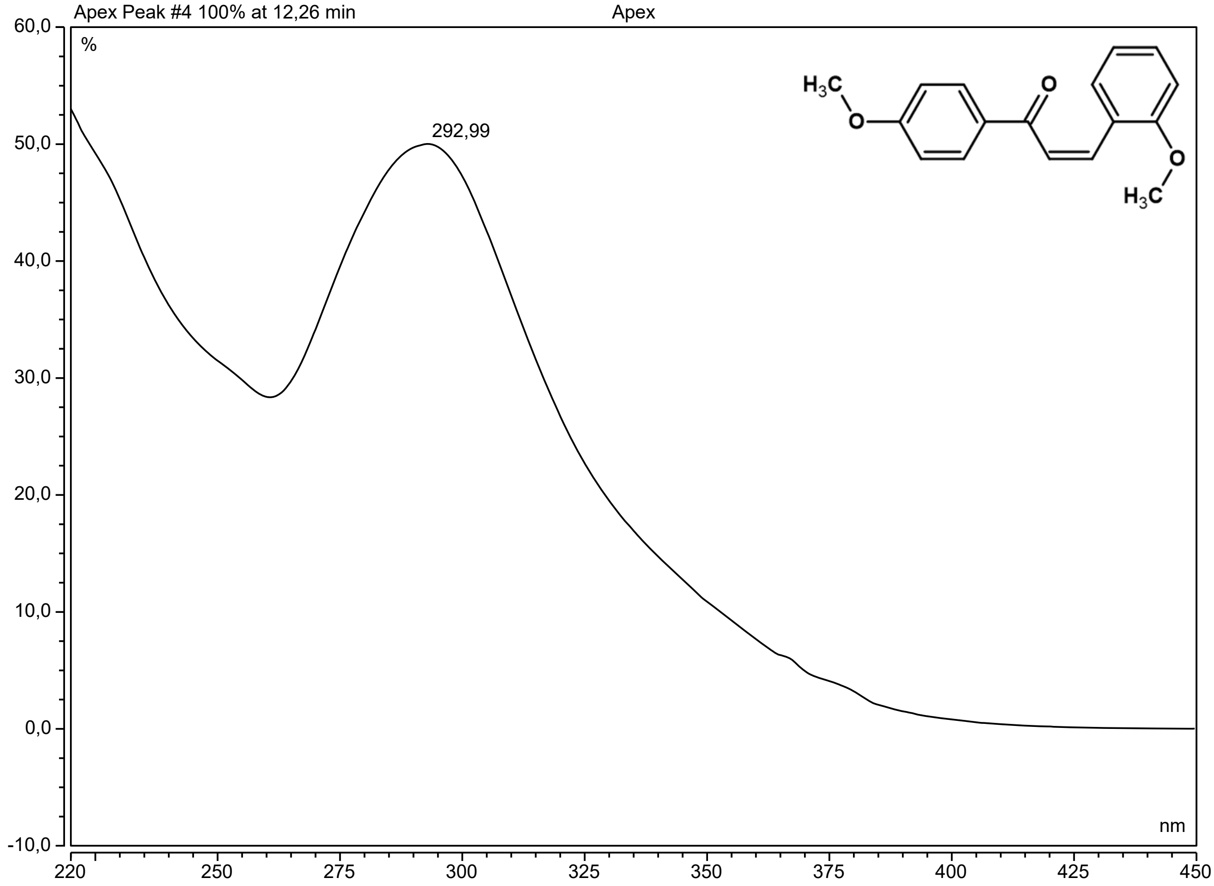

Figure S5 UV-Vis spectrum of *cis*-2,4′-dimethoxychalcone (**2b**).

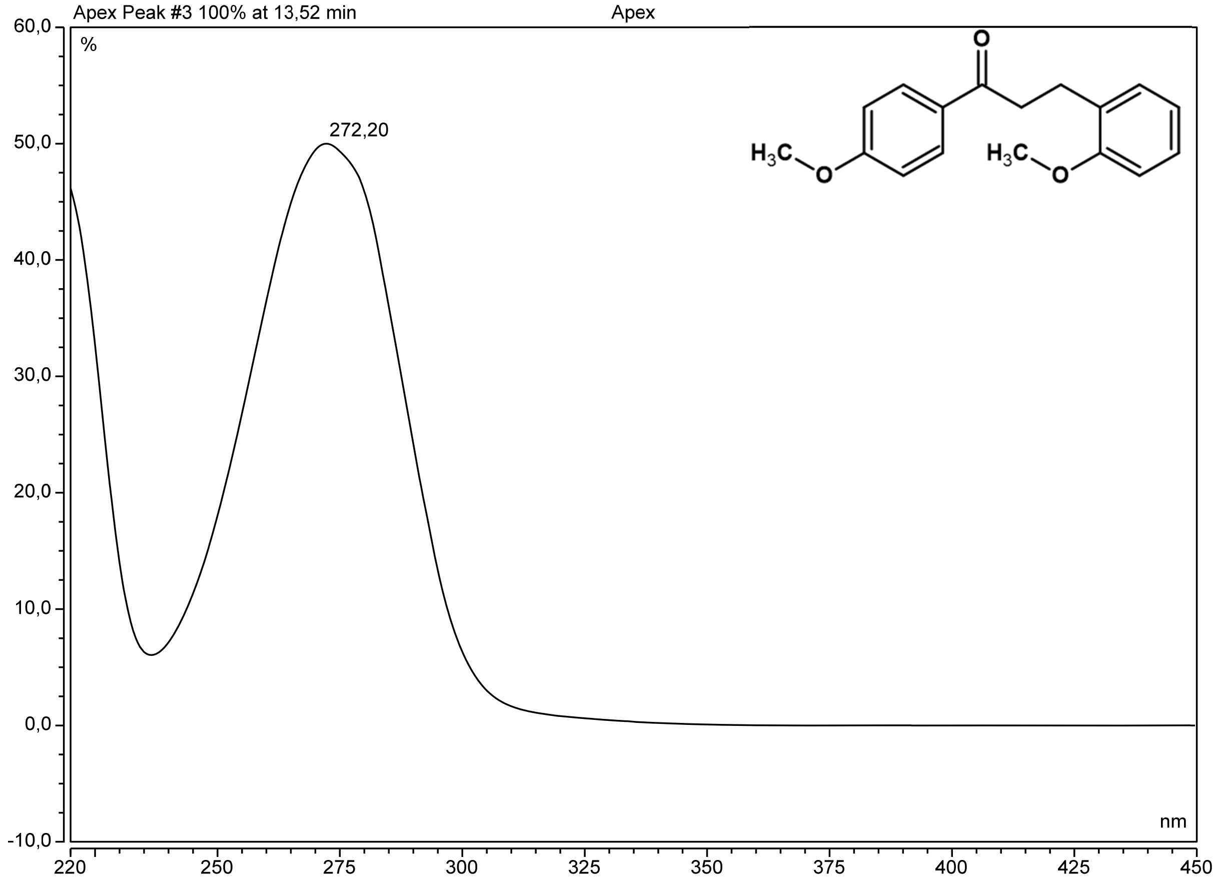

Figure S6 UV-Vis spectrum of 2,4′-dimethoxydihydrochalcone (**2c**).

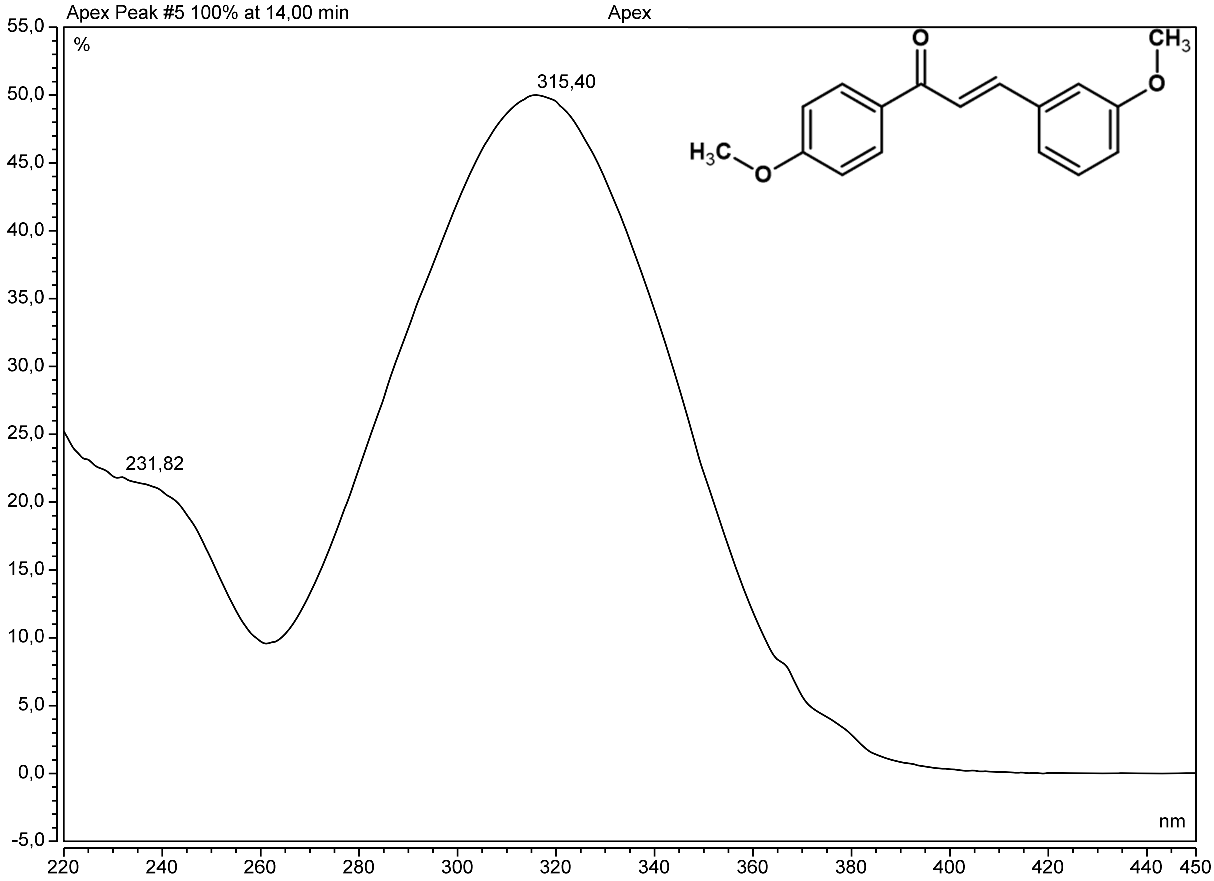

Figure S7 UV-Vis spectrum of *trans*-3,4′-dimethoxychalcone (**3a**).

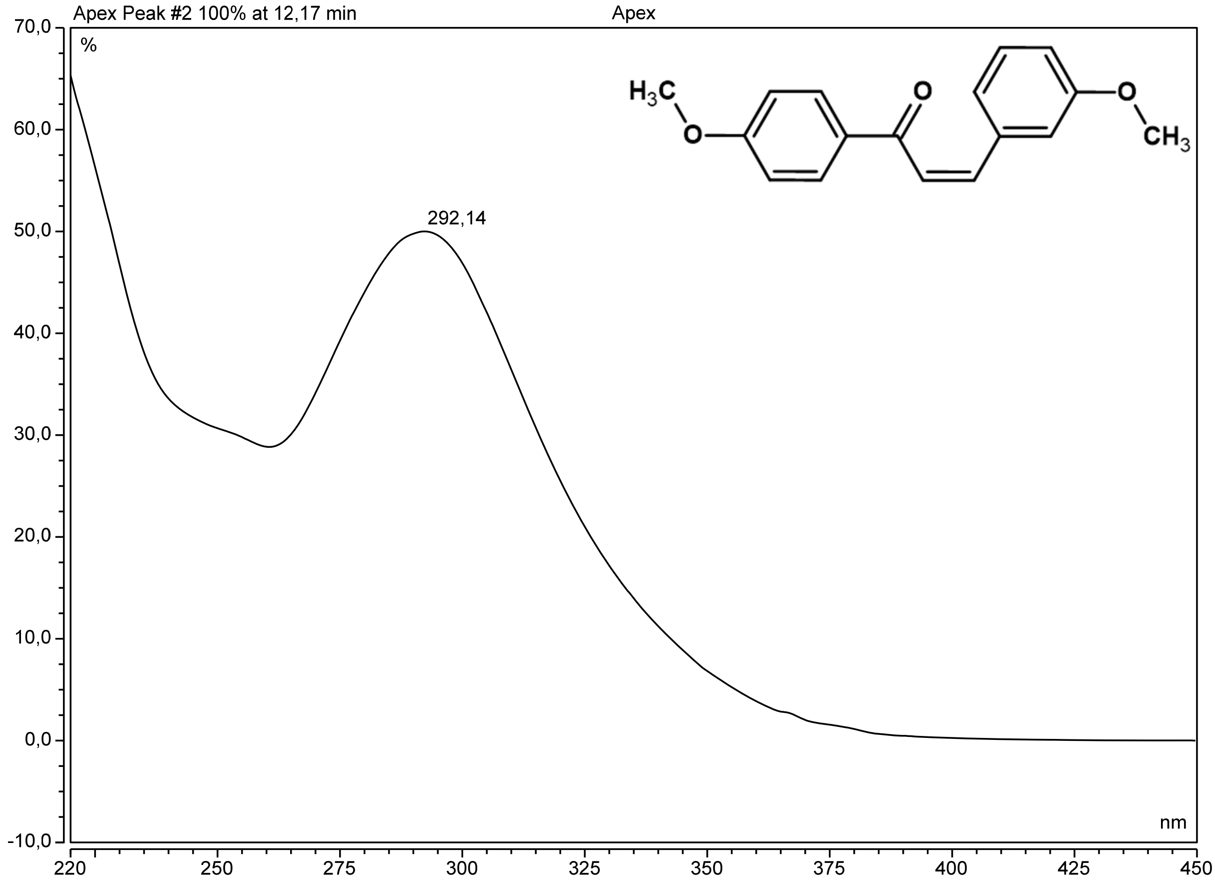

Figure S8 UV-Vis spectrum of *cis*-3,4′-dimethoxychalcone (**3b**).

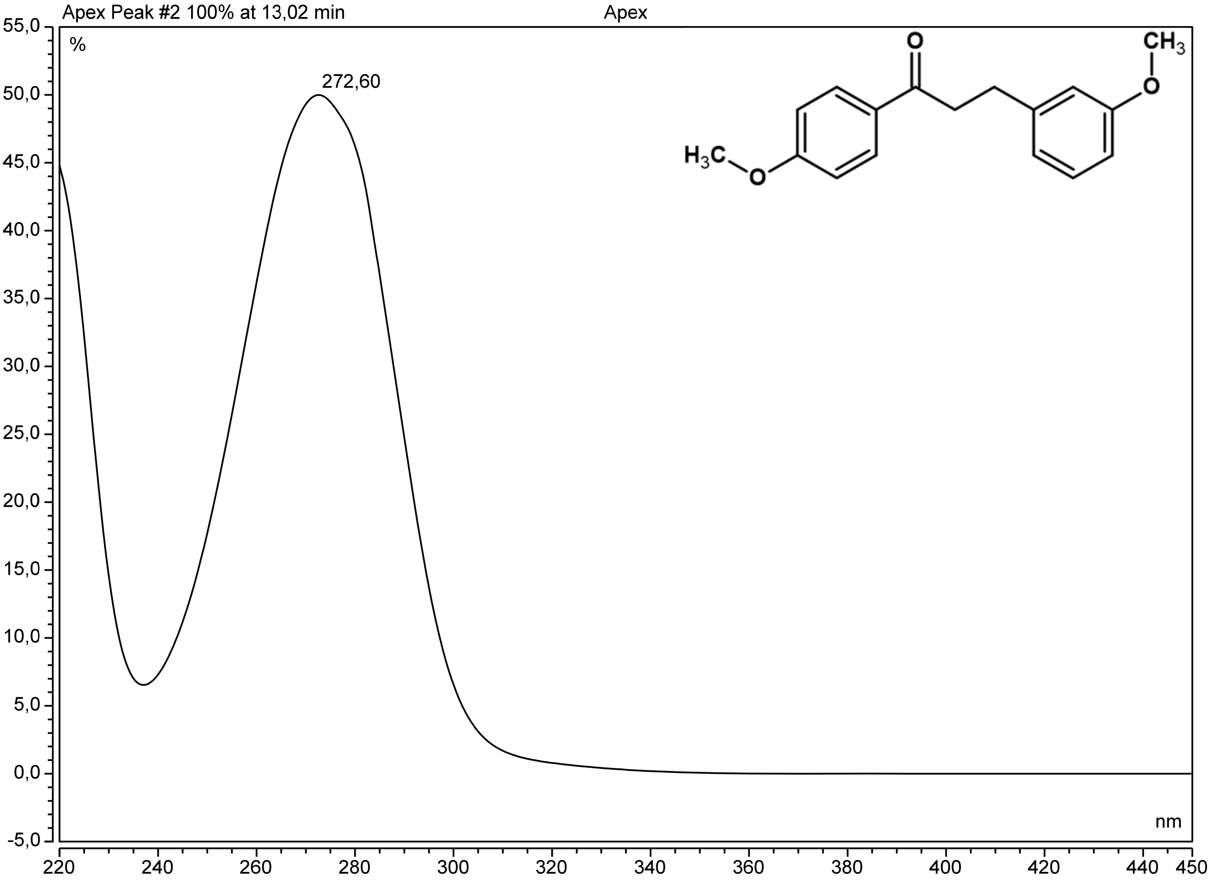

Figure S9 UV-Vis spectrum of 3,4′-dimethoxydihydrochalcone (**3c**).

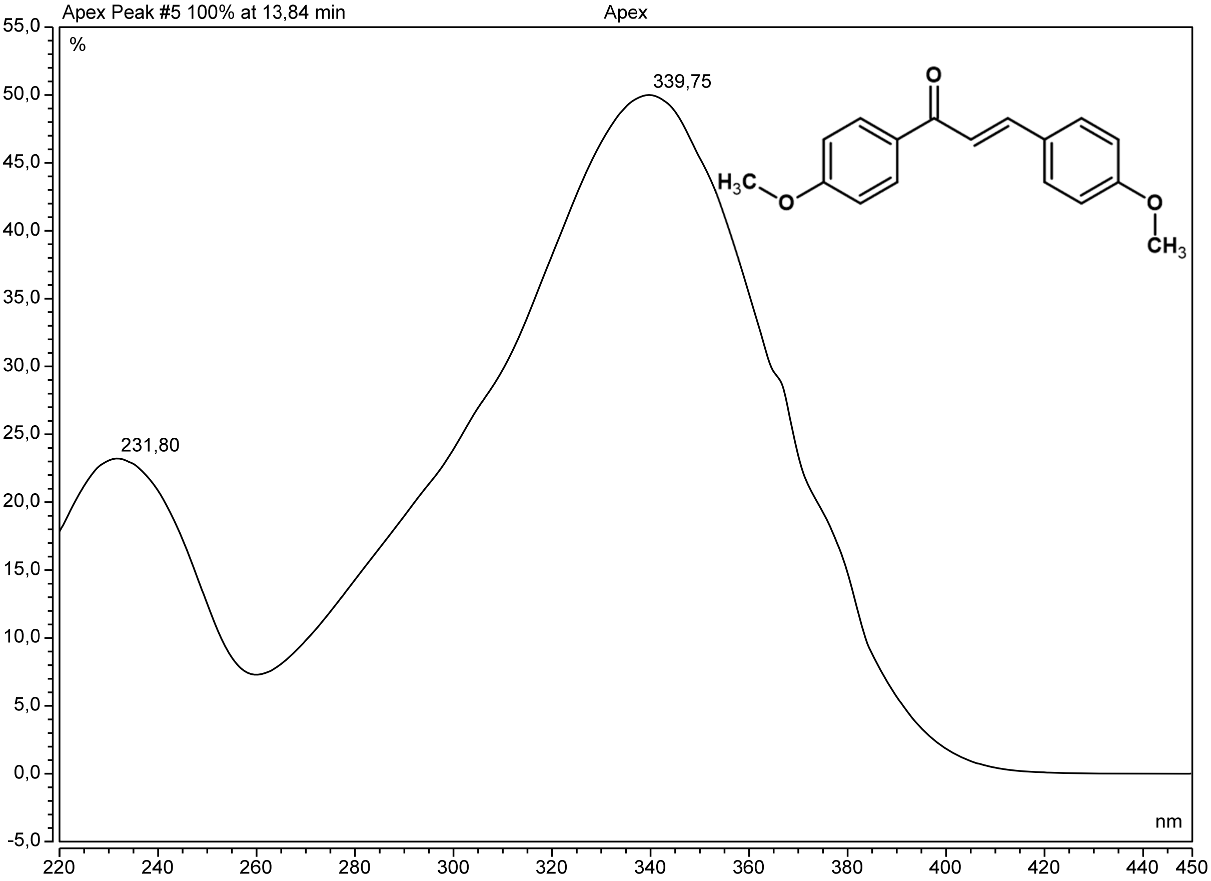

Figure S10 UV-Vis spectrum of *trans*-4,4′-dimethoxychalcone (**4a**).

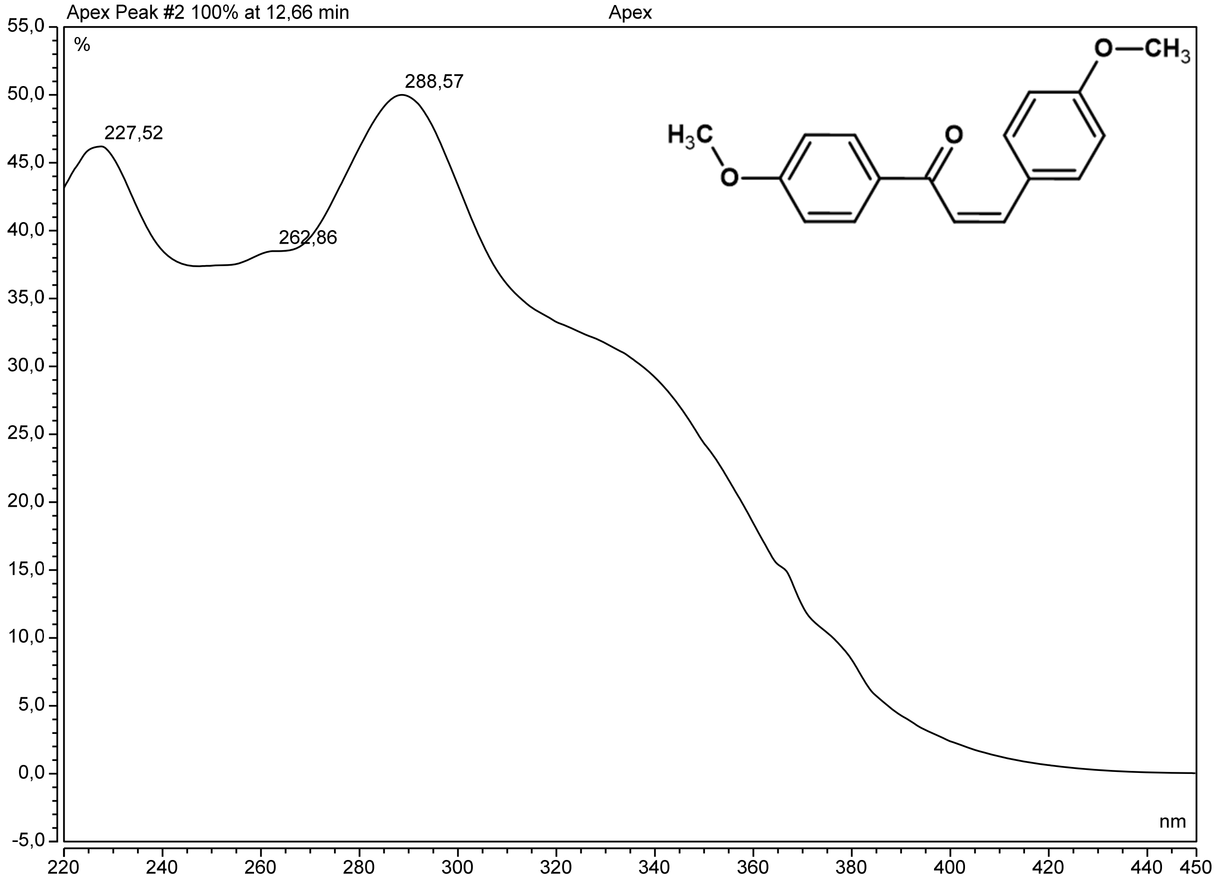

Figure S 11 UV-Vis spectrum of *cis*-4,4′-dimethoxychalcone (**4b**).

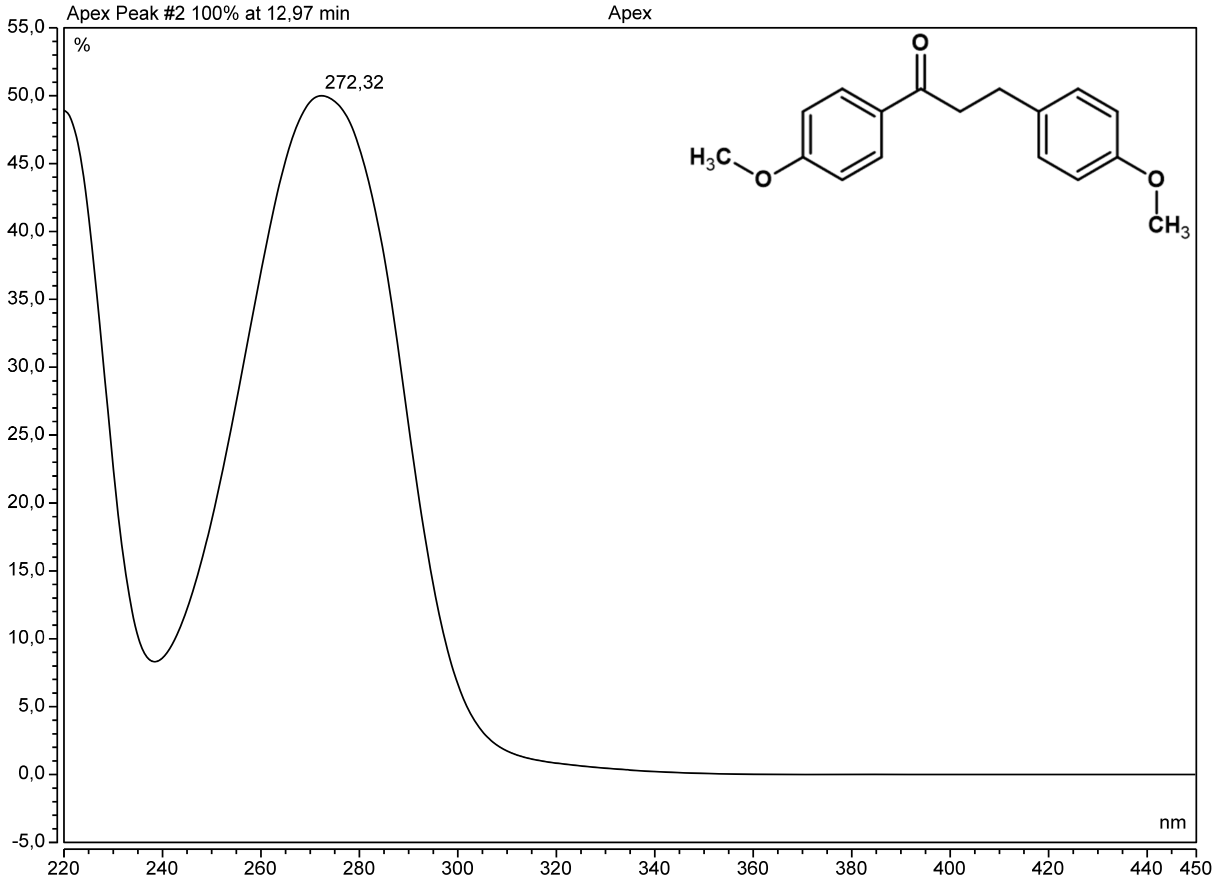

Figure S 12 UV-Vis spectrum of 4,4′-dimethoxydihydrochalcone (**4c**).

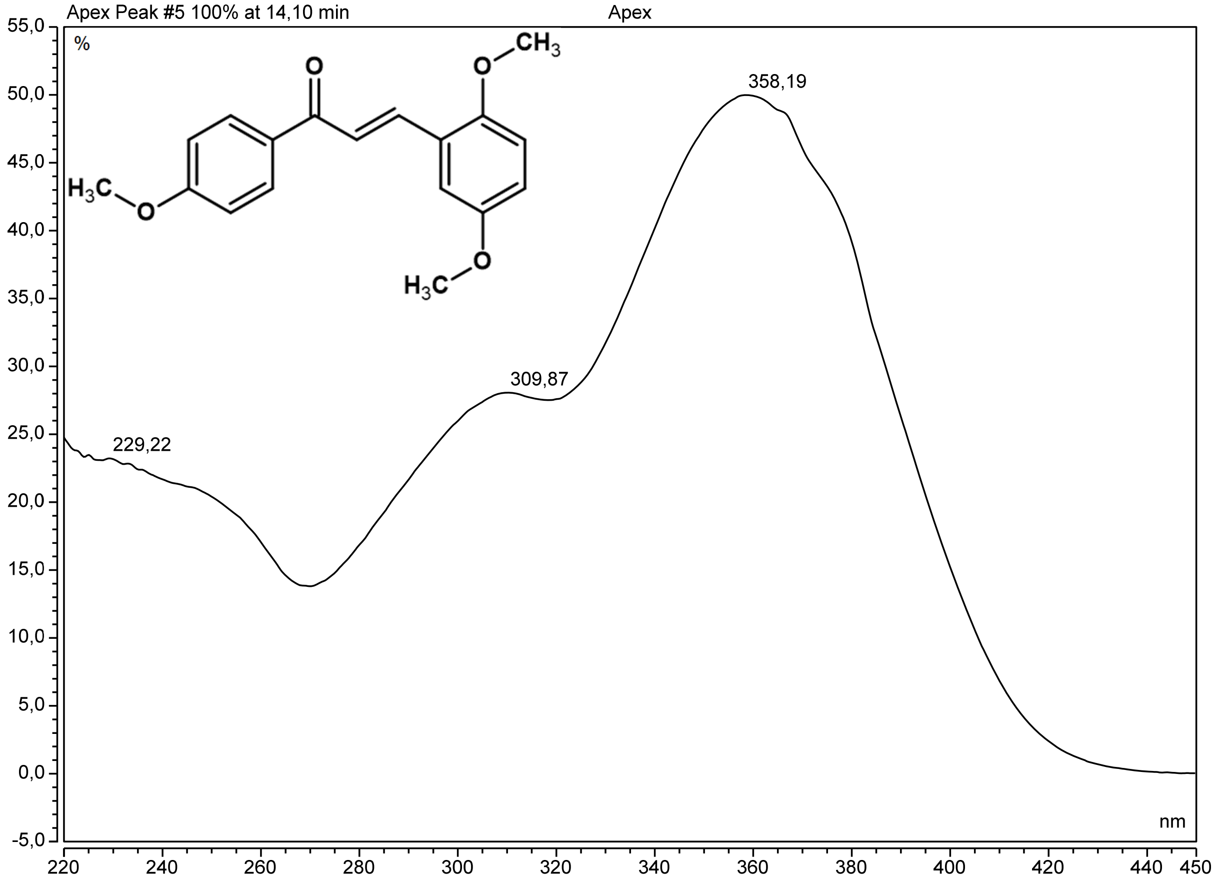

Figure S13 UV-Vis spectrum of *trans*-2,5,4′-trimethoxychalcone (**5a**).

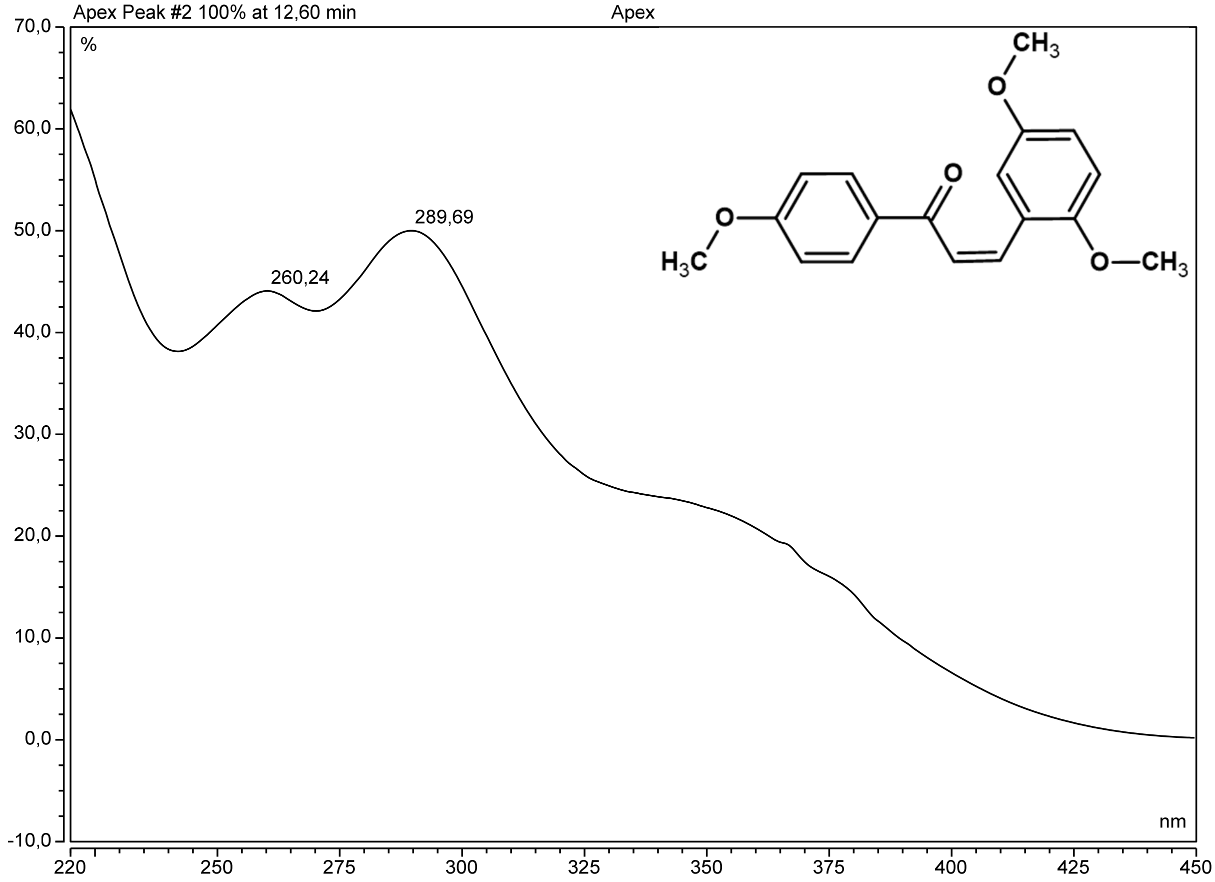

Figure S14 UV-Vis spectrum of *cis*-2,5,4′-trimethoxychalcone (**5b**).

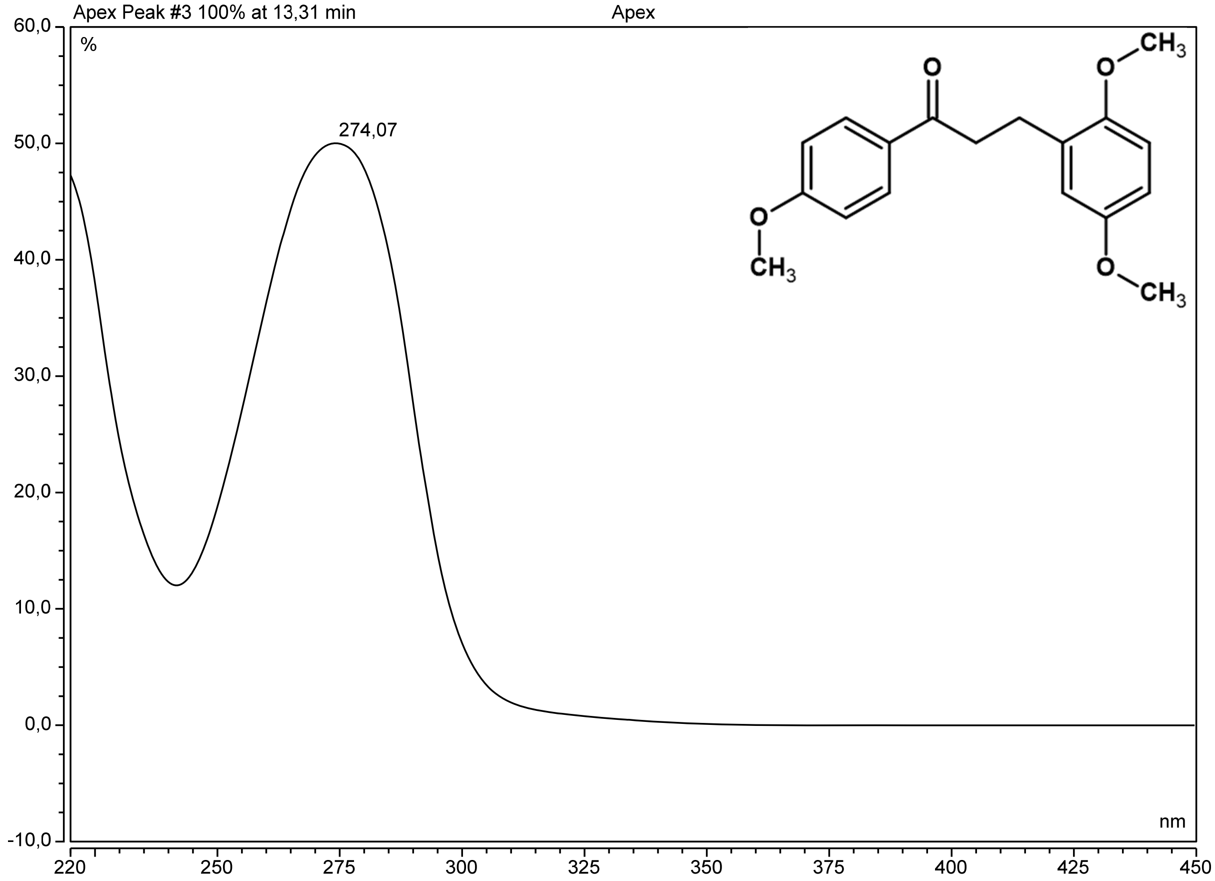

Figure S15 UV-Vis spectrum of 2,5,4′-trimethoxydihydrochalcone (**5c**).

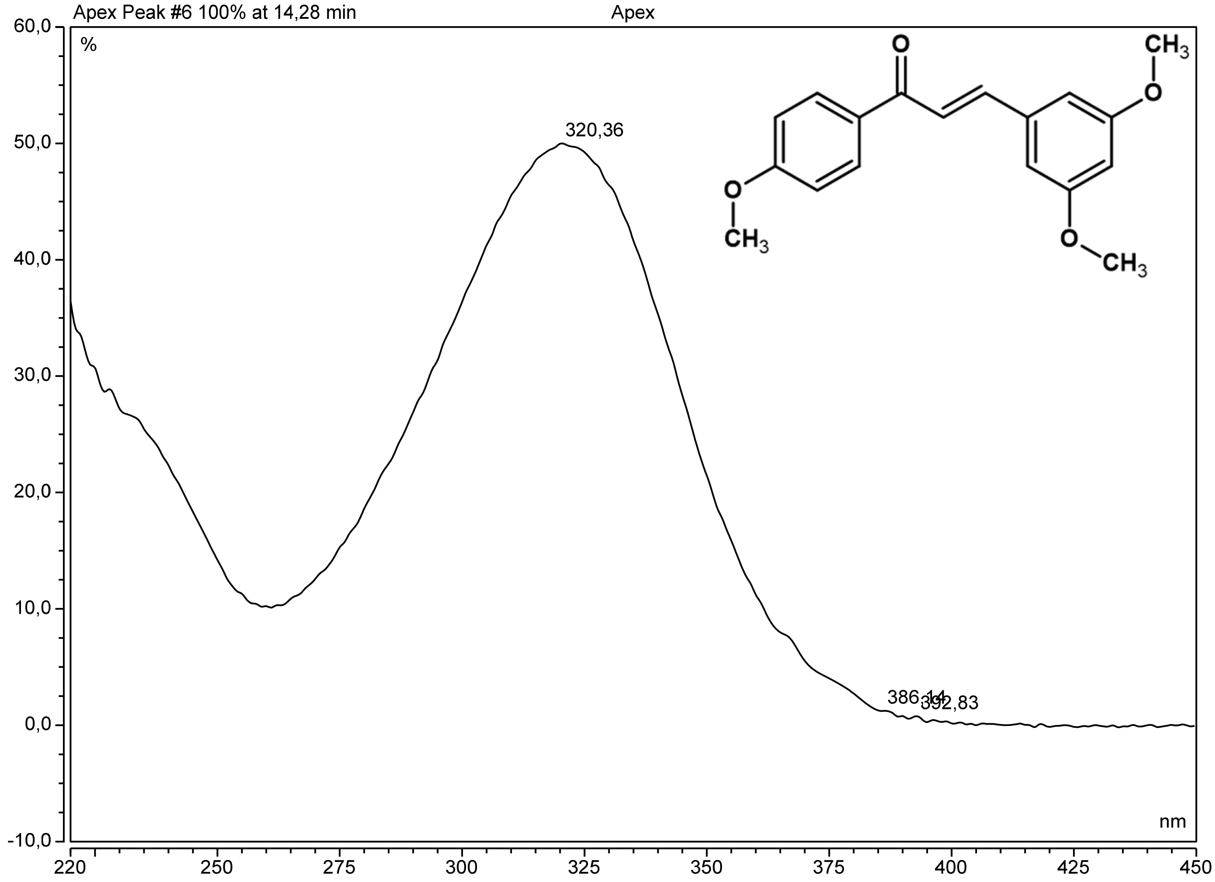

Figure S16 UV-Vis spectrum of *trans*-3,5,4′-trimethoxychalcone (**6a**).

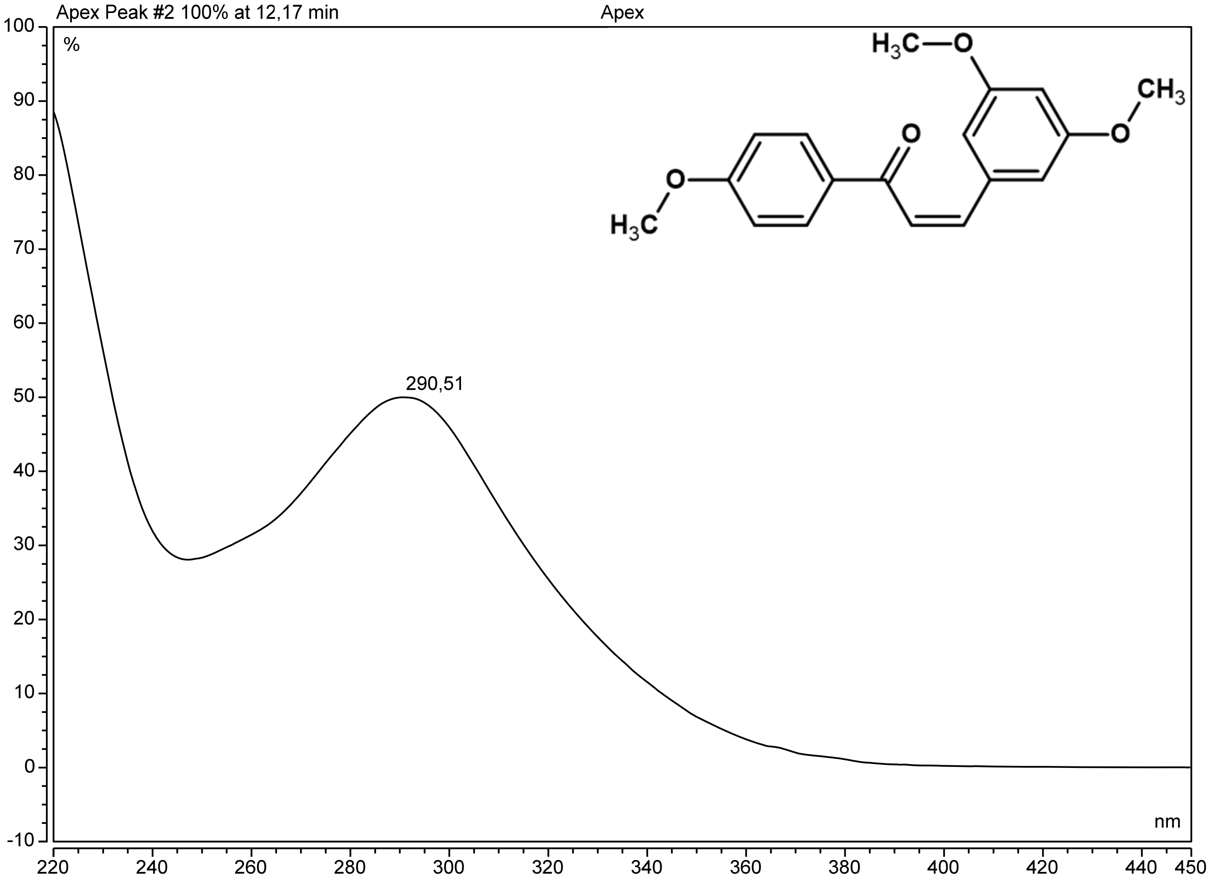

Figure S17 UV-Vis spectrum of *cis*-3,5,4′-trimethoxychalcone (**6b**).

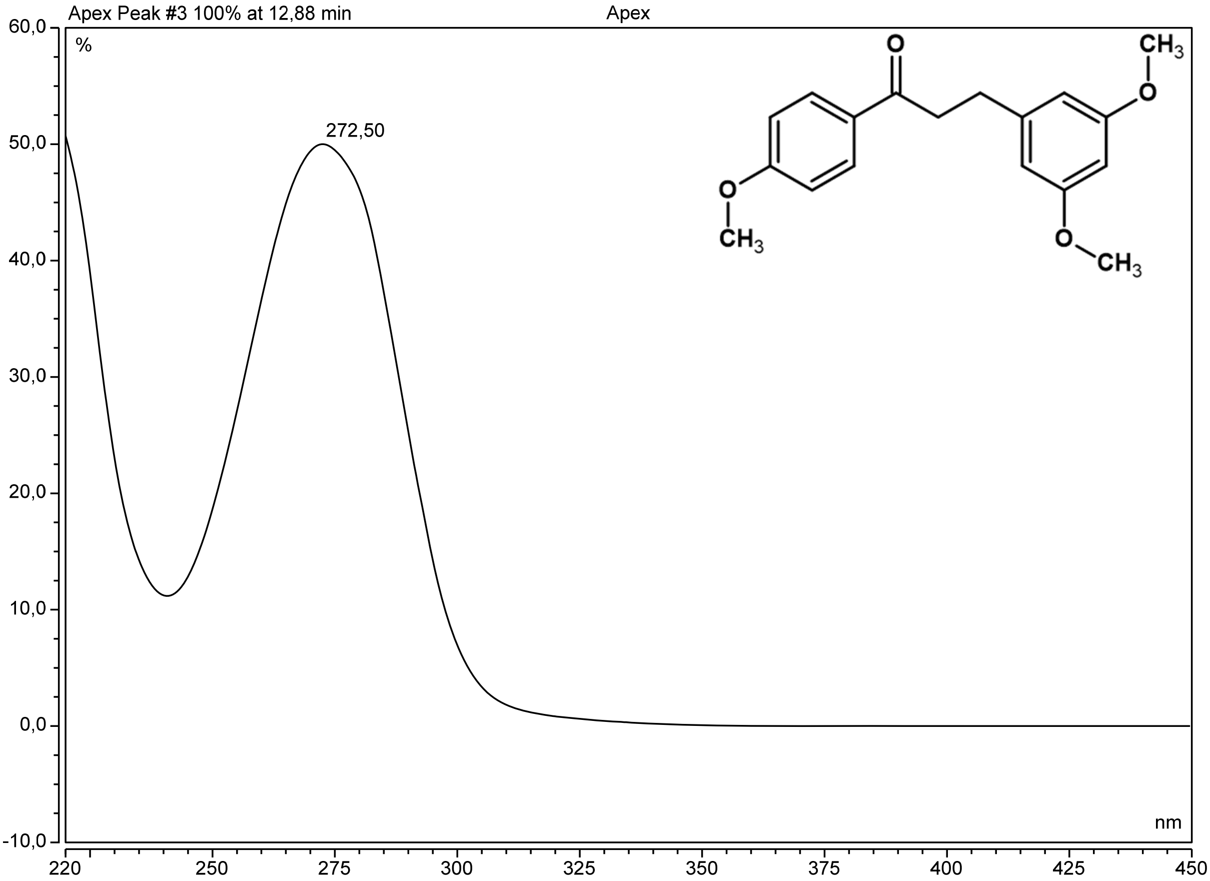

Figure S18 UV-Vis spectrum of 3,5,4′-trimethoxydihydrochalcone (**6c**).

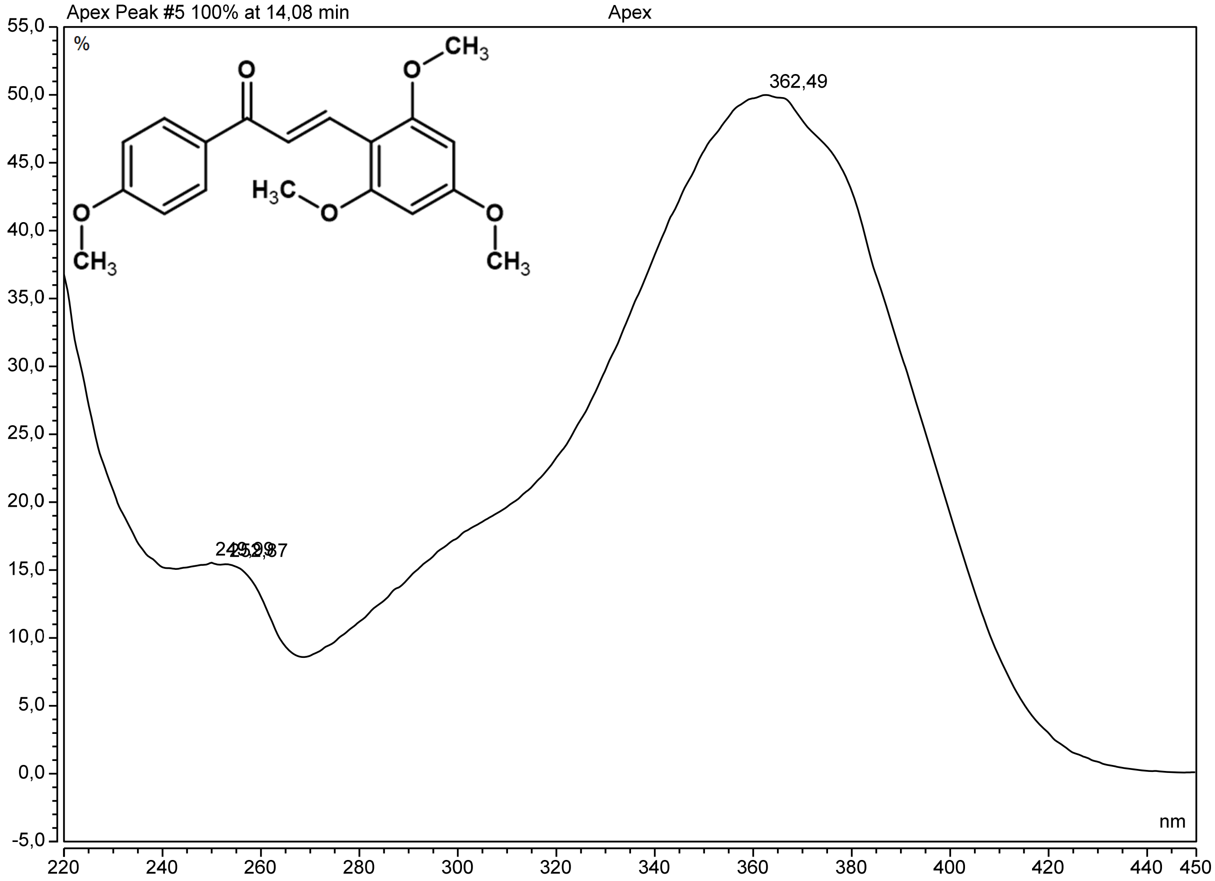

Figure S19 UV-Vis spectrum of *trans*-2,4,6,4′-tetramethoxychalcone (**7a**).

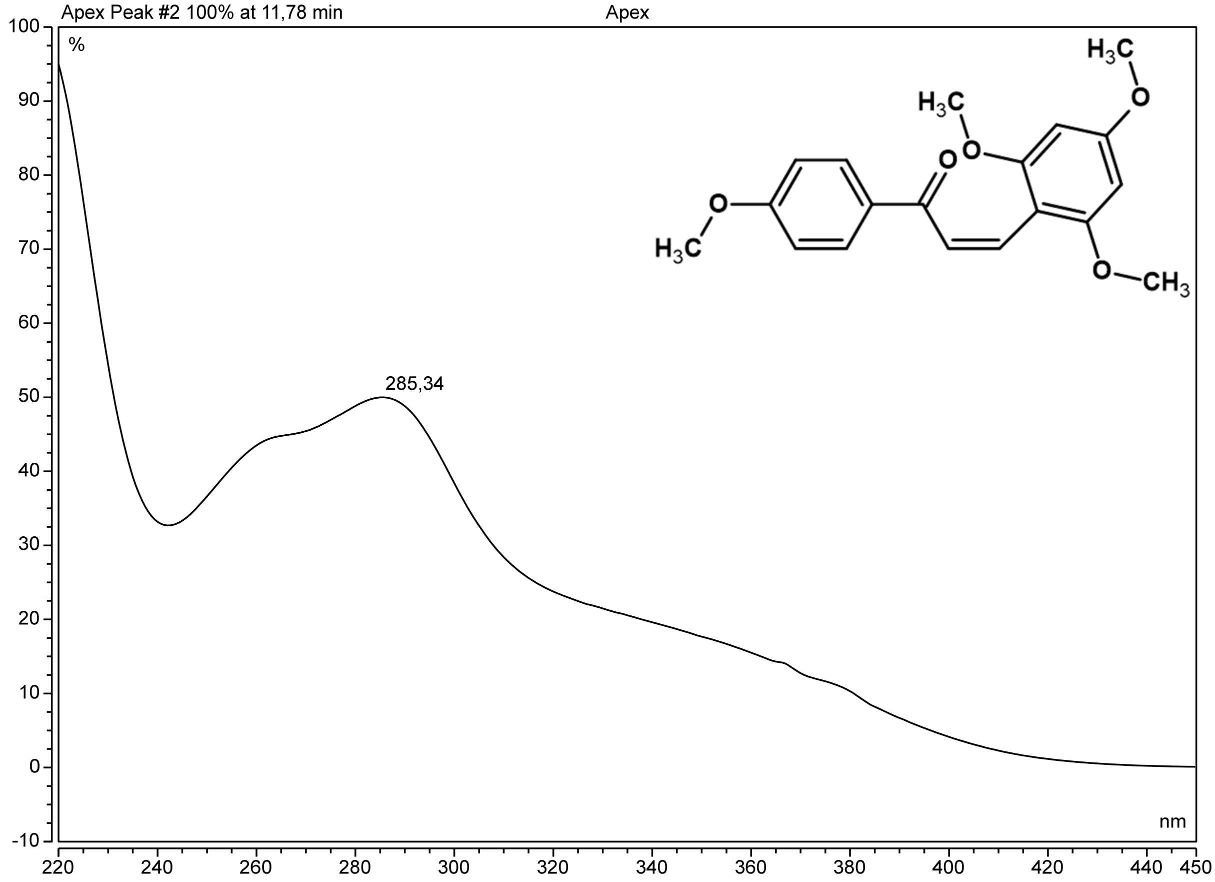

Figure S20 UV-Vis spectrum of *cis*-2,4,6,4′-tetramethoxychalcone (**7b**).

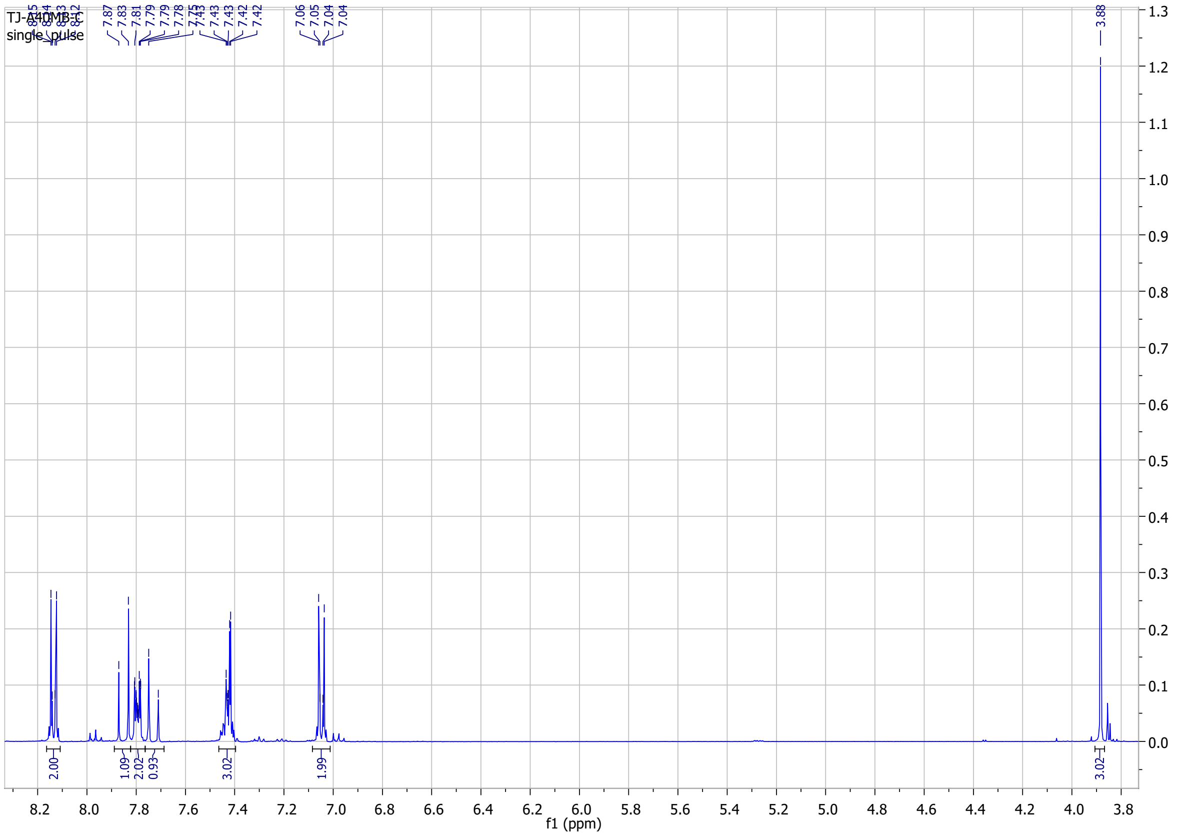

Figure S21 ^{1}H NMR spectrum of 4′-methoxychalcone (**1a**) (400 MHZ; acetone-d_6).

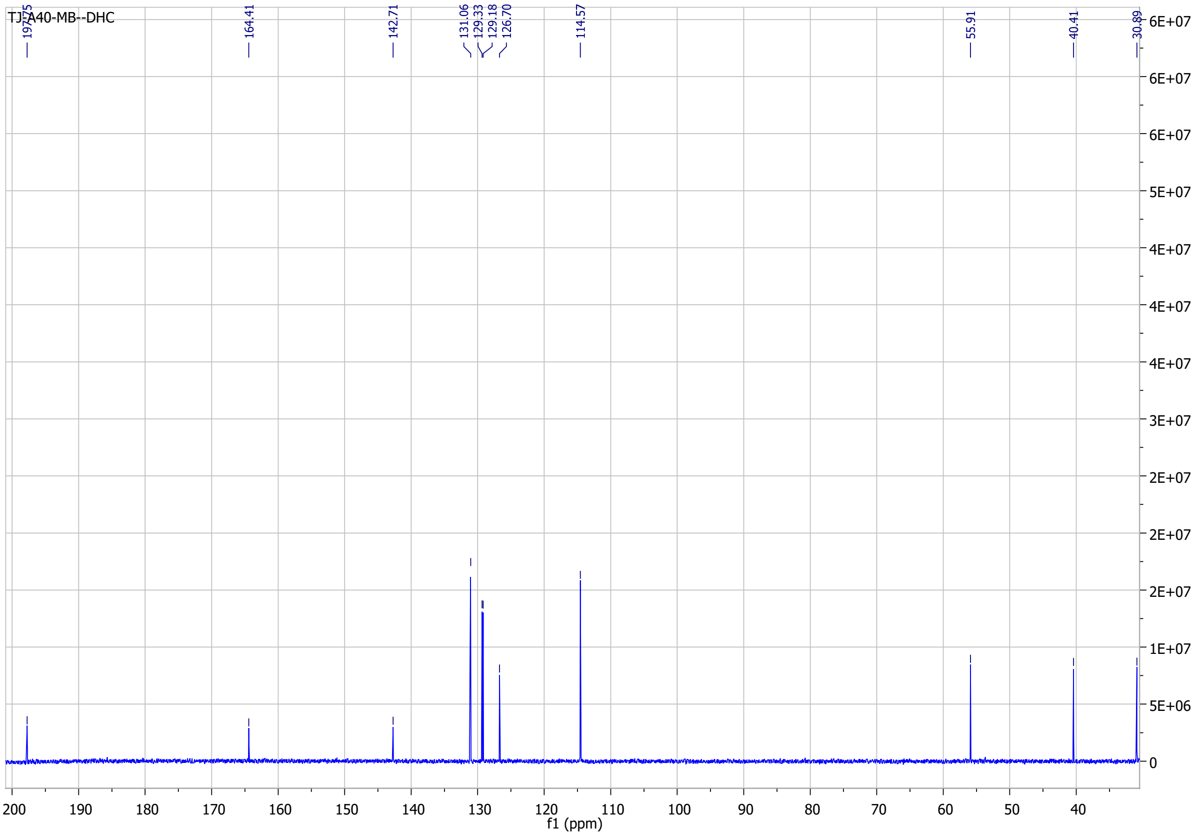

Figure S22 ^{13}C NMR spectrum of 4′-methoxychalcone (**1a**) (400 MHZ; acetone-d_6).

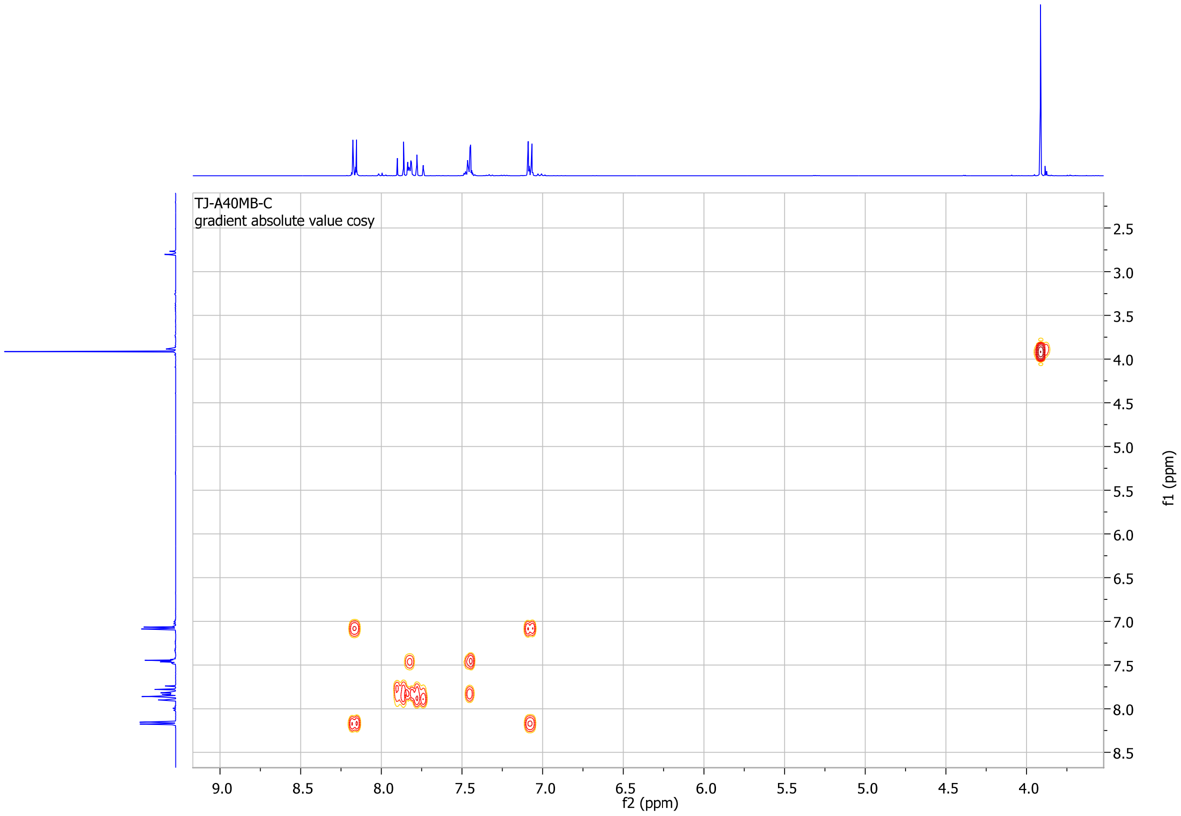

Figure S23 COSY NMR spectrum of 4′-methoxychalcone (**1a**) (400 MHZ; acetone-d6).

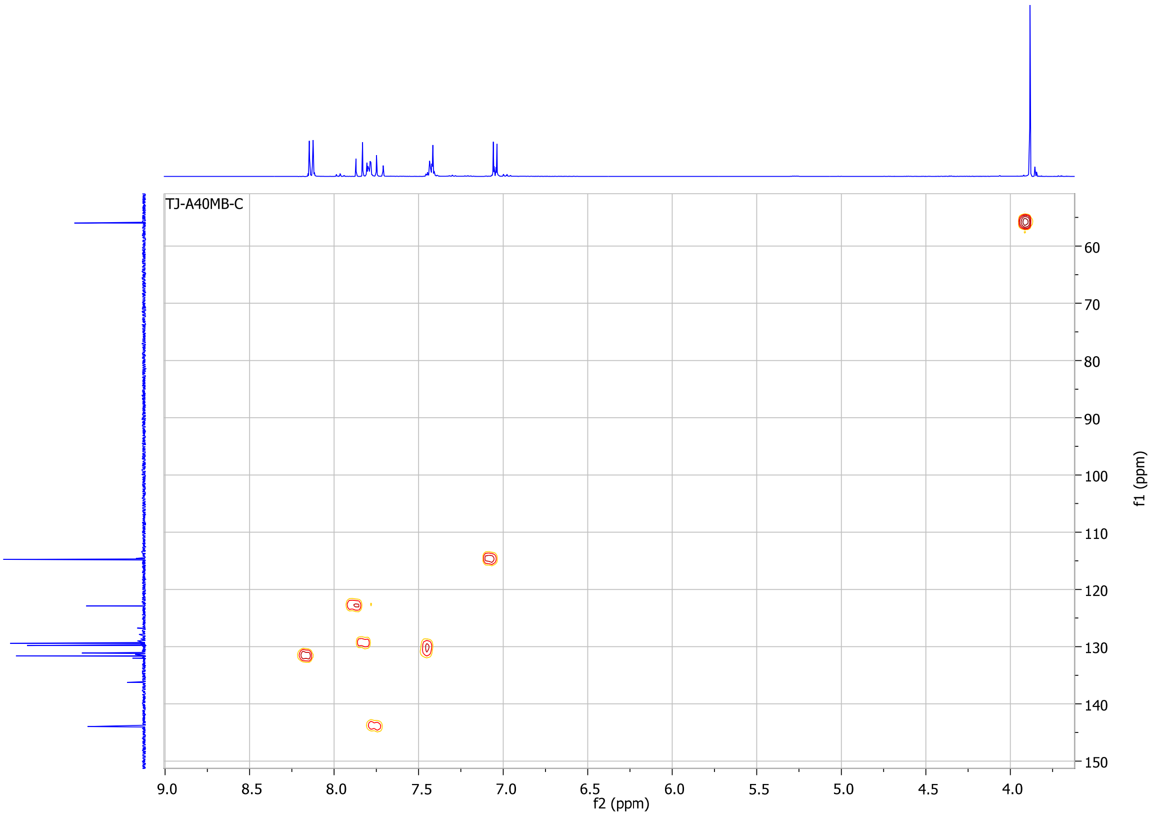

Figure S24 HSQC NMR spectrum of 4′-methoxychalcone (**1a**) (400 MHZ; acetone-d6).

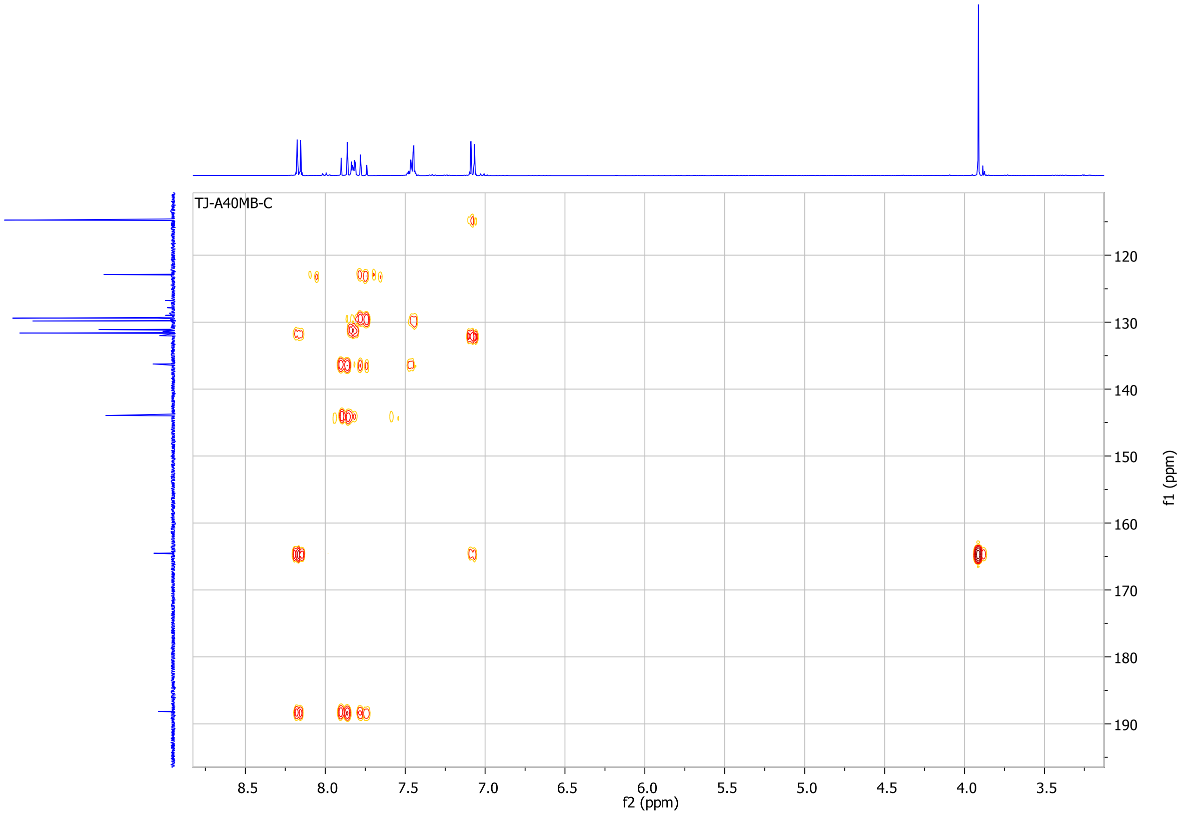

Figure S25 HMBC NMR spectrum of 4′-methoxychalcone (**1a**) (400 MHZ; acetone-d6).

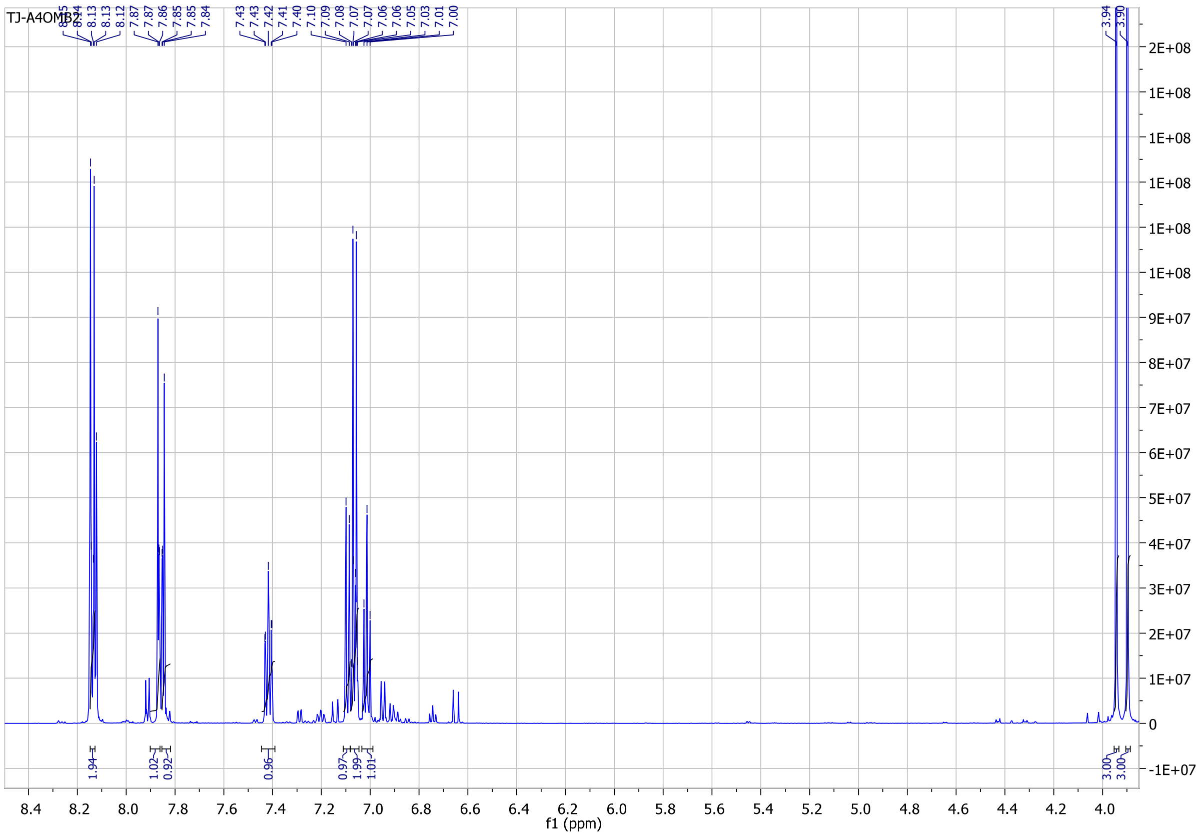

Figure S26 ^{1}H NMR spectrum of 2,4′-dimethoxychalcone (**2a**)(600 MHZ; acetone-d6).

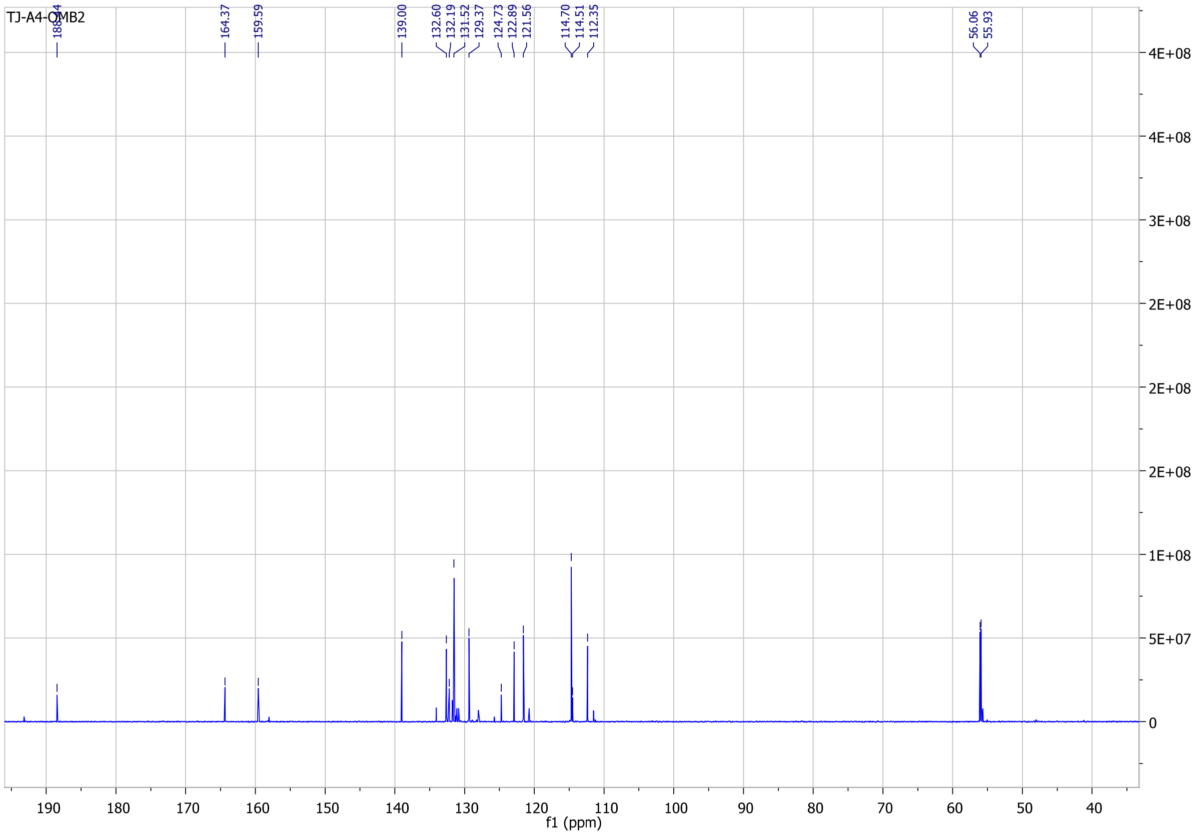

Figure S 27 ^{13}C NMR spectrum of 2,4′-dimethoxychalcone (**2a**) (600 MHZ; acetone-d$_6$).

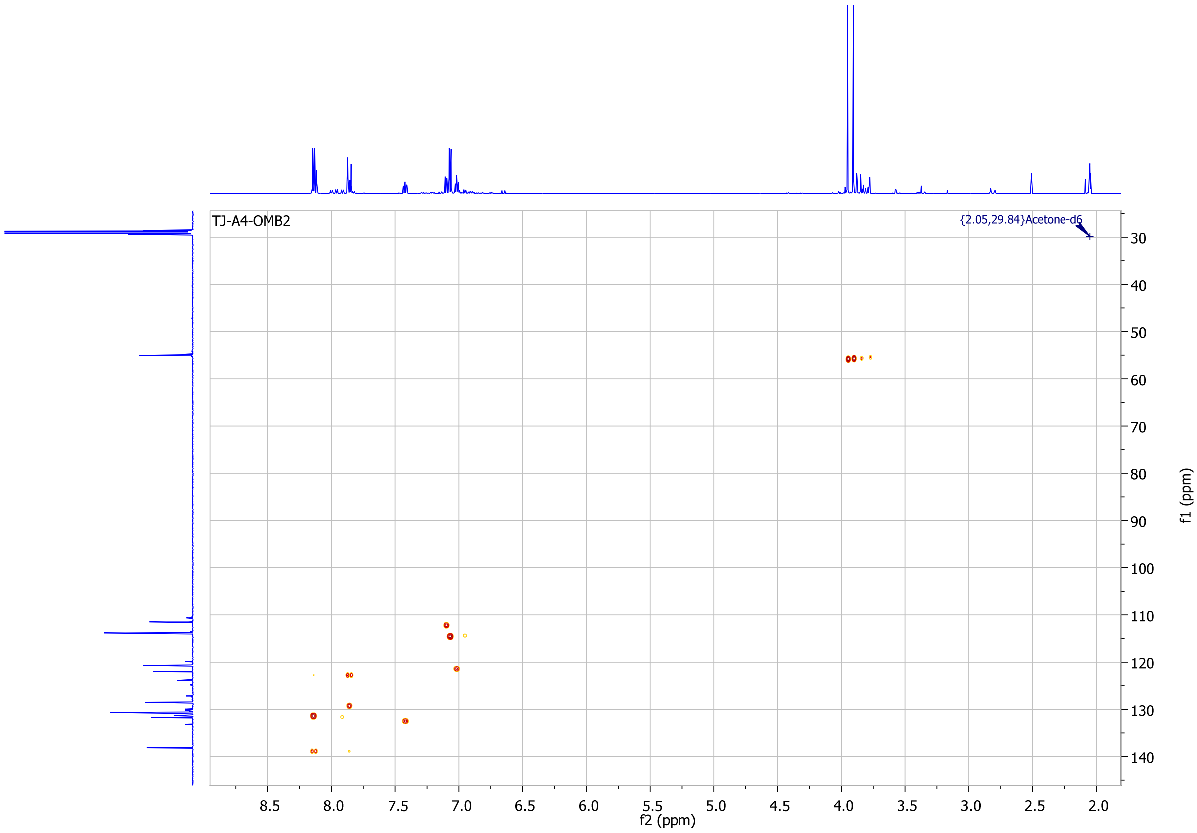

Figure S28 HSQC NMR spectrum of 2,4′-dimethoxychalcone (**2a**) (600 MHZ; acetone-d$_6$).

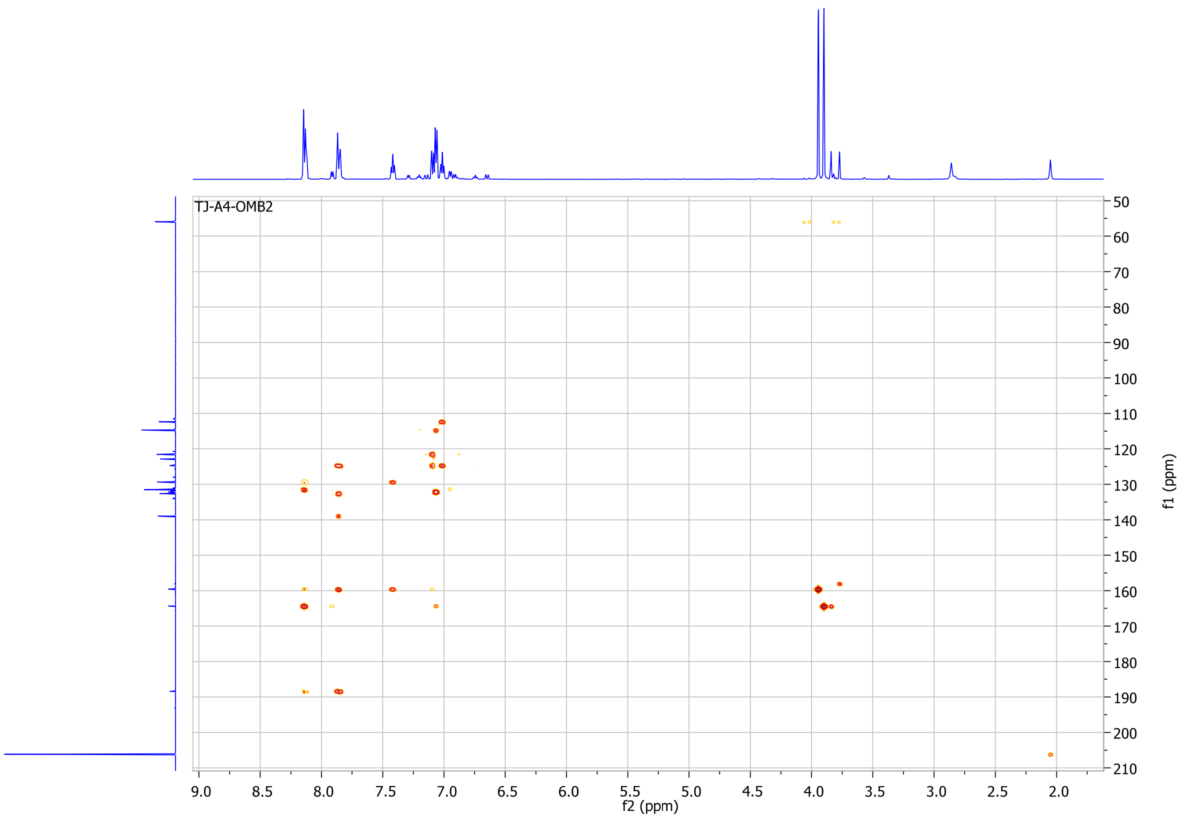

Figure S29 HMBC NMR spectrum of 2,4′-dimethoxychalcone (**2a**) (600 MHZ; acetone-d6).

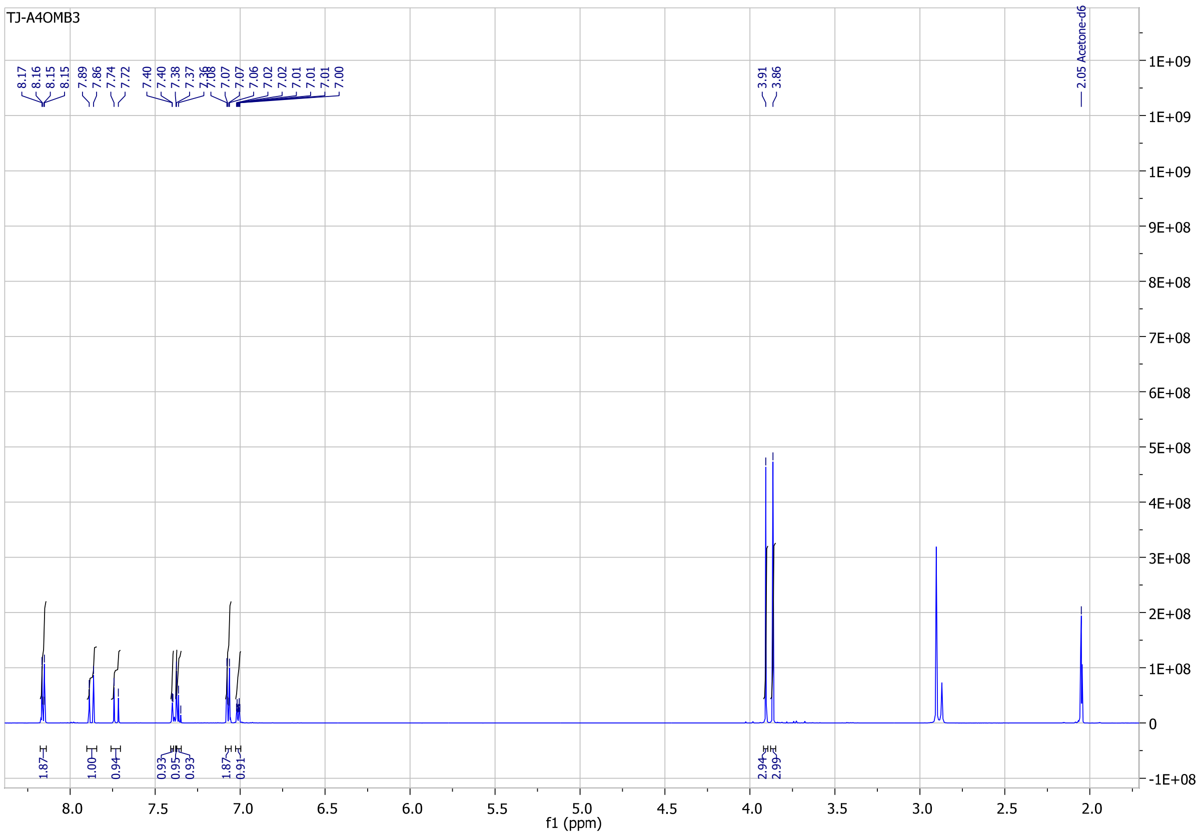

Figure S30 ^{1}H NMR spectrum of 3,4'-dimethoxychalcone (**3a**) (600 MHz; acetone-d6).

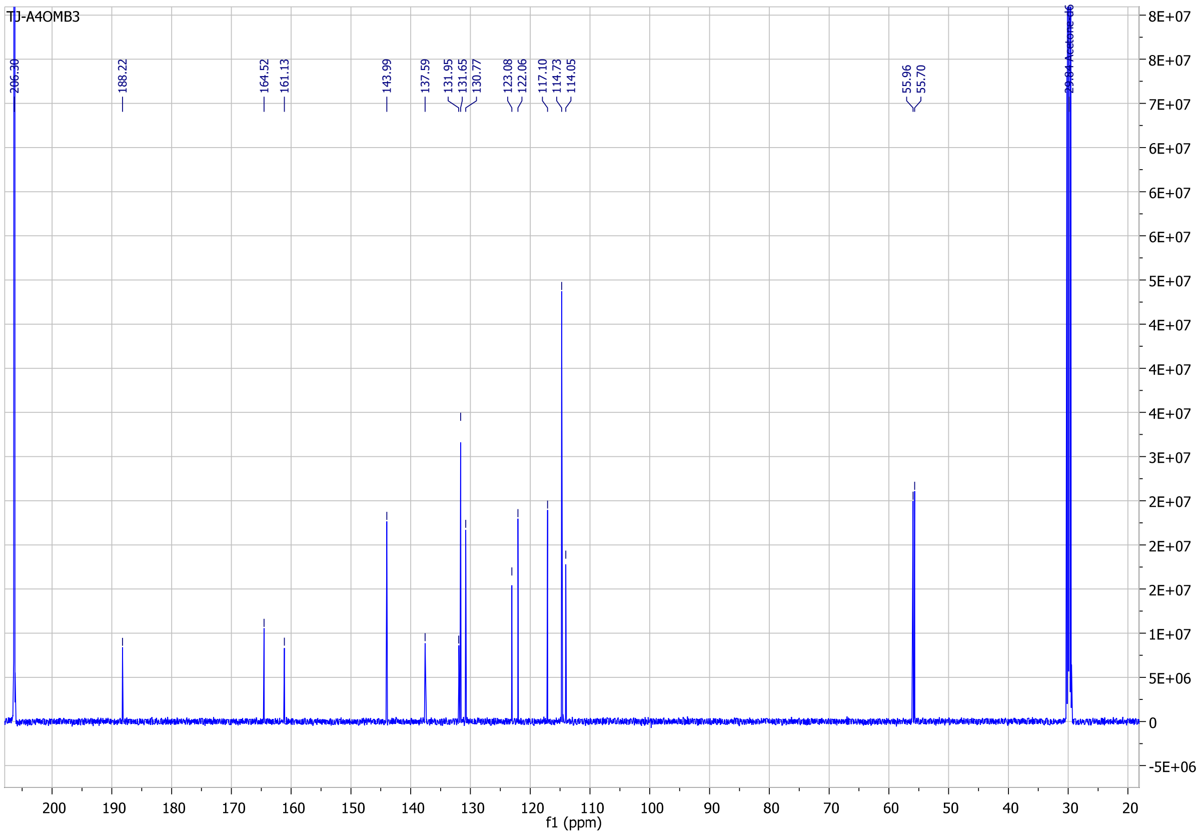

Figure S31 ^{13}C NMR spectrum of 3,4'-dimethoxychalcone (**3a**) (600 MHz; acetone-d$_6$).

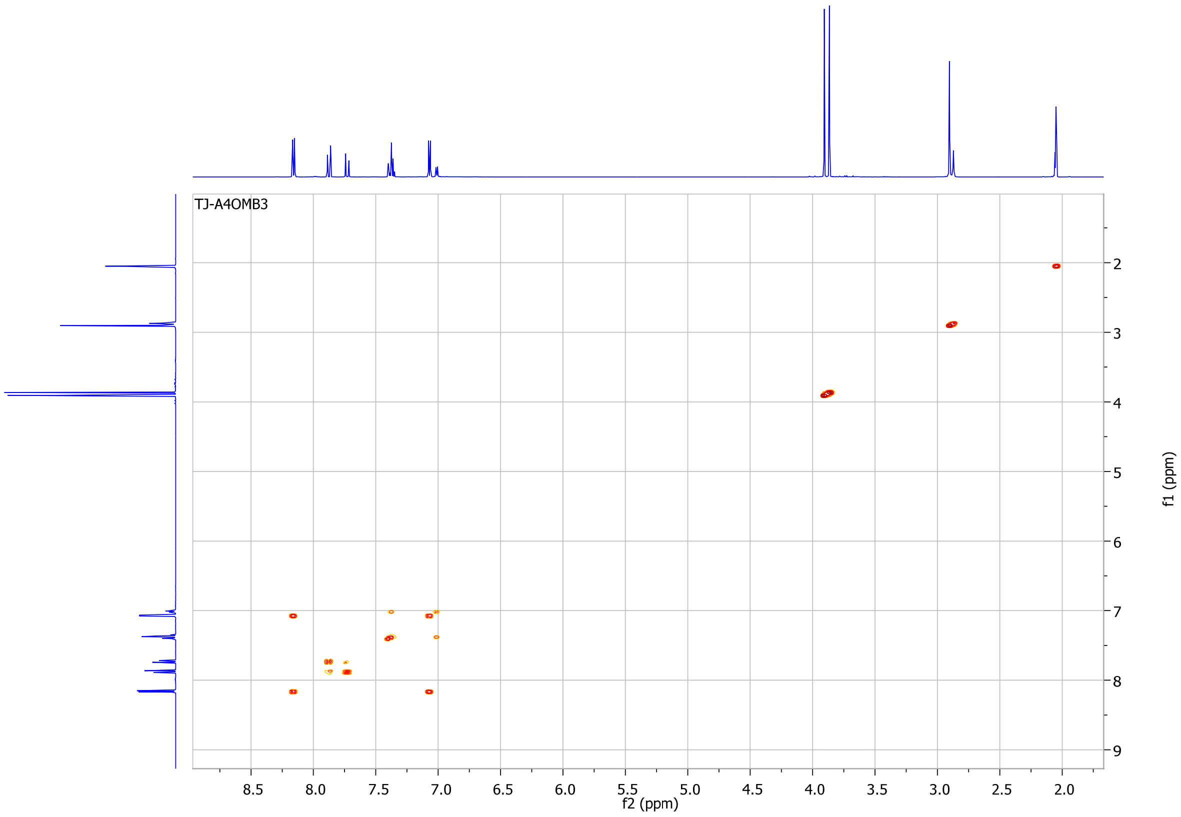

Figure S32 COSY NMR spectrum of 3,4'-dimethoxychalcone (**3a**) (600 MHz; acetone-d$_6$).

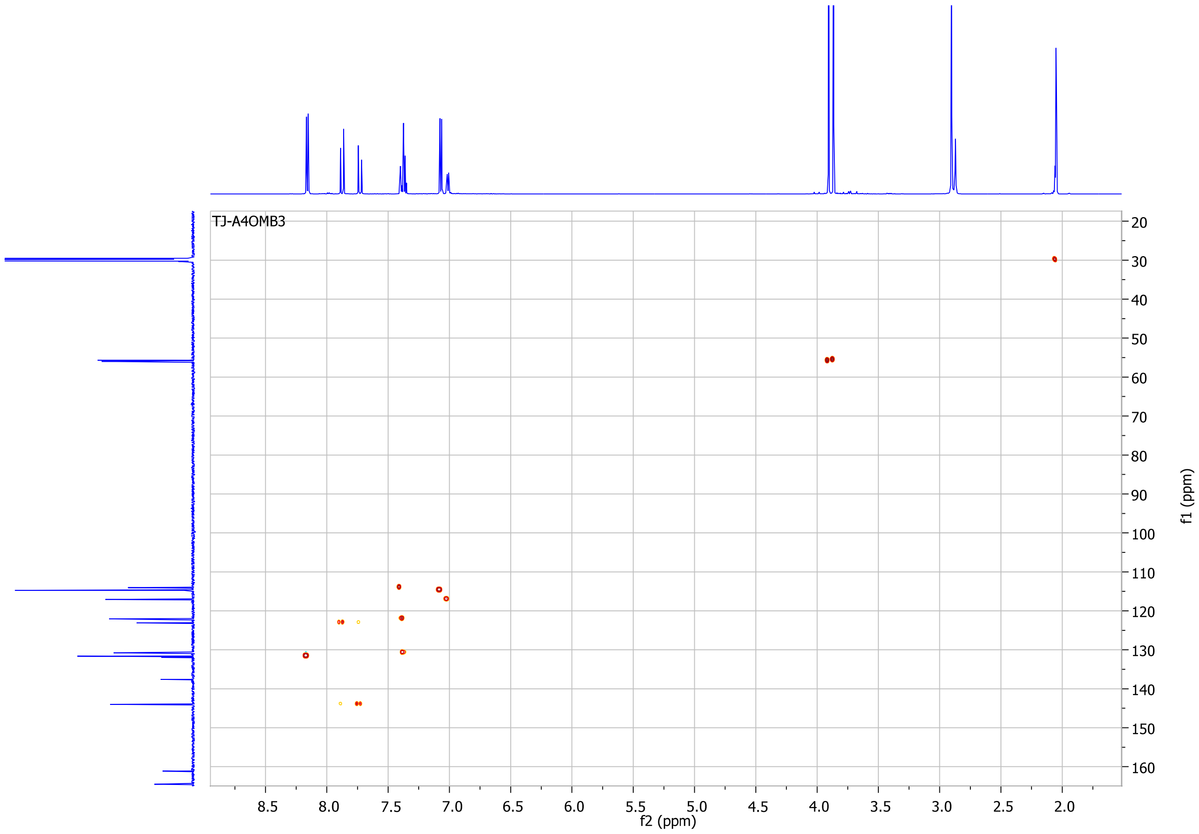

Figure S33 HSQC NMR spectrum of 3,4'-dimethoxychalcone (**3a**) (600 MHz; acetone-d6).

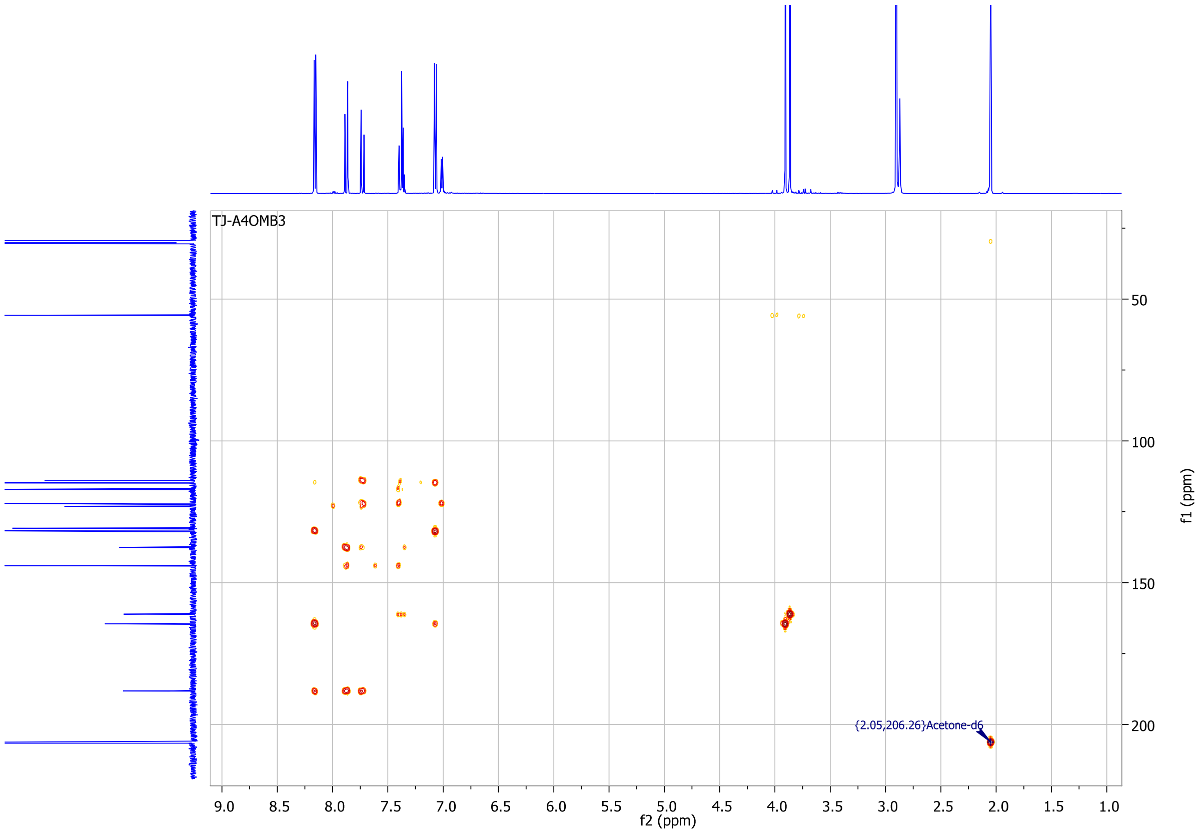

Figure S34 HMBC NMR spectrum of 3,4'-dimethoxychalcone (600 MHz; acetone-d6).

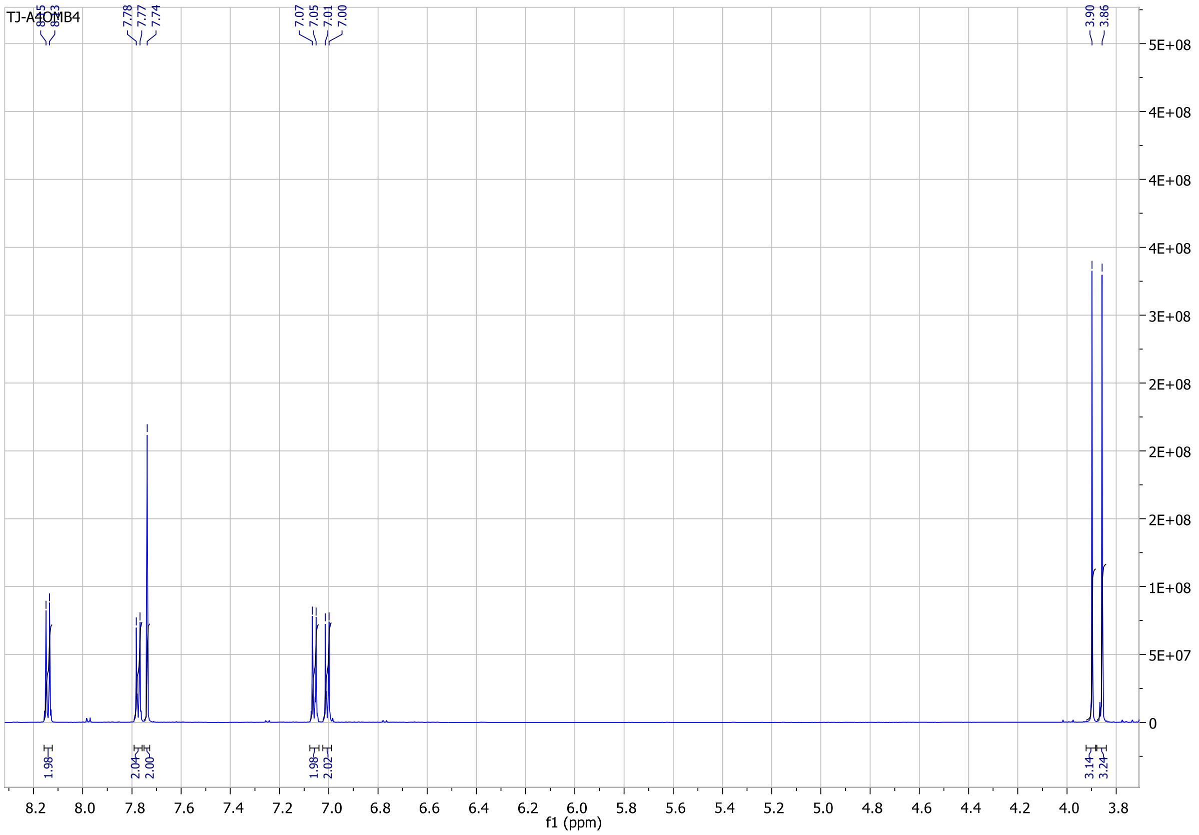

Figure S35 ^{1}H NMR spectrum of 4,4'-dimethoxychalcone (**4a**) (600 MHz; acetone-d$_6$).

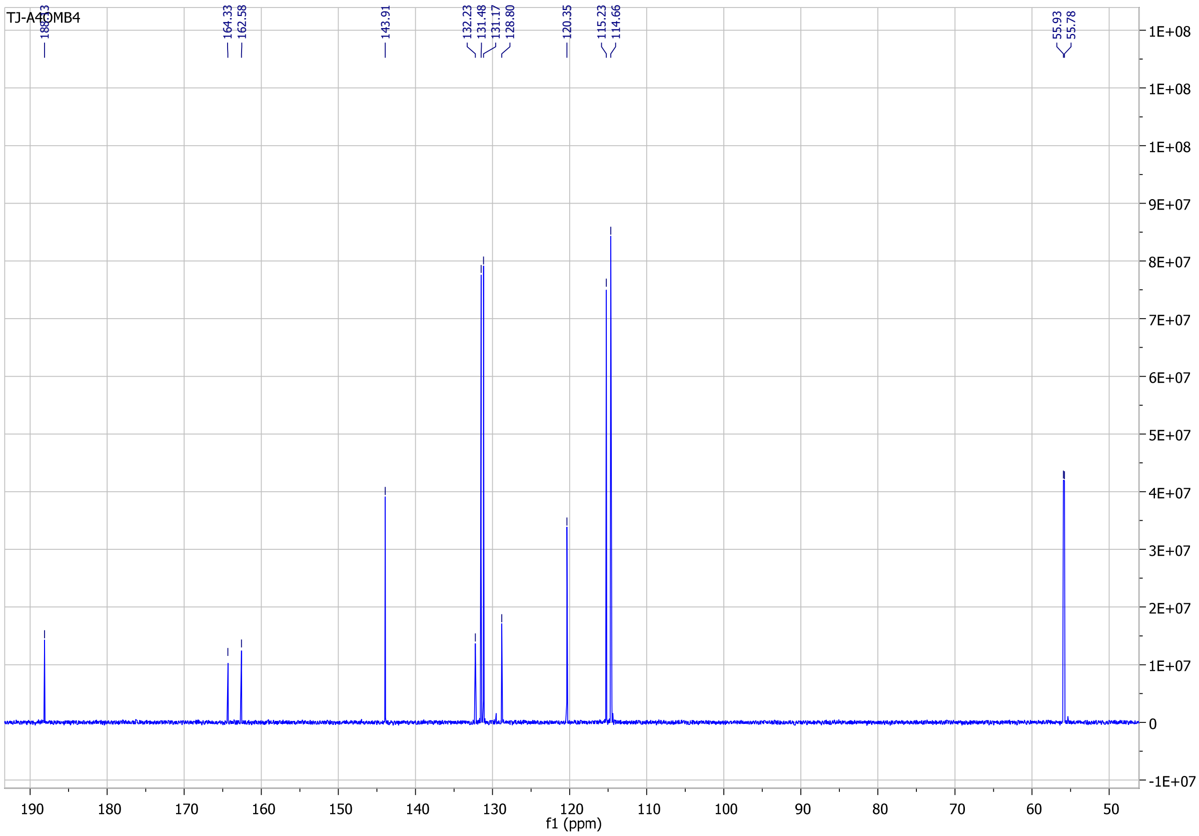

Figure S36 ^{13}C NMR spectrum of 4,4'-dimethoxychalcone (**4a**) (600 MHz; acetone-d$_6$).

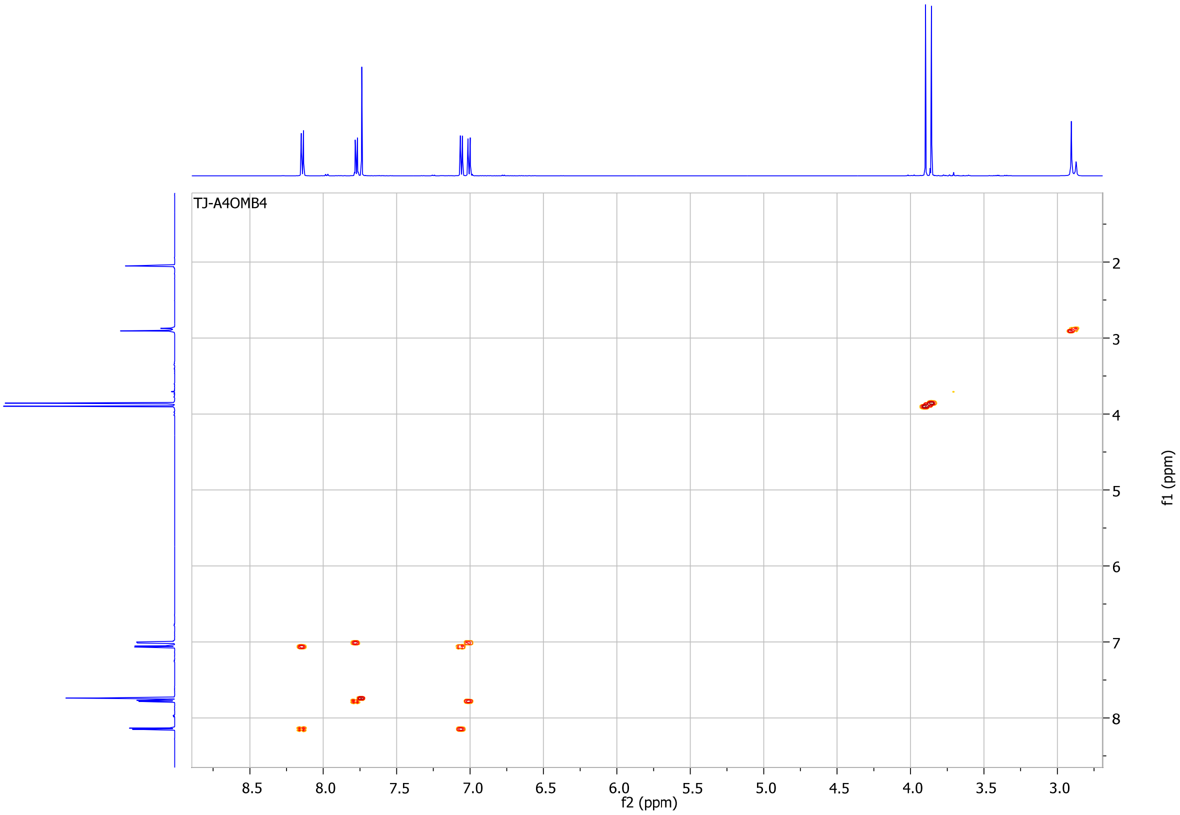

Figure S37 COSY NMR spectrum of 4,4'-dimethoxychalcone (**4a**) (600 MHz; acetone-d_6).

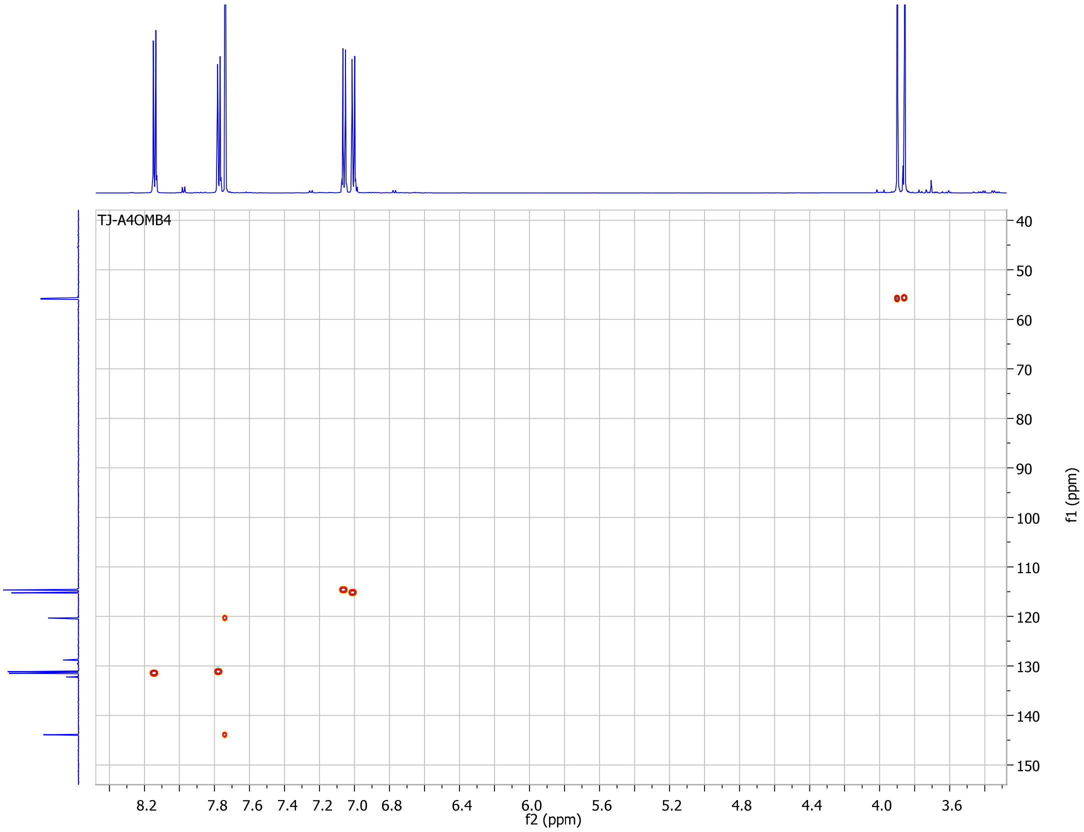

Figure S38 HSQC NMR spectrum of 4,4'-dimethoxychalcone (600 MHz; acetone-d6).

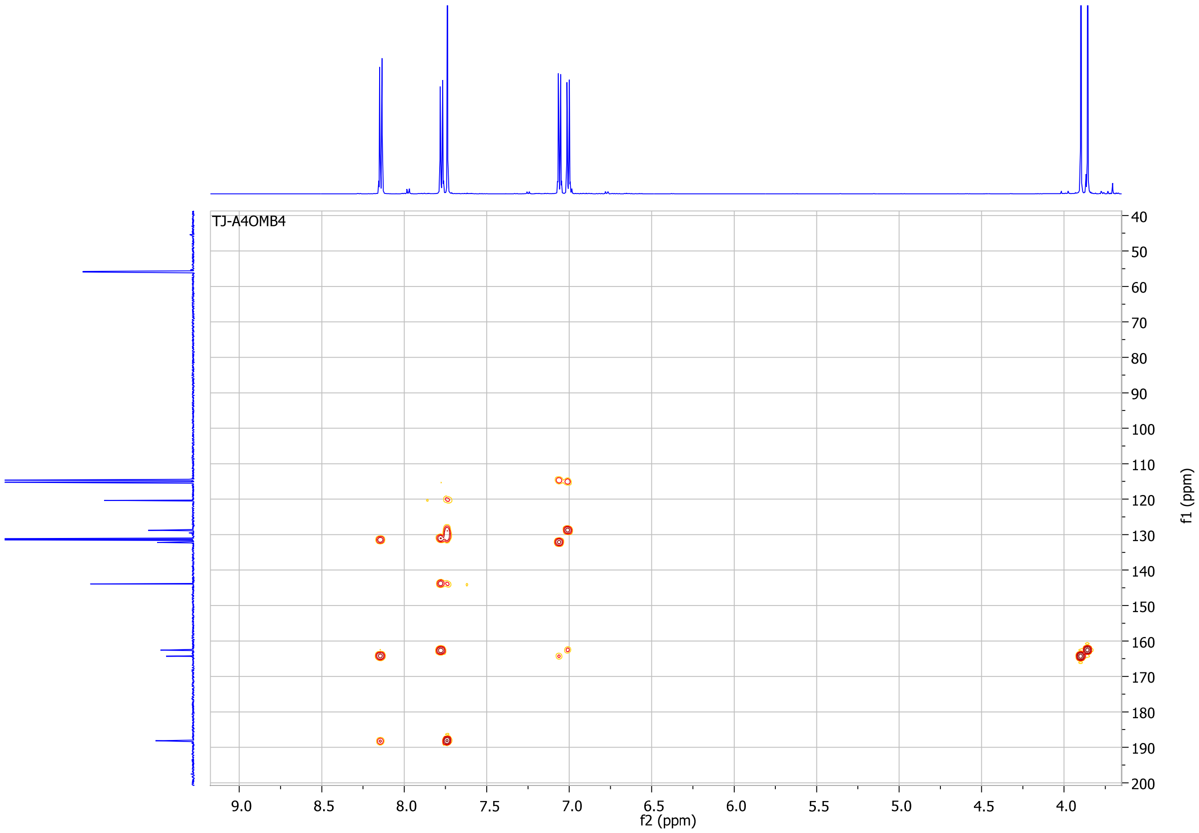

Figure S39 HMBC NMR spectrum of 4,4'-dimethoxychalcone (**4a**) (600 MHz; acetone-d_6).

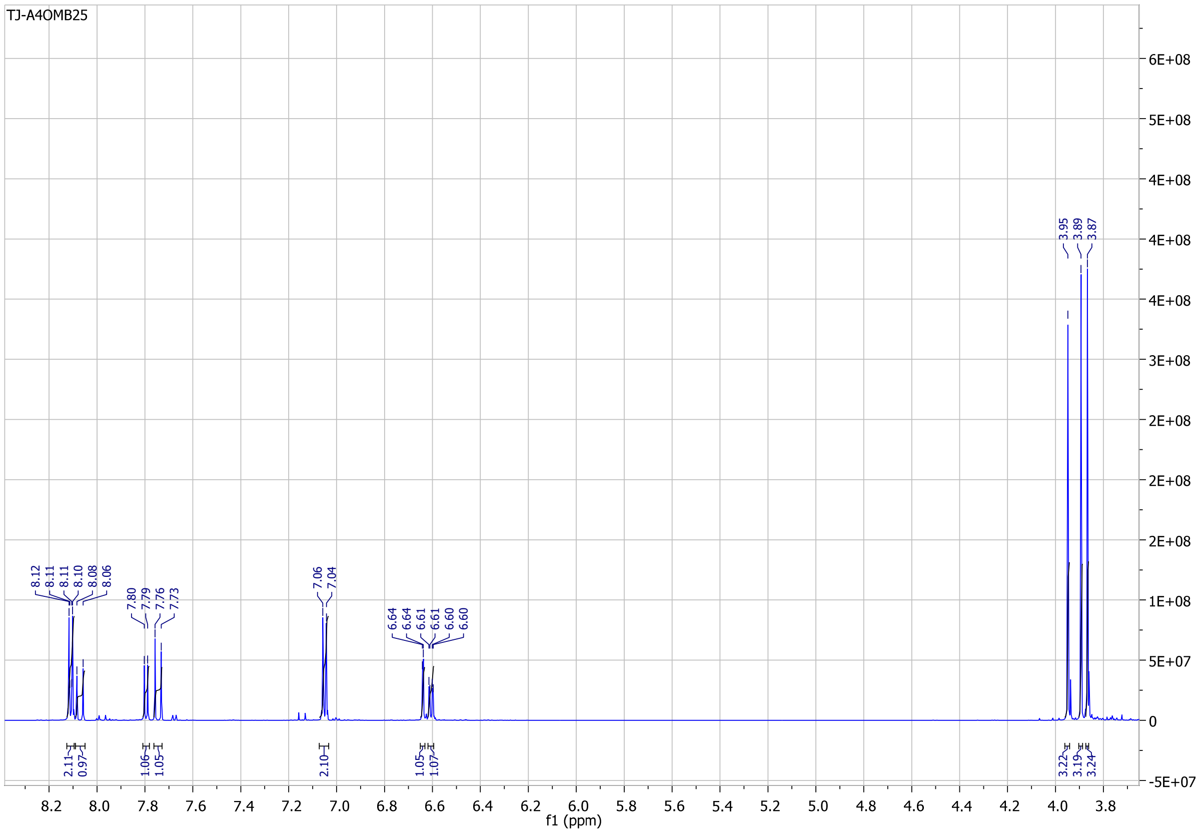

Figure S40 ^{1}H NMR spectrum of 2,5,4'-trimethoxychalcone (**5a**) (600 MHz; acetone-d_6).

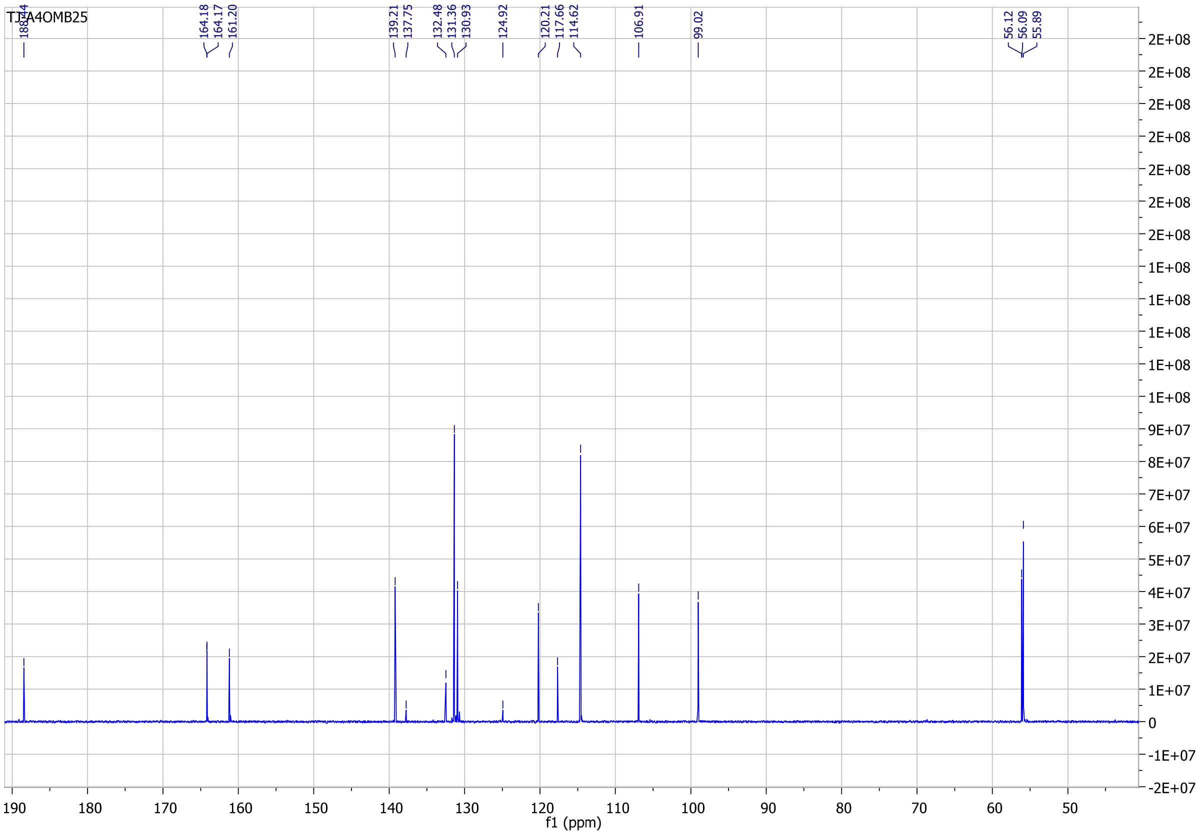

Figure S41 ^{13}C NMR spectrum of 2,5,4'-trimethoxychalcone (**5a**) (600 MHz; acetone-d$_6$).

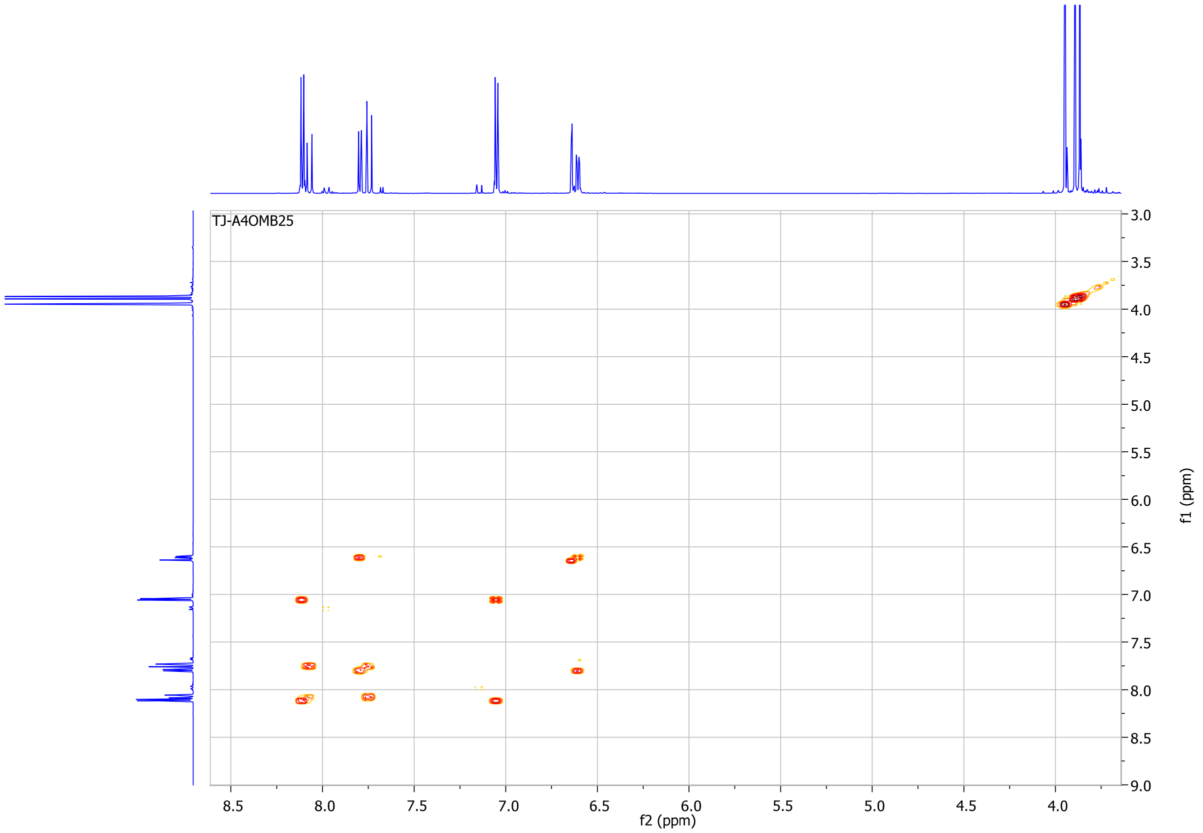

Figure S42 COSY NMR spectrum of 2,5,4'-trimethoxychalcone (**5a**) (600 MHz; acetone-d$_6$).

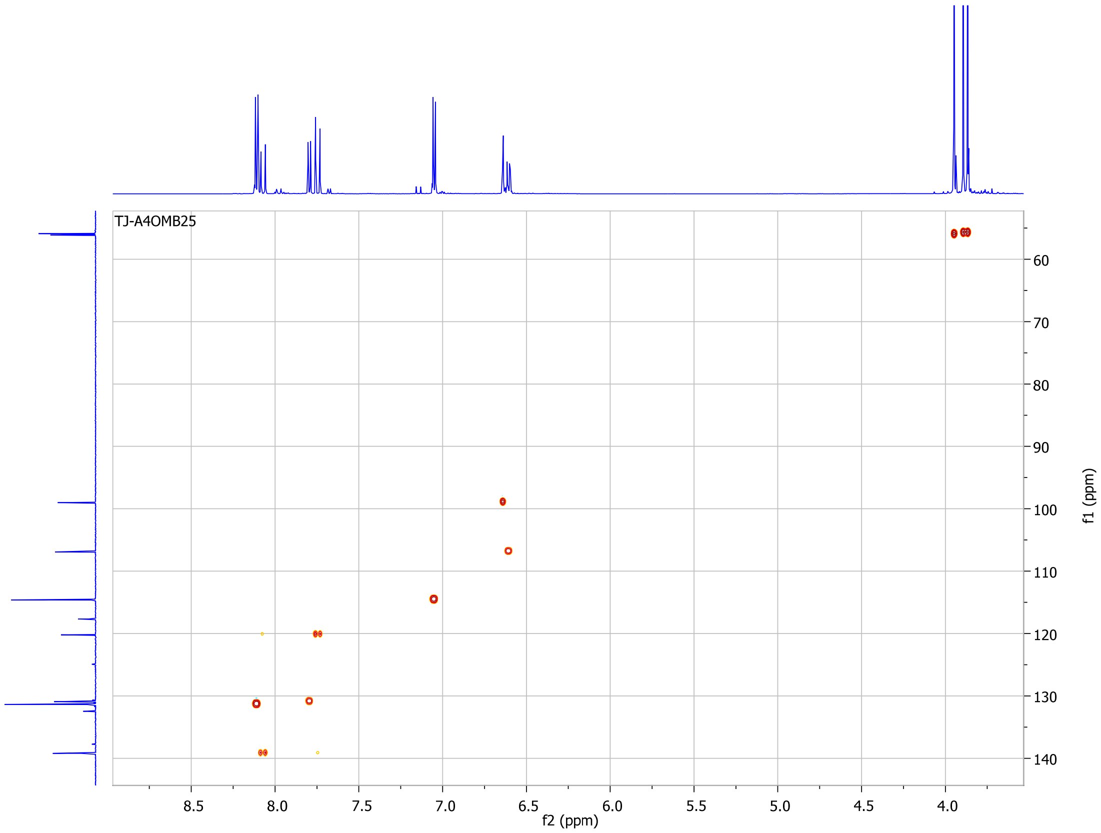

Figure S43 HSQC NMR spectrum of 2,5,4'-trimethoxychalcone (**5a**) (600 MHz; acetone-d_6).

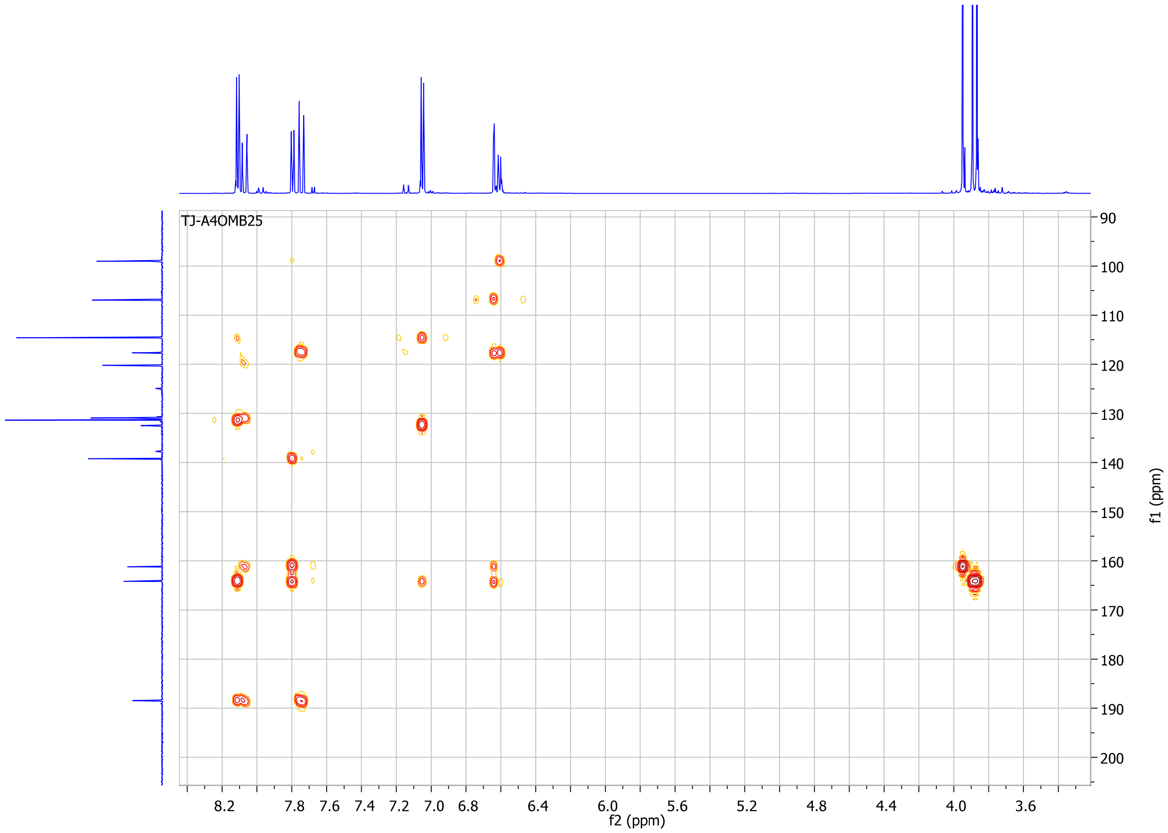

Figure S44 HMBC NMR spectrum of 2,5,4'-trimethoxychalcone (**5a**) (600 MHz; acetone-d_6).

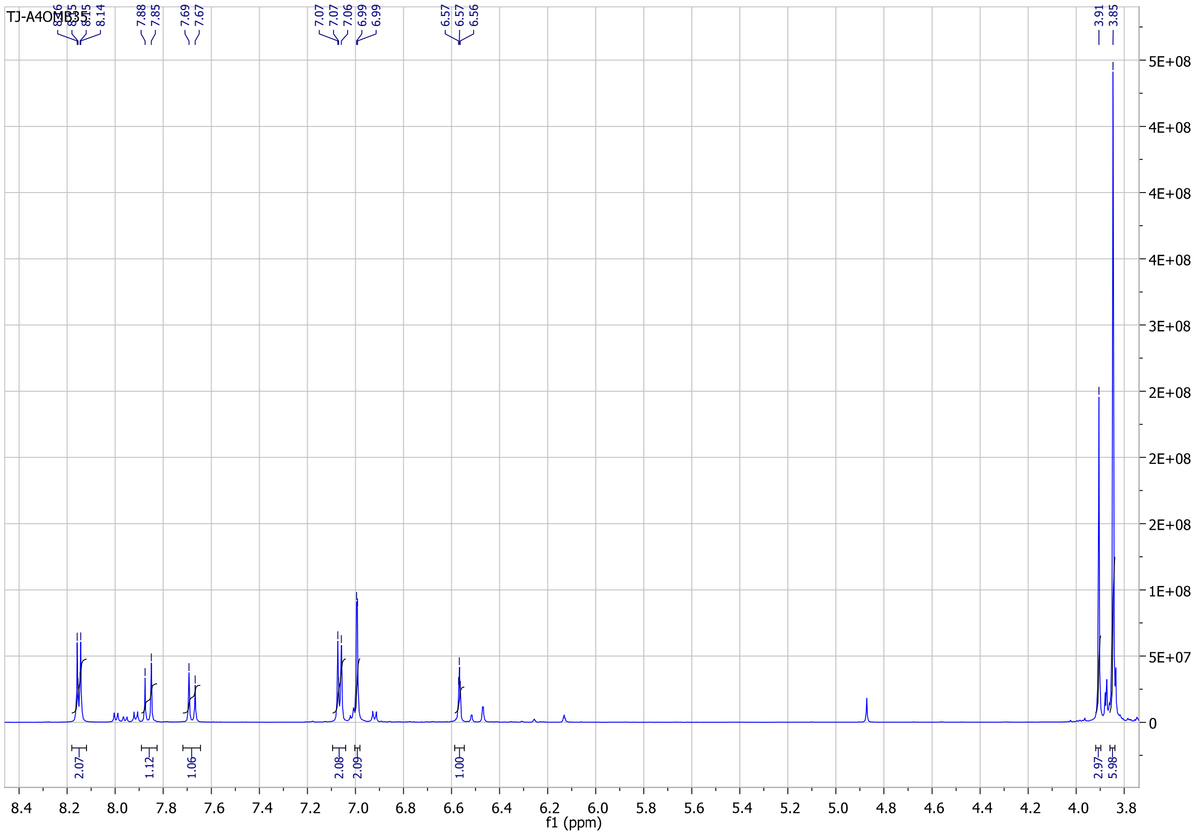

Figure S45 ¹H NMR spectrum of 3,5,4'-trimethoxychalcone (**6a**) (600 MHz; acetone-d₆).

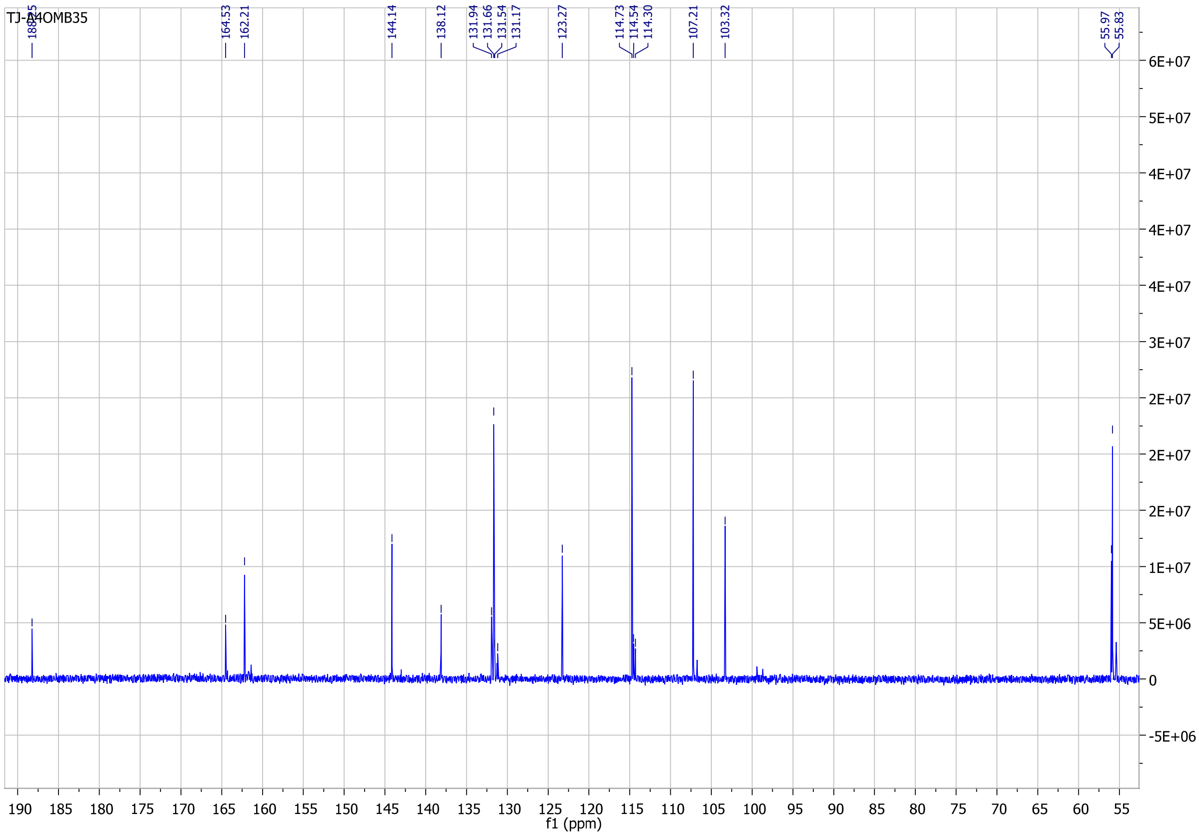

Figure S46 ¹³C NMR spectrum of 3,5,4'-trimethoxychalcone (**6a**) (600 MHz; acetone-d₆).

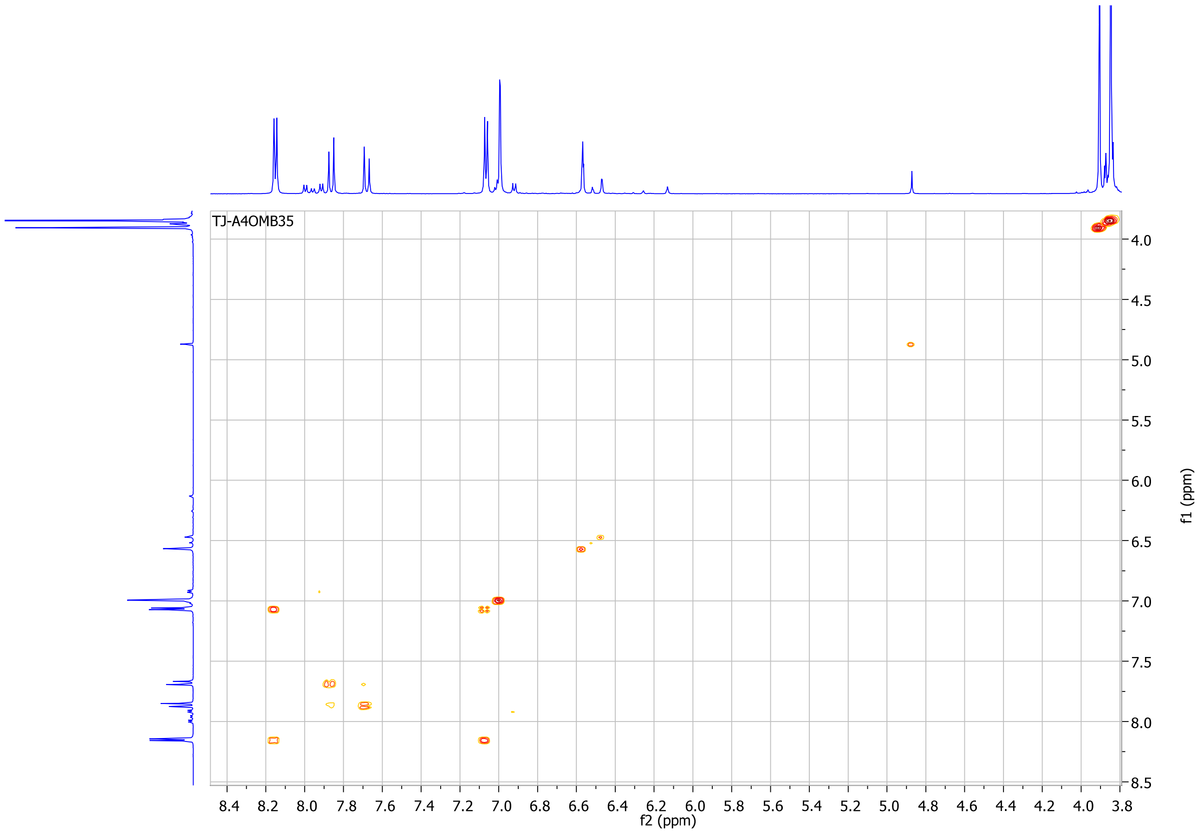

Figure S47 COSY NMR spectrum of 3,5,4'-trimethoxychalcone (**6a**) (600 MHz; acetone-d$_6$).

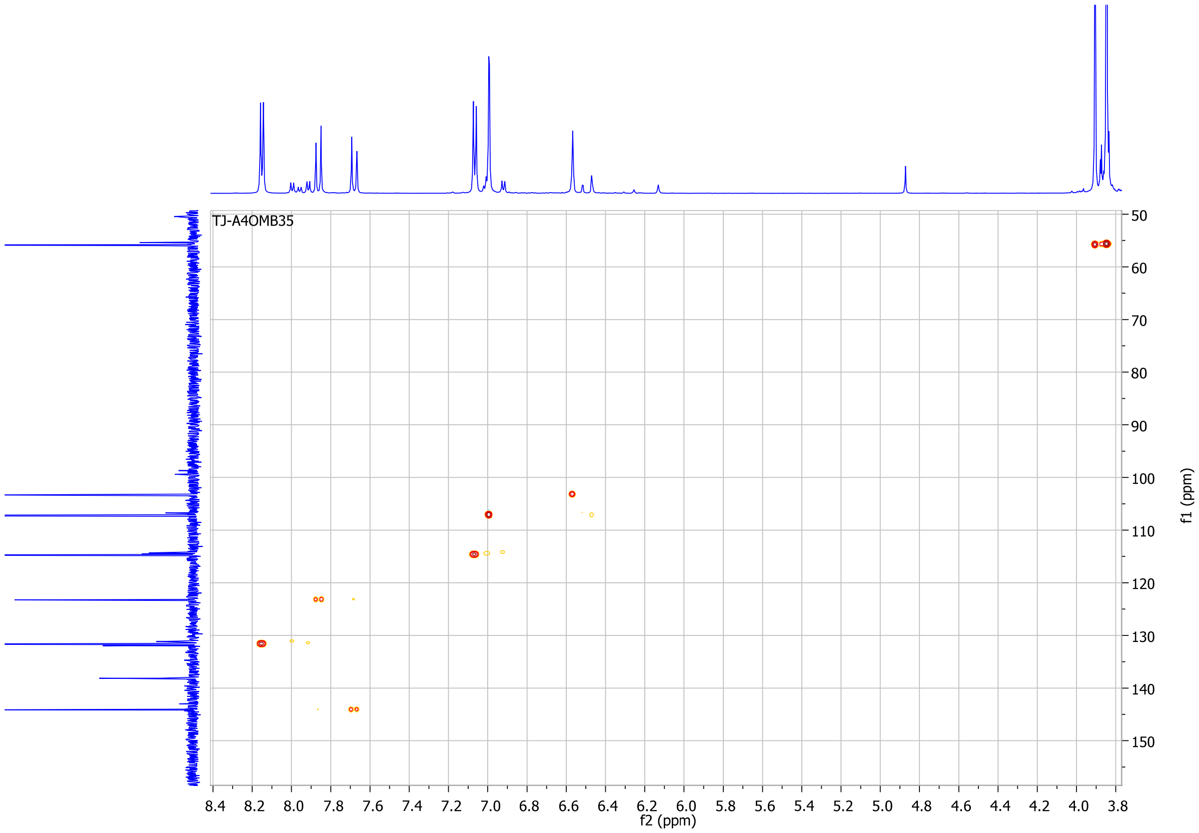

Figure S48 HSQC NMR spectrum of 3,5,4'-trimethoxychalcone (**6a**) (600 MHz; acetone-d$_6$).

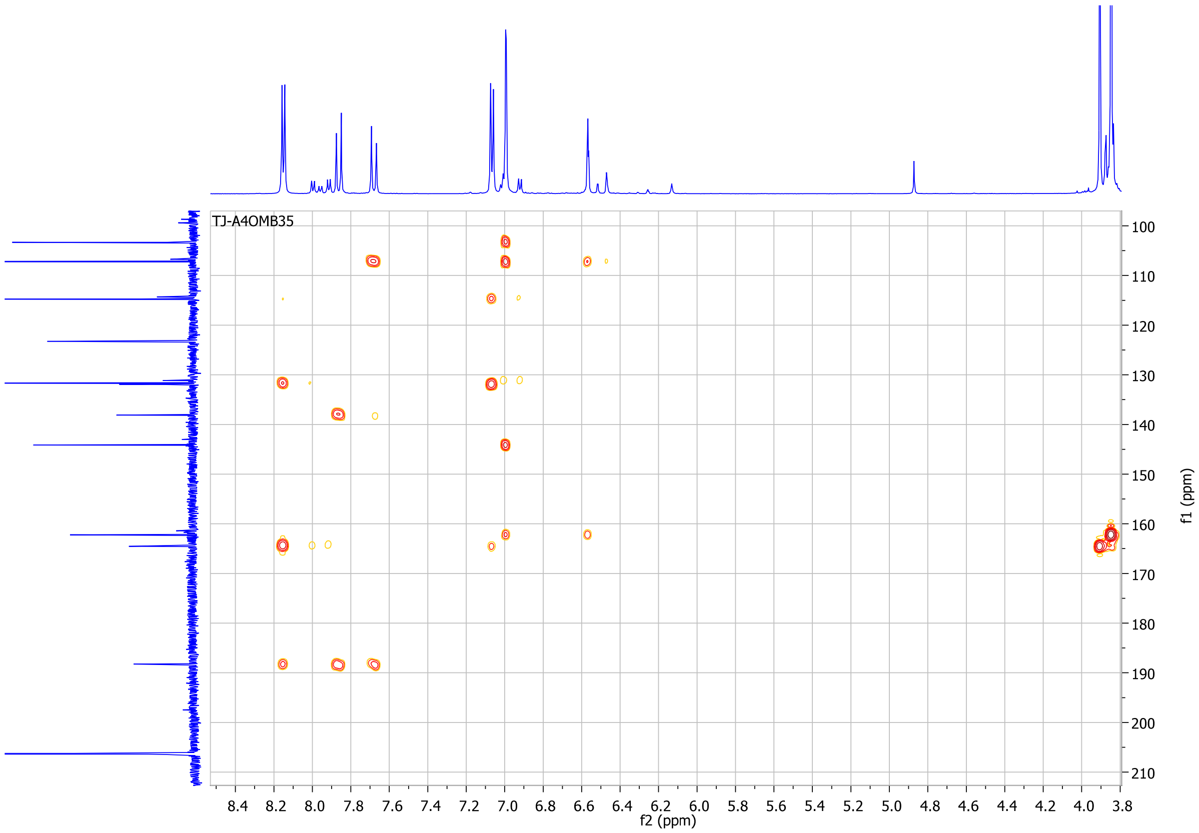

Figure S49 HMBC NMR spectrum of 3,5,4'-trimethoxychalcone (**6a**) (600 MHz; acetone-d6).

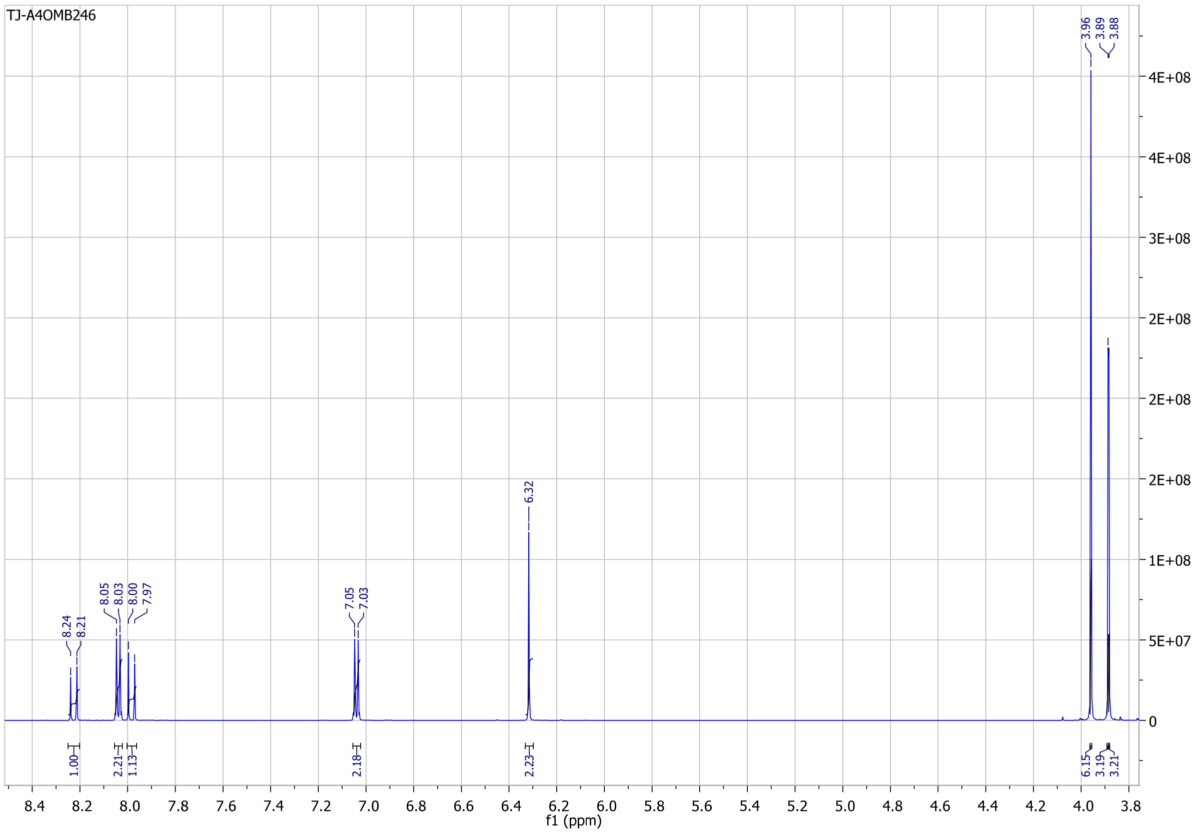

Figure S50 ^{1}H NMR spectrum of 2,4,6,4'-tetramethoxychalcone (**7a**) (600 MHz; acetone-d6).

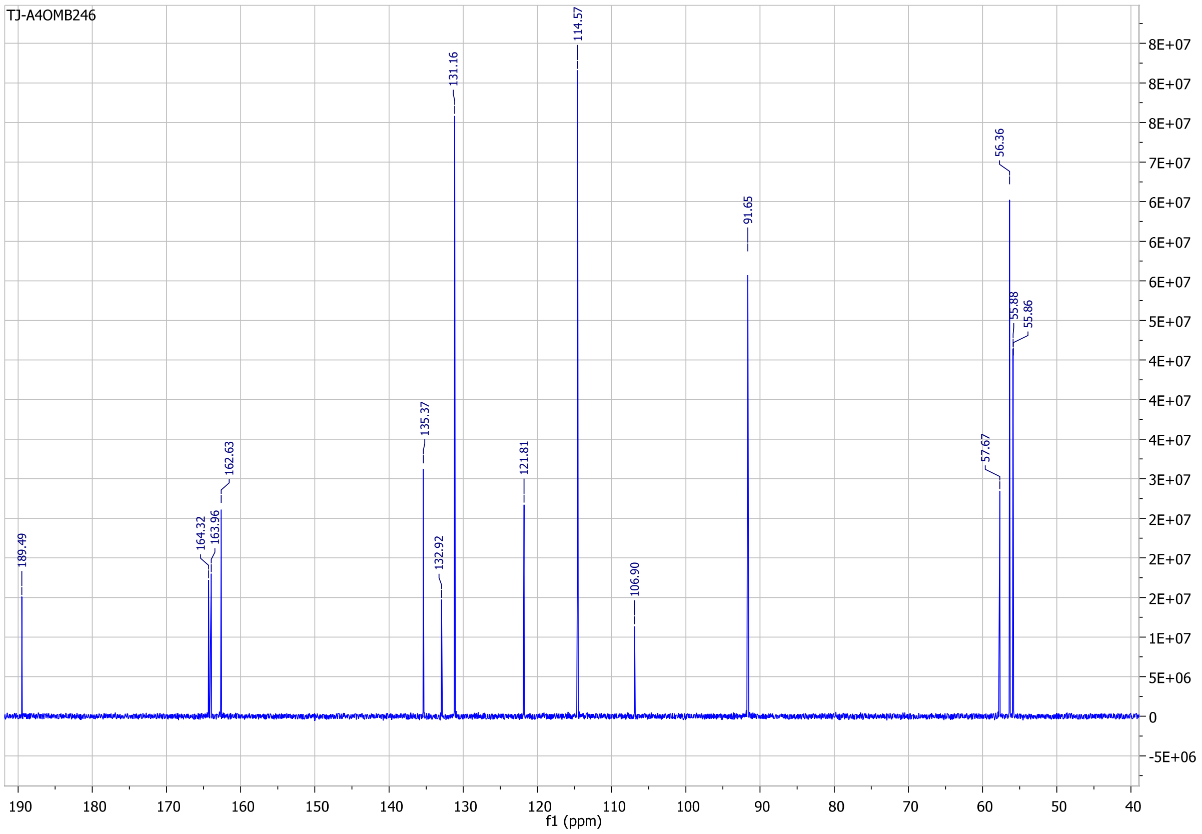

Figure S51 ^{13}C NMR spectrum of 2,4,6,4'-tetramethoxychalcone (**7a**) (600 MHz; acetone-d$_6$).

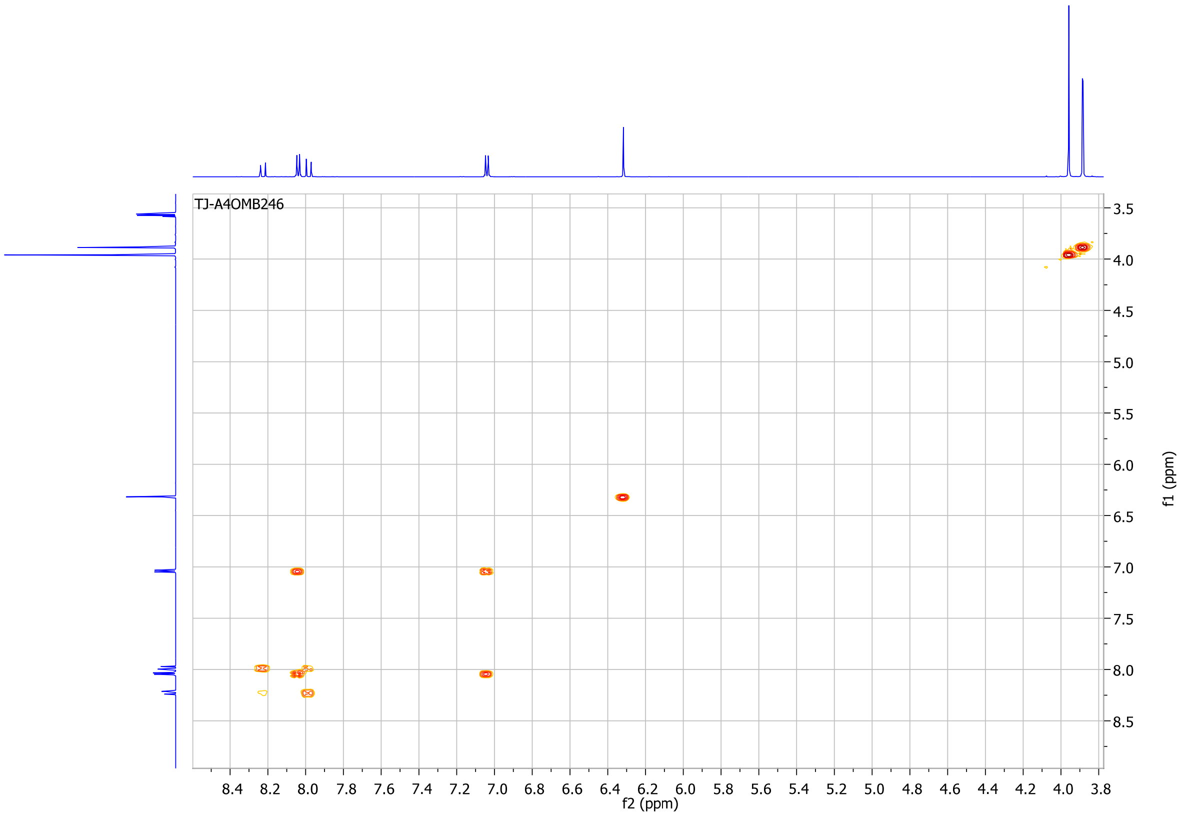

Figure S52 COSY NMR spectrum of 2,4,6,4'-tetramethoxychalcone (**7a**) (600 MHz; acetone-d$_6$).

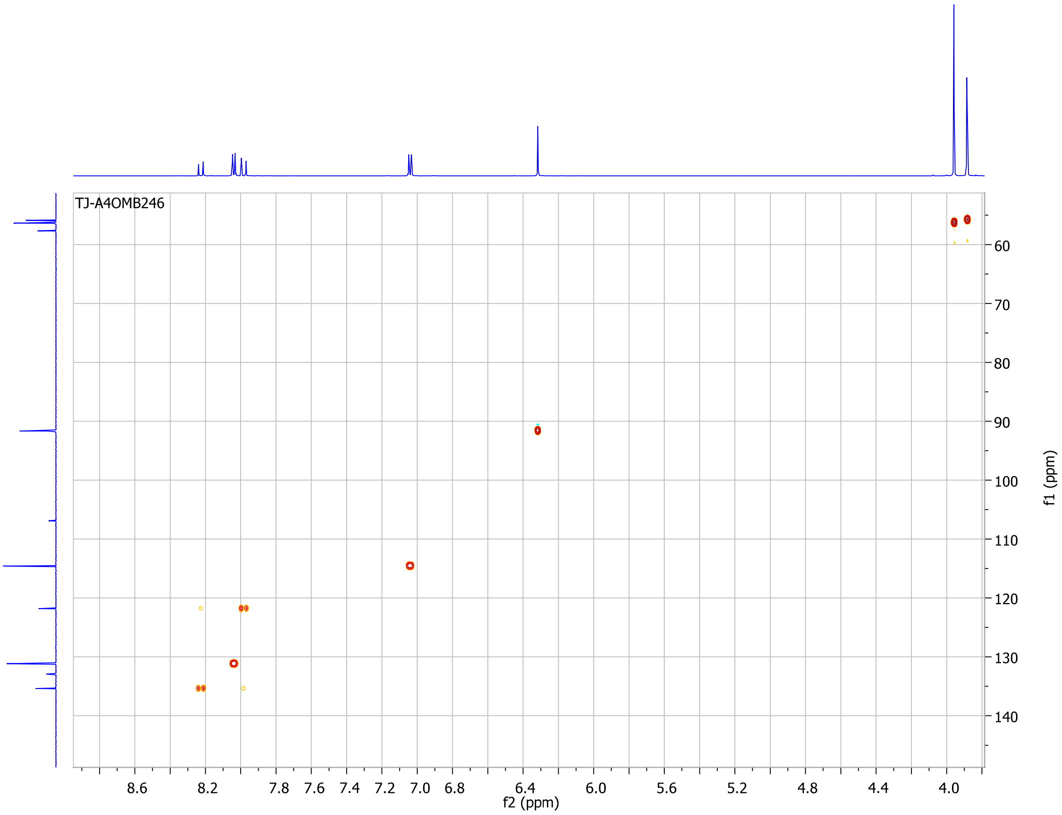

Figure S53 HSQC NMR spectrum of 2,4,6,4'-tetramethoxychalcone (**7a**) (600 MHz; acetone-d$_6$).

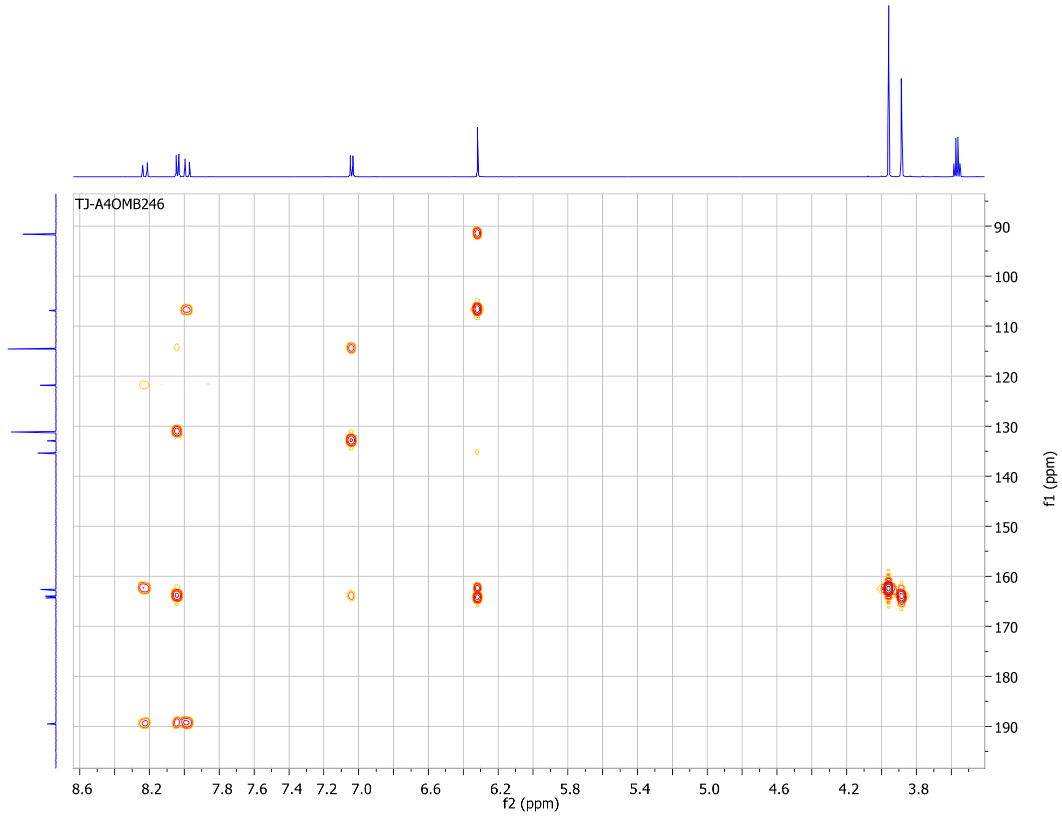

Figure S54 HMBC NMR spectrum of 2,4,6,4'-tetramethoxychalcone (**7a**) (600 MHz; acetone-d$_6$).

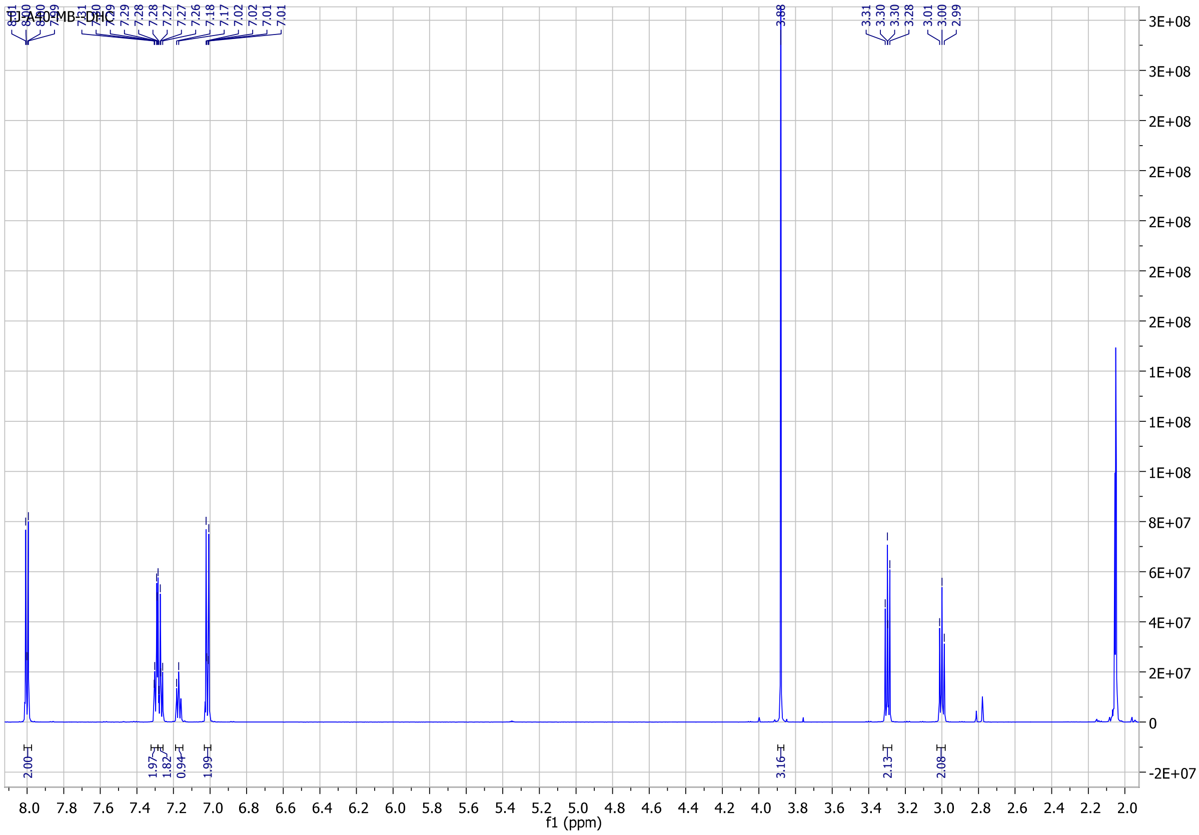

Figure S55 ^{1}H NMR spectrum of 4'-methoxydihydrochalcone (**1c**) (600 MHz; acetone-d_6).

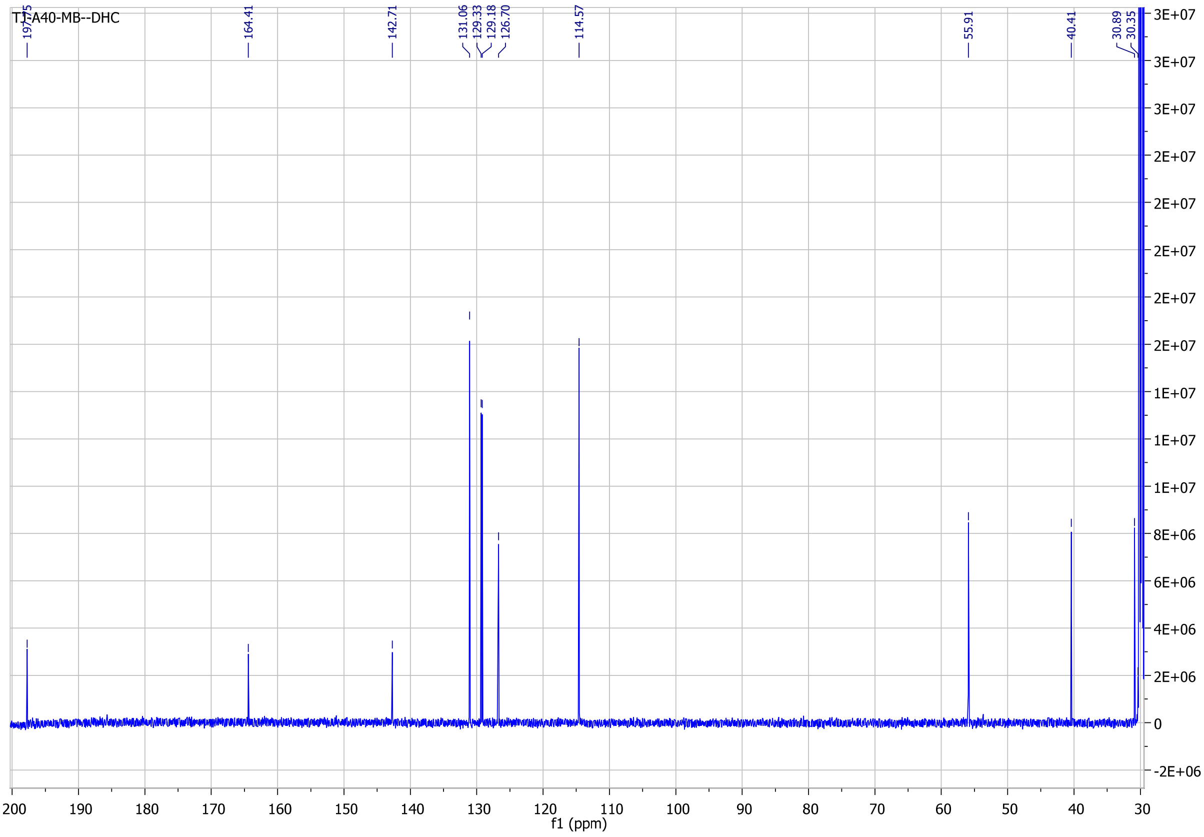

Figure S56 ^{13}C NMR spectrum of 4'-methoxydihydrochalcone (**1c**) (600 MHz; acetone-d_6).

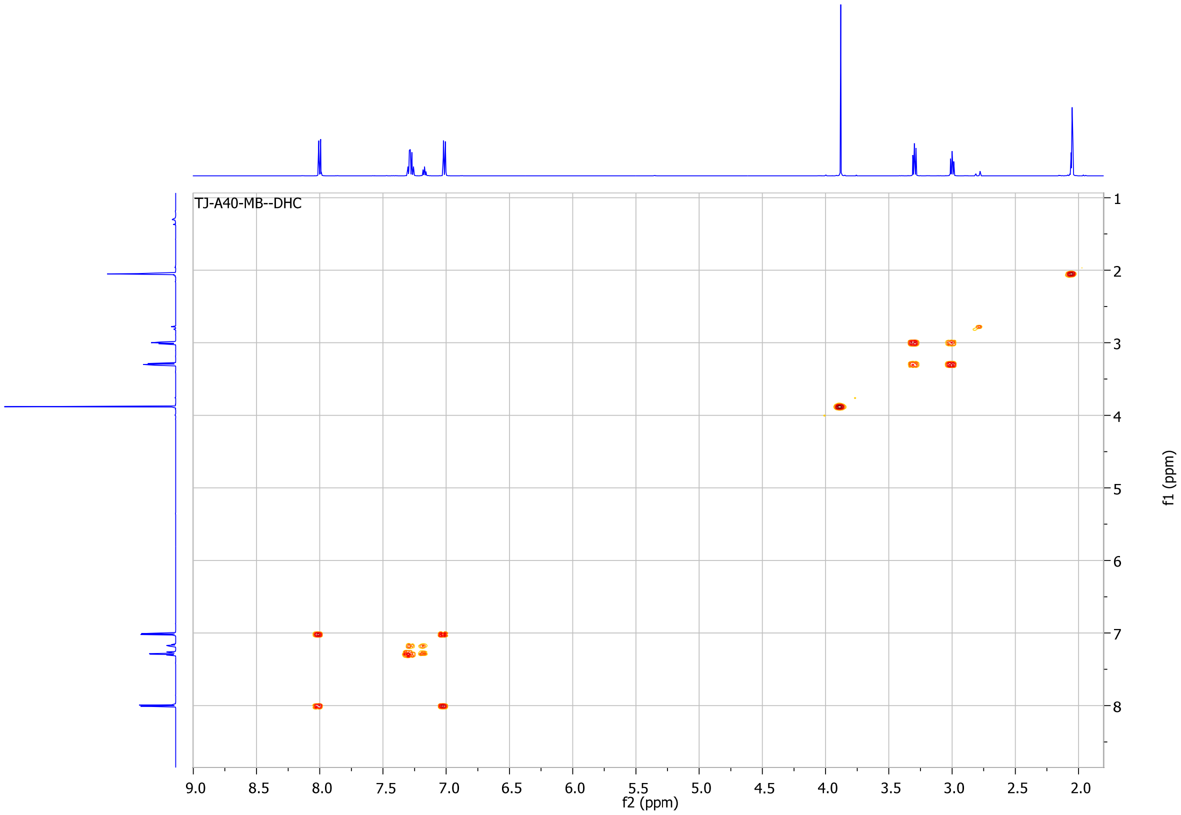

Figure S57 COSY NMR spectrum of 4'-methoxydihydrochalcone (**1c**) (600 MHz; acetone-d6).

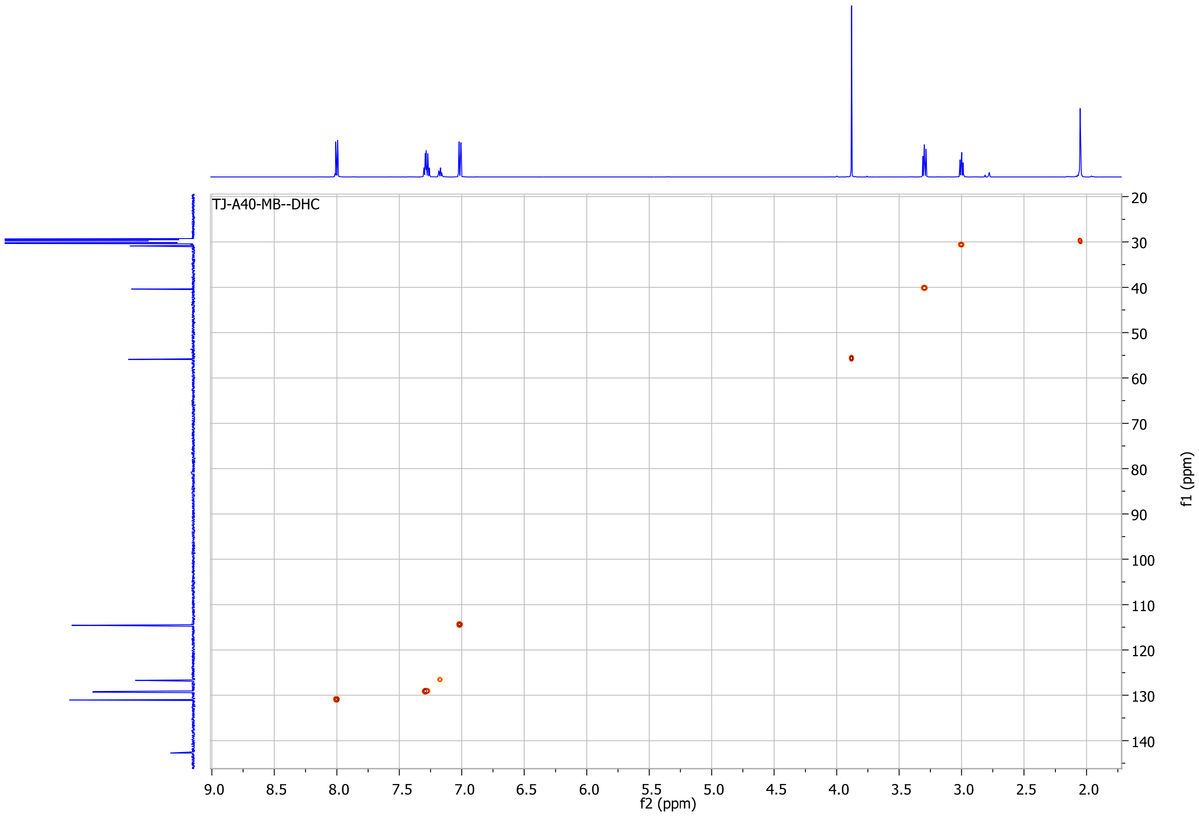

Figure S58 HSQC NMR spectrum of 4'-methoxydihydrochalcone (**1c**) (600 MHz; acetone-d_6).

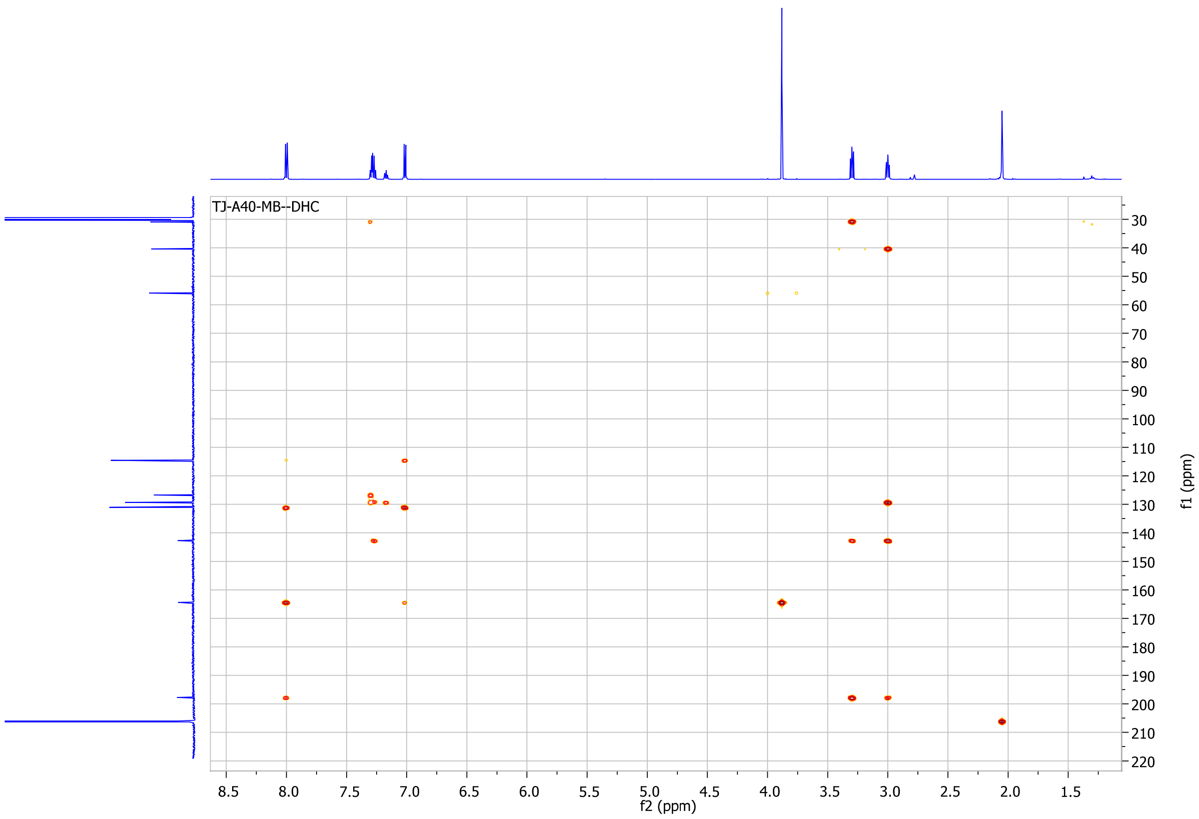

Figure S59 HMBC NMR spectrum of 4'-methoxydihydrochalcone (**1c**) (600 MHz; acetone-d₆).

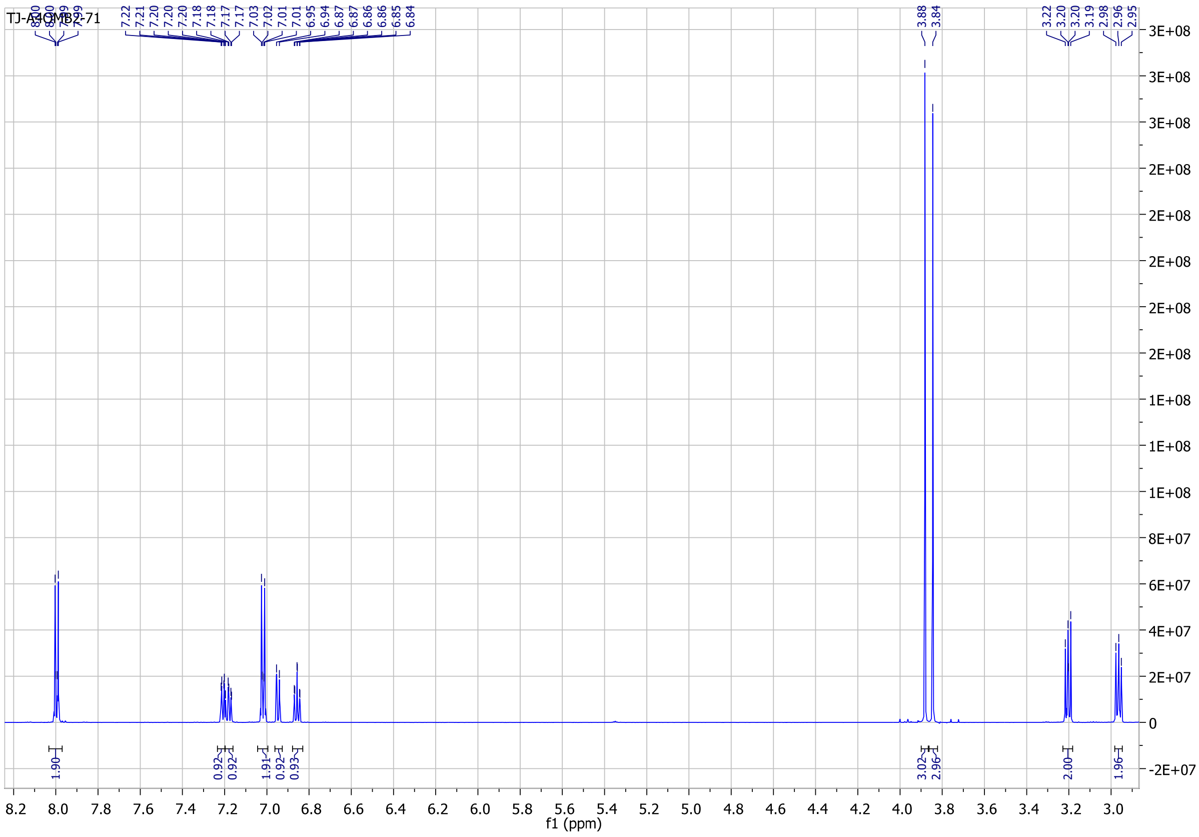

Figure S60 ^{1}H NMR spectrum of 2,4'-dimethoxydihydrochalcone (**2c**) (600 MHz; acetone-d₆).

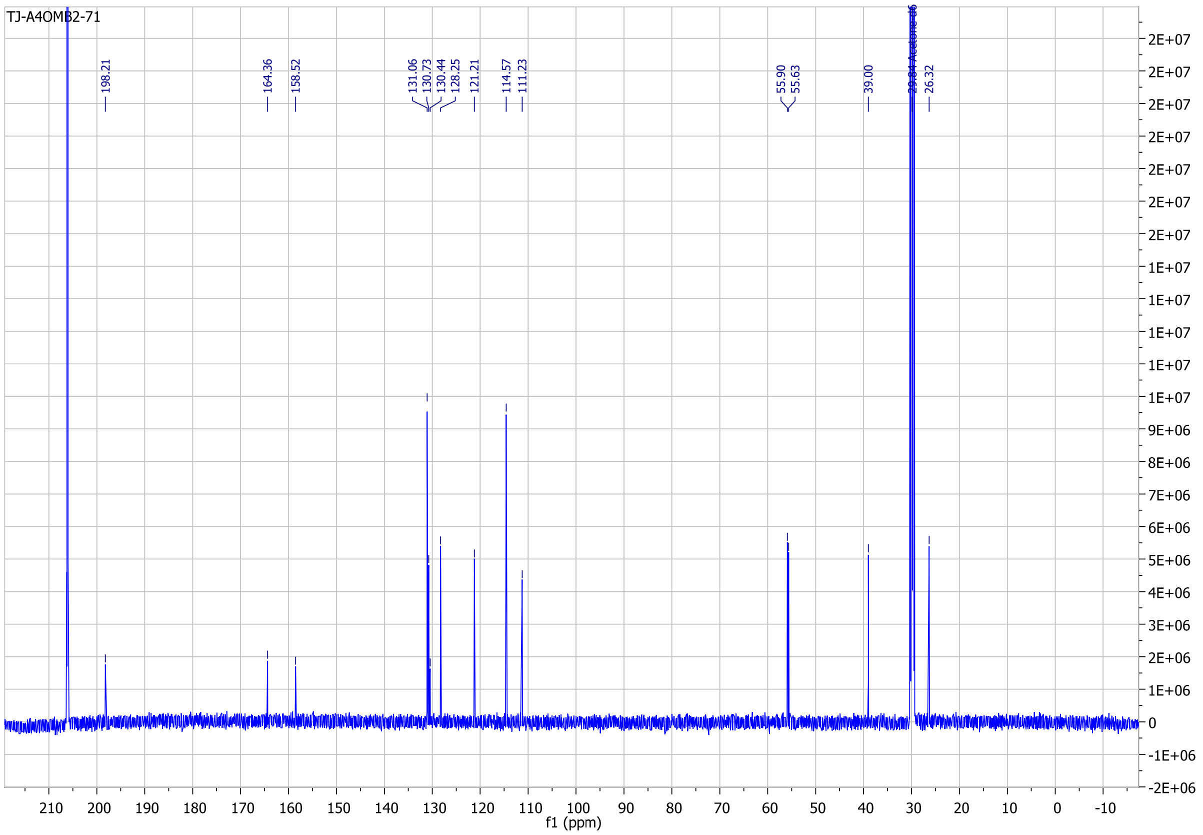

Figure S61 ^{13}C NMR spectrum of 2,4'-dimethoxydihydrochalcone (**2c**) (600 MHz; acetone-d$_6$).

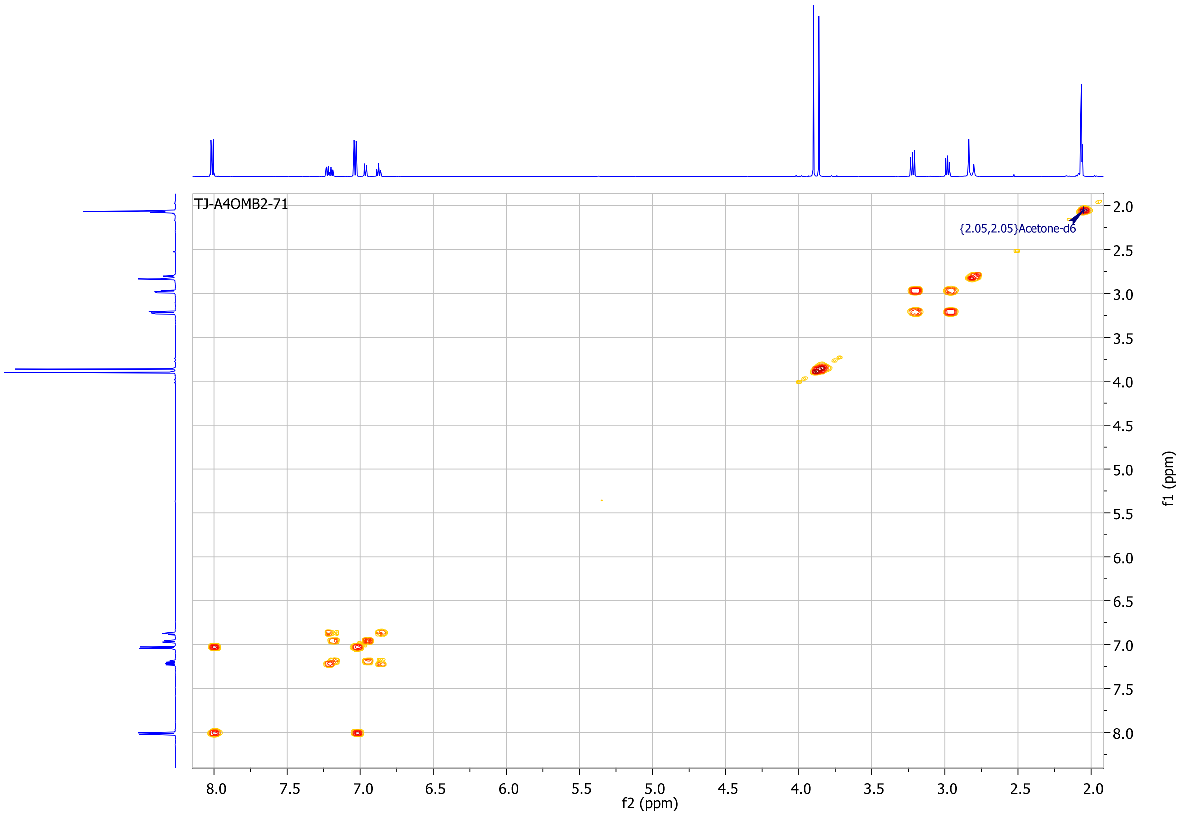

Figure S62 COSY NMR spectrum of 2,4'-dimethoxydihydrochalcone (**2c**) (600 MHz; acetone-d$_6$).

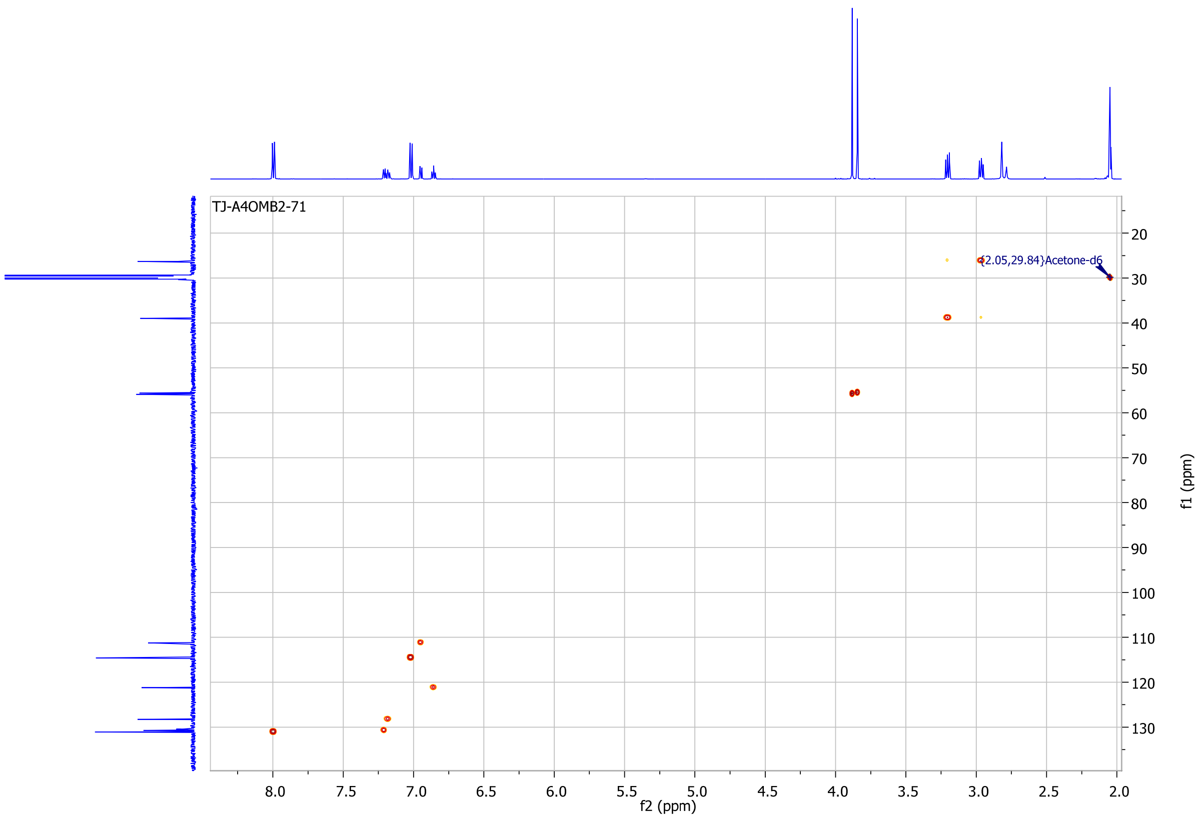

Figure S63 HSQC NMR spectrum of 2,4'-dimethoxydihydrochalcone (**2c**) (600 MHz; acetone-d_6).

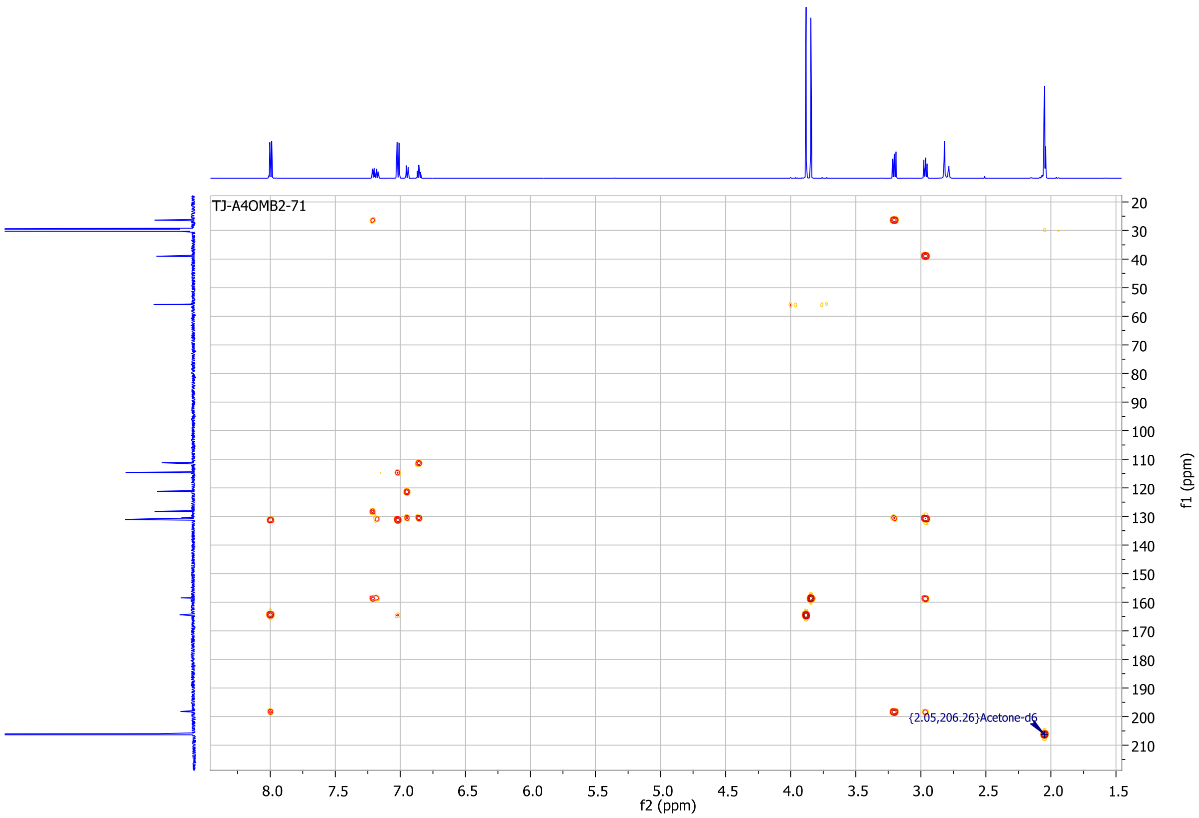

Figure S64 HMBC NMR spectrum of 2,4'-dimethoxydihydrochalcone (**2c**) (600 MHz; acetone-d_6).

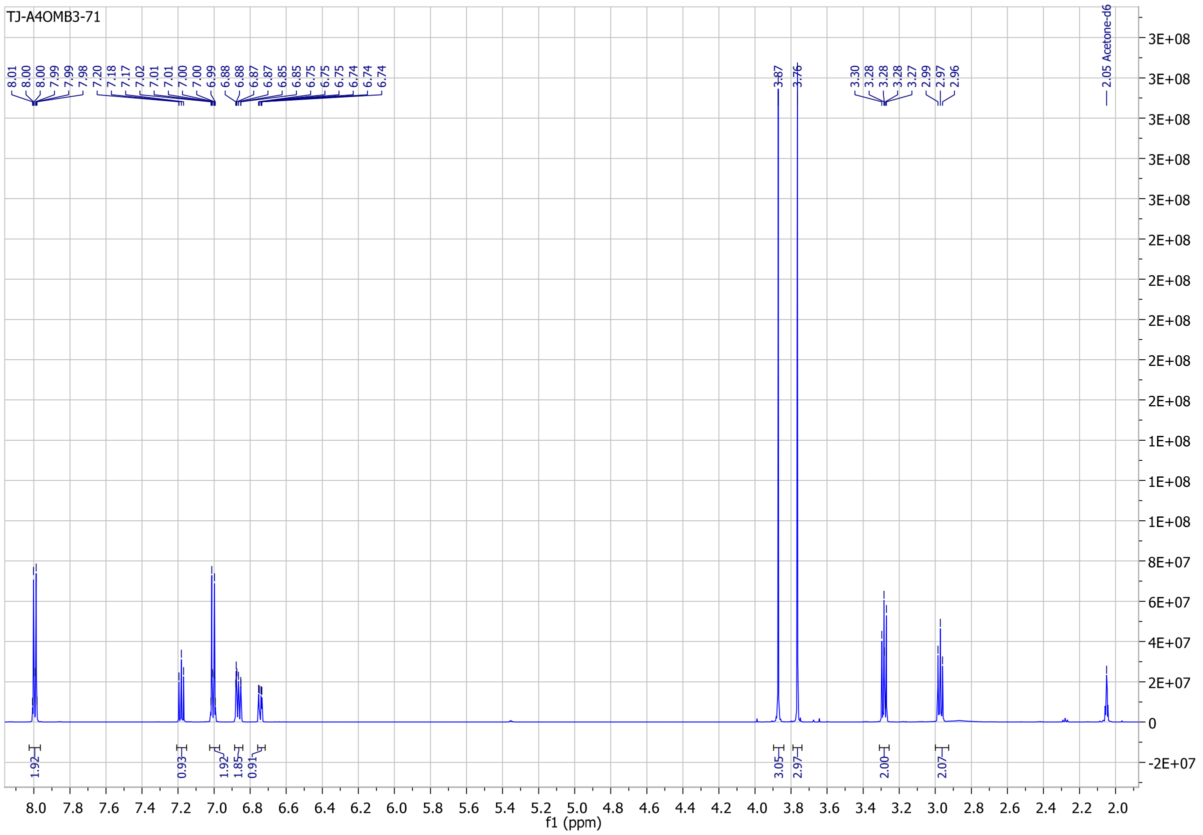

Figure S65 ^{1}H NMR spectrum of 3,4'-dimethoxydihydrochalcone (**3c**) (600 MHz; acetone-d_6).

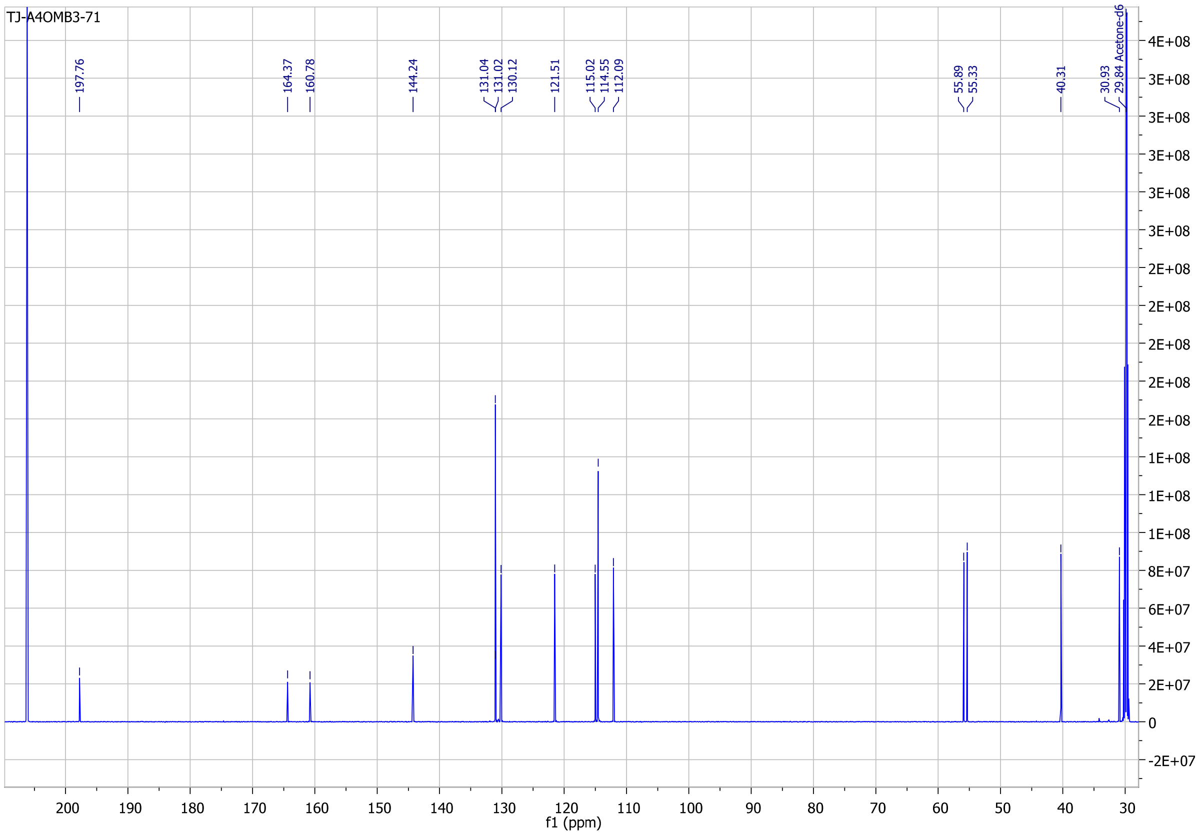

Figure S66 ^{13}C NMR spectrum of 3,4'-dimethoxydihydrochalcone (**3c**) (600 MHz; acetone-d_6).

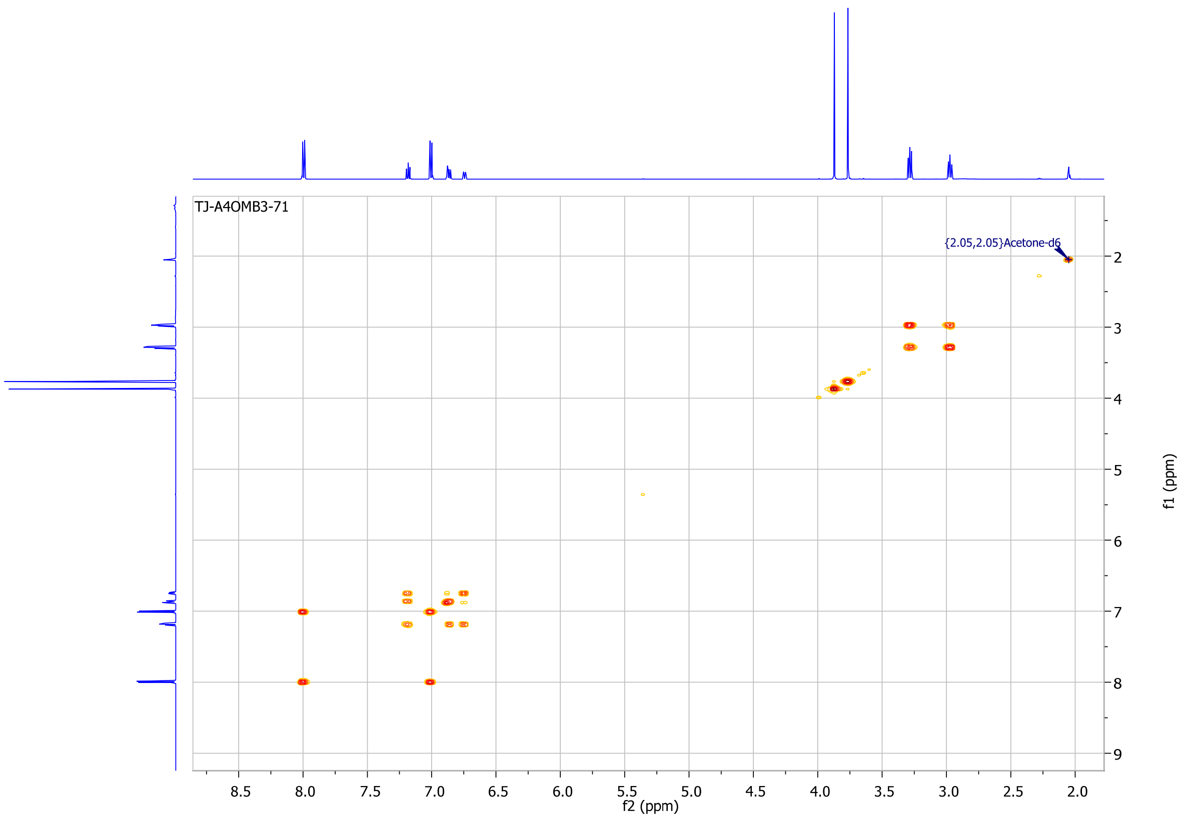

Figure S67 COSY NMR spectrum of 3,4'-dimethoxydihydrochalcone (**3c**) (600 MHz; acetone-d_6).

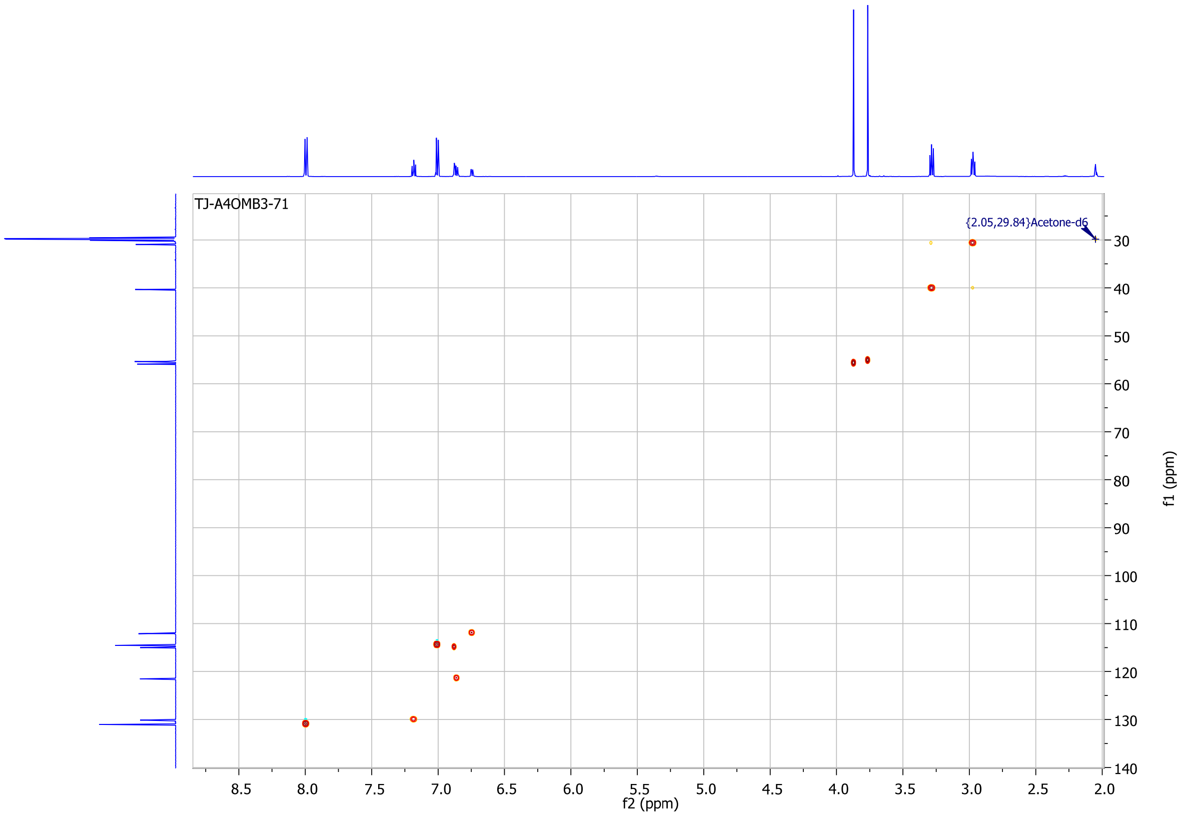

Figure S68 HSQC NMR spectrum of 3,4'-dimethoxydihydrochalcone (**3c**) (600 MHz; acetone-d_6).

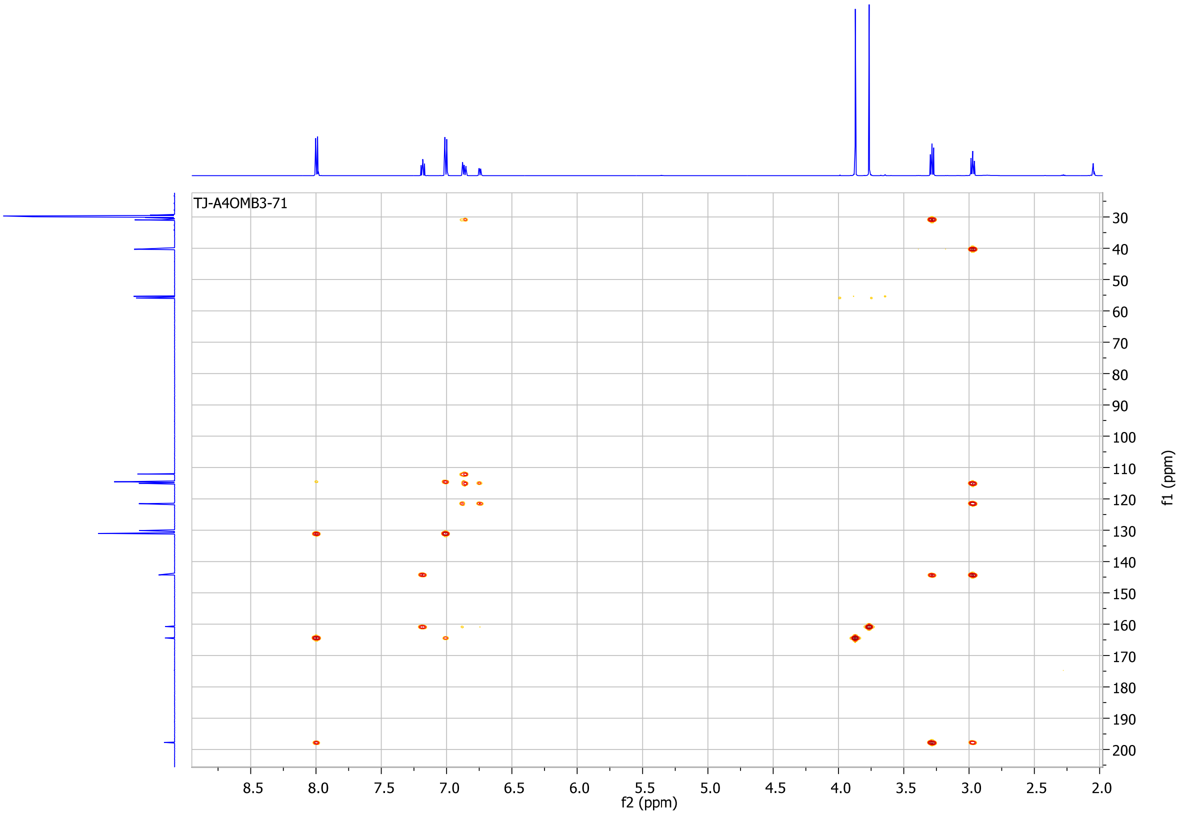

Figure S69 HMBC NMR spectrum of 3,4'-dimethoxydihydrochalcone (**3c**) (600 MHz; acetone-d6).

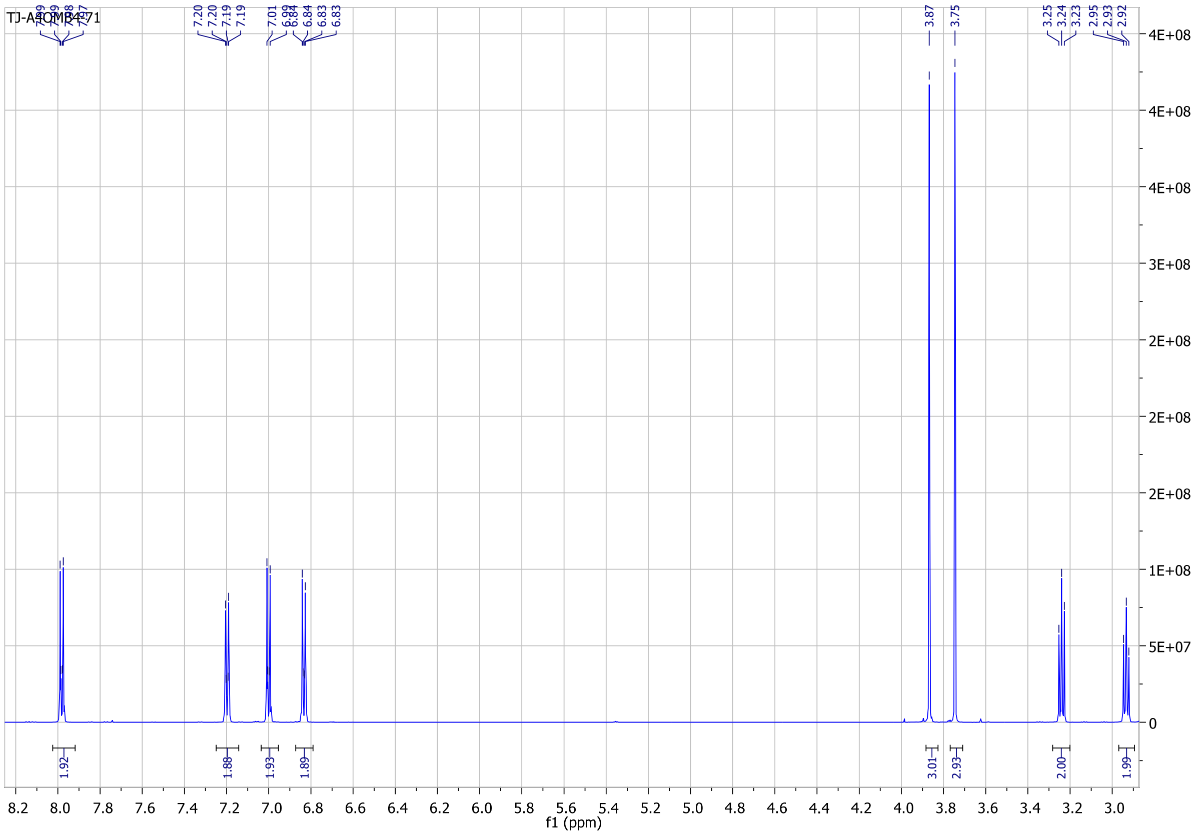

Figure S70 ¹H NMR spectrum of 4,4'-dimethoxydihydrochalcone (**4c**) (600 MHz; acetone-d6).

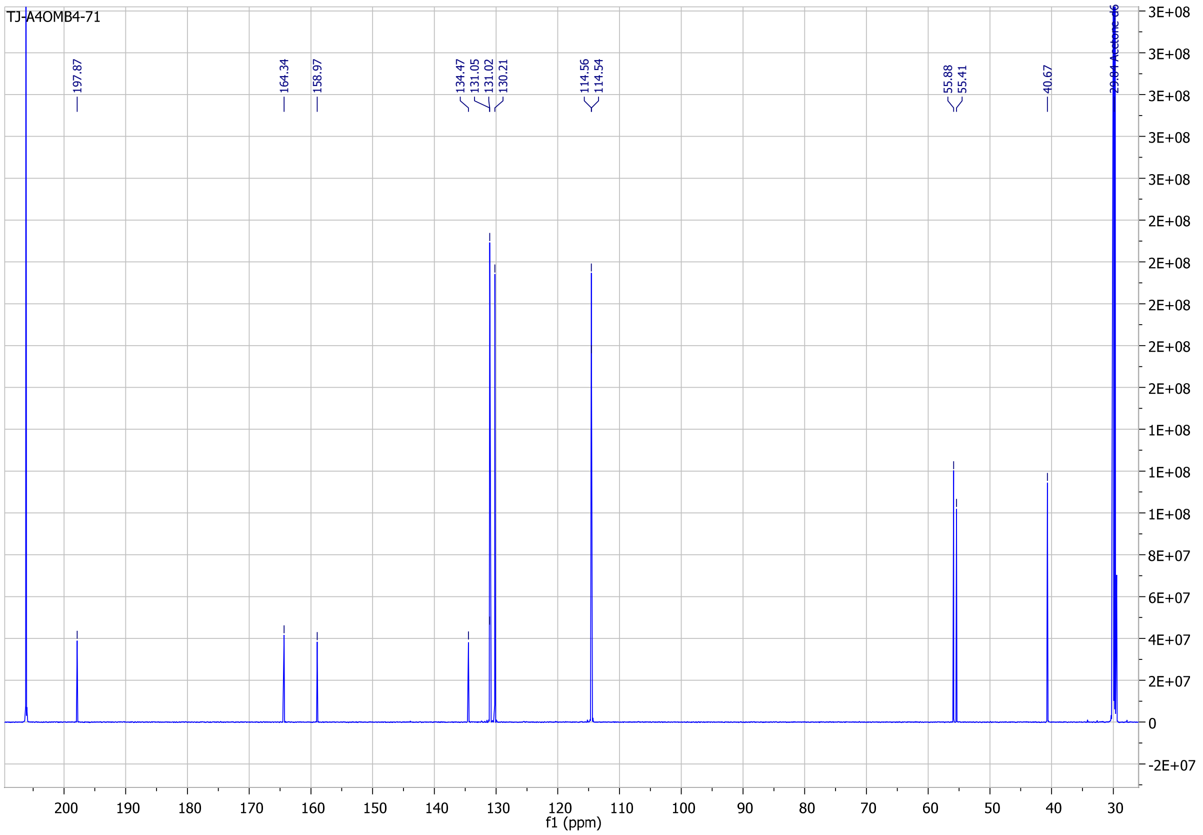

Figure S71 ^{13}C NMR spectrum of 4,4'-dimethoxydihydrochalcone (**4c**) (600 MHz; acetone-d$_6$).

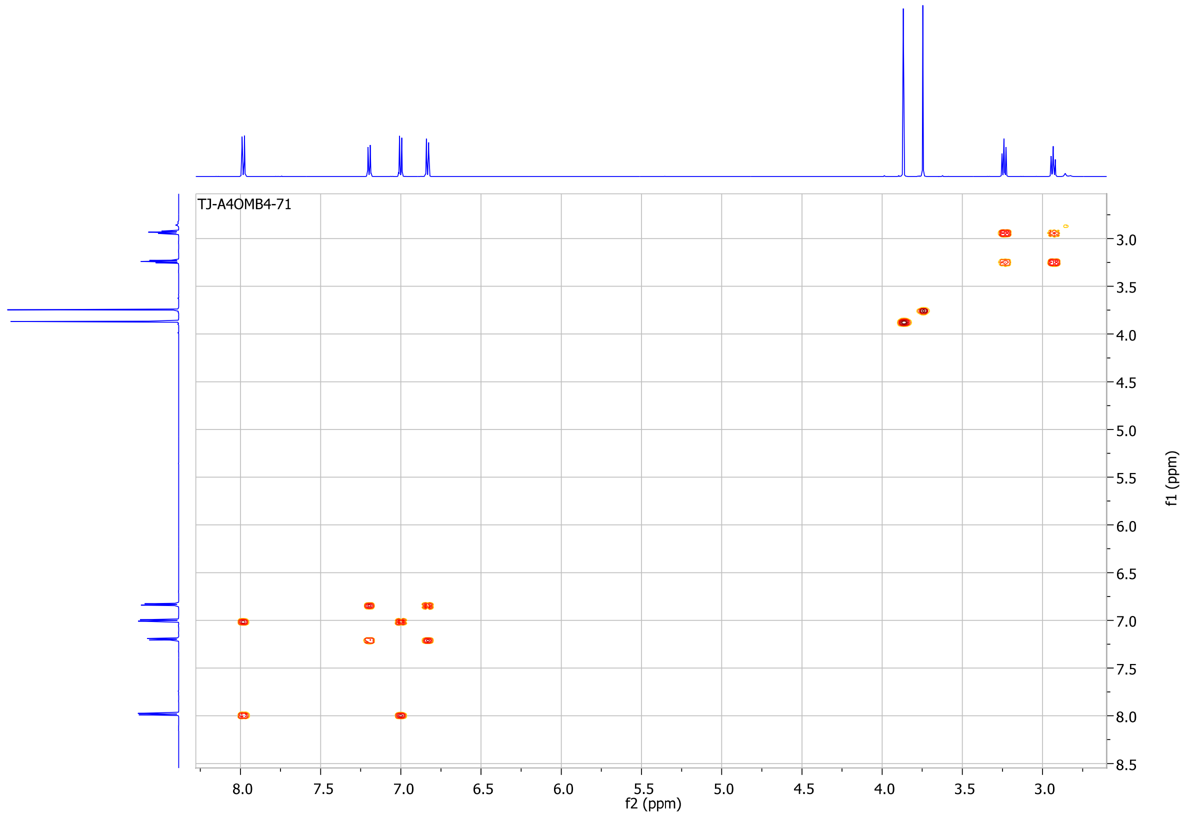

Figure S72 COSY NMR spectrum of 4,4'-dimethoxydihydrochalcone (**4c**) (600 MHz; acetone-d$_6$).

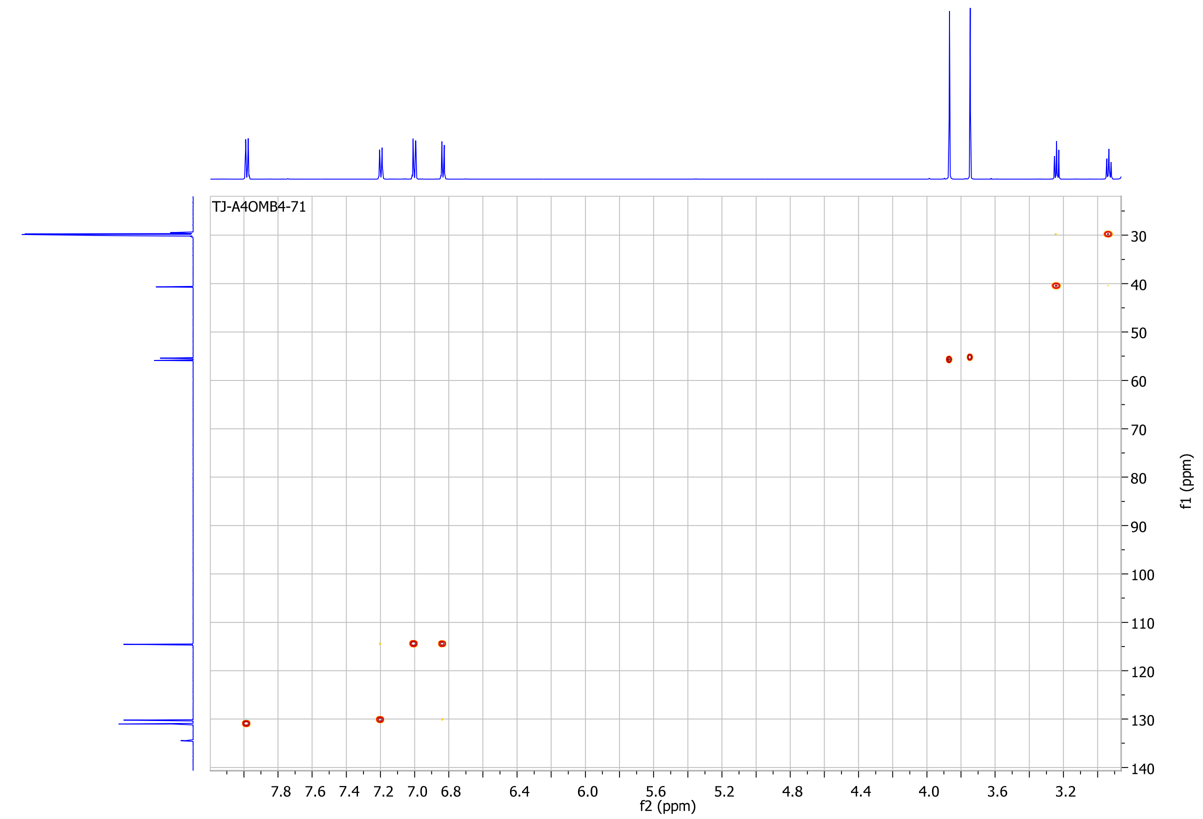

Figure S73 HSQC NMR spectrum of 4,4'-dimethoxydihydrochalcone (**4c**) (600 MHz; acetone-d6).

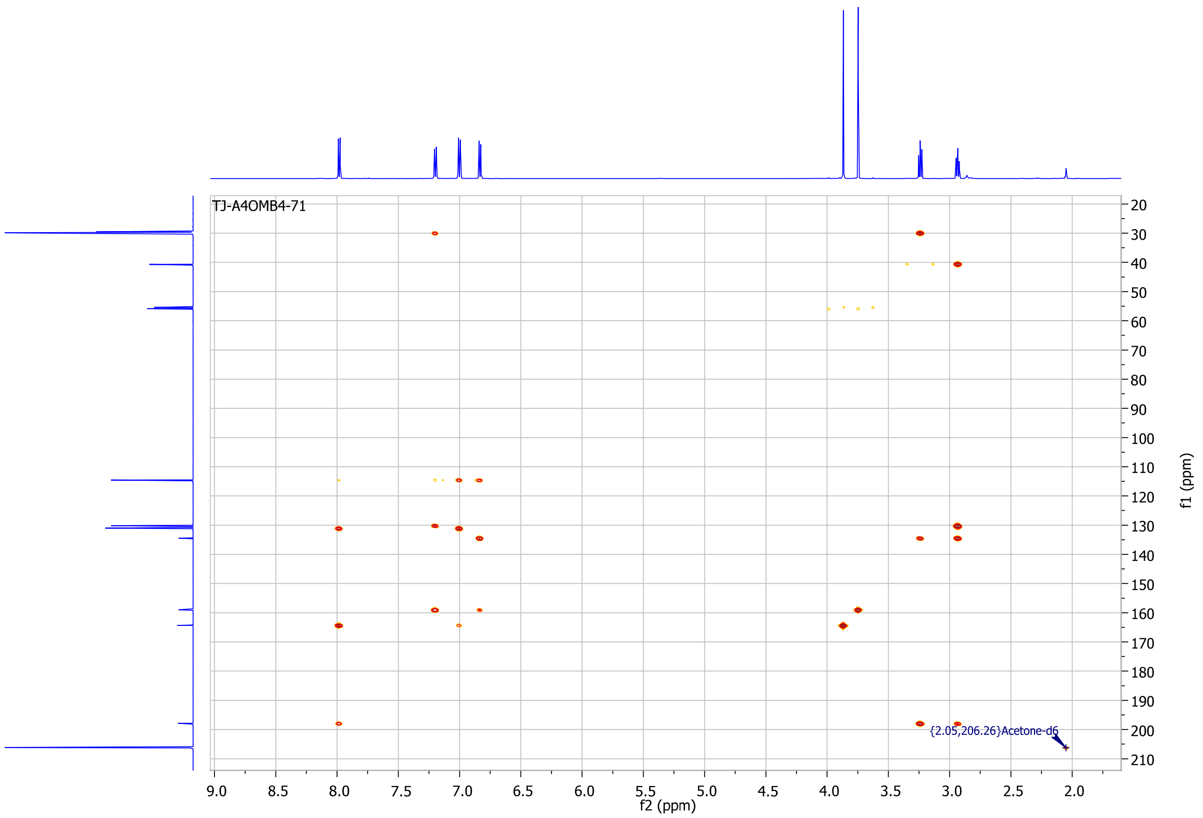

Figure S74 HMBC NMR spectrum of 4,4'-dimethoxydihydrochalcone (**4c**) (600 MHz; acetone-d6).

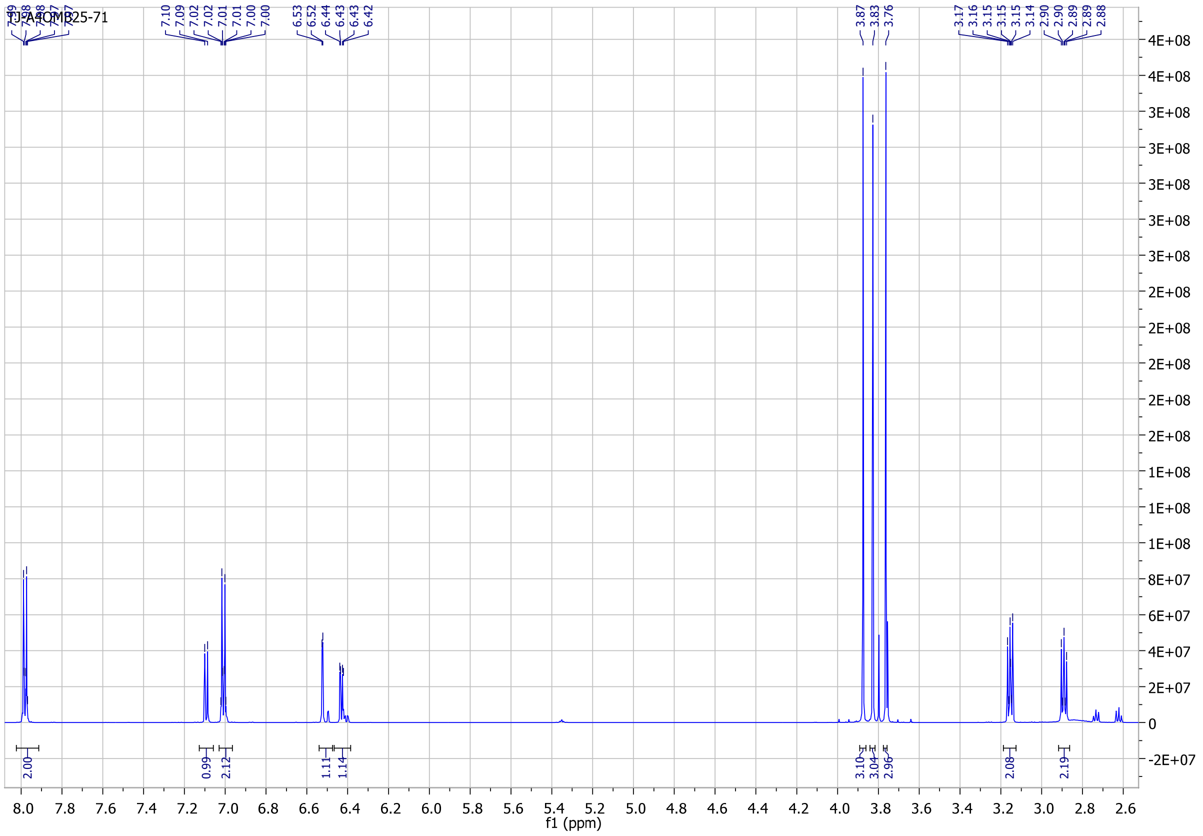

Figure S75 ¹H NMR spectrum of 2,5,4'-trimethoxydihydrochalcone (**5c**) (600 MHz; acetone-d_6).

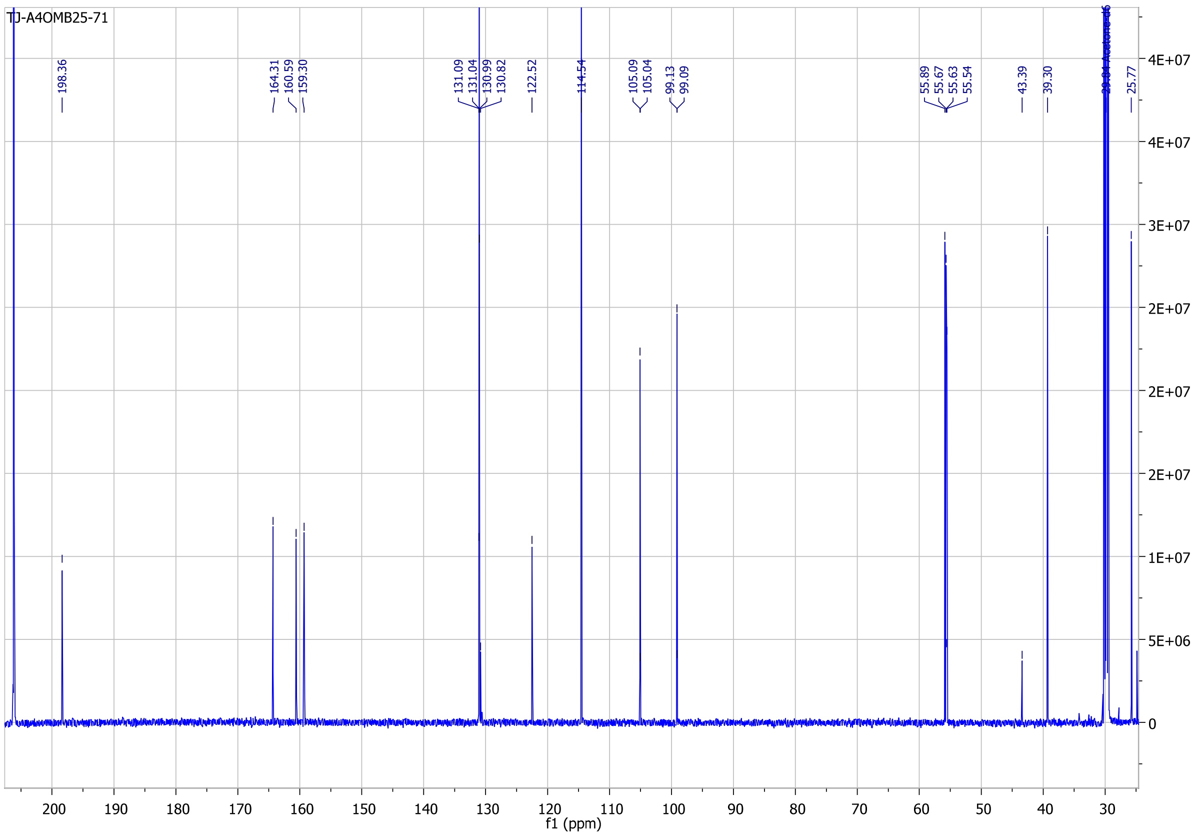

Figure S76 ¹³C NMR spectrum of 2,5,4'-trimethoxydihydrochalcone (**5c**) (600 MHz; acetone-d_6).

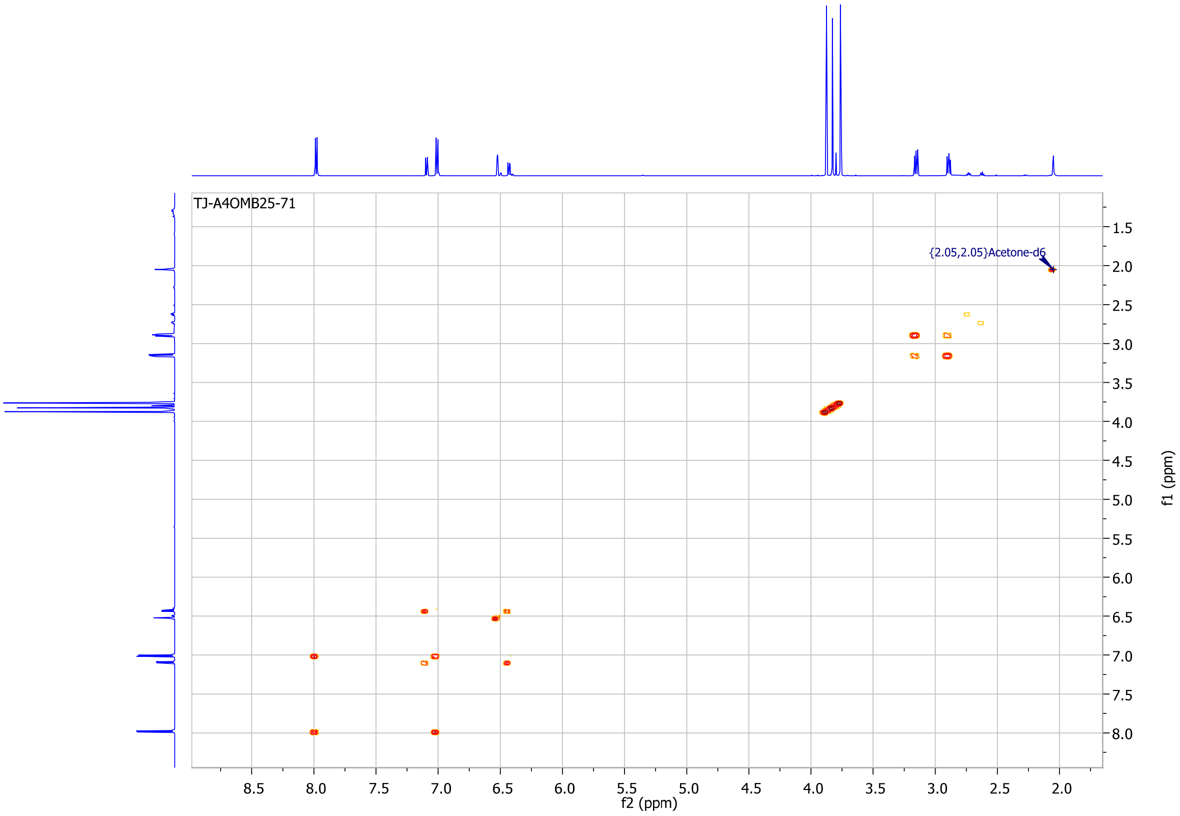

Figure S77 COSY NMR spectrum of 2,5,4'-trimethoxydihydrochalcone (**5c**) (600 MHz; acetone-d_6).

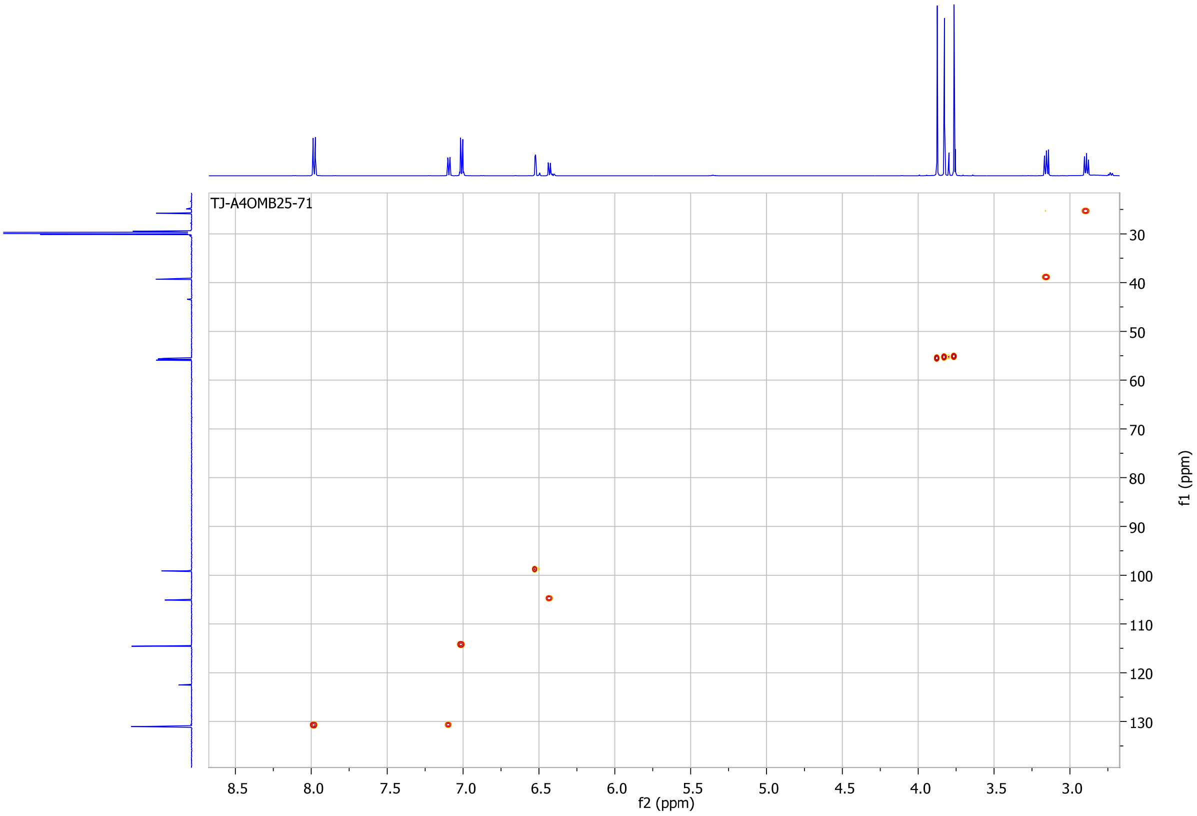

Figure S78 HSQC NMR spectrum of 2,5,4'-trimethoxydihydrochalcone (**5c**) (600 MHz; acetone-d_6).

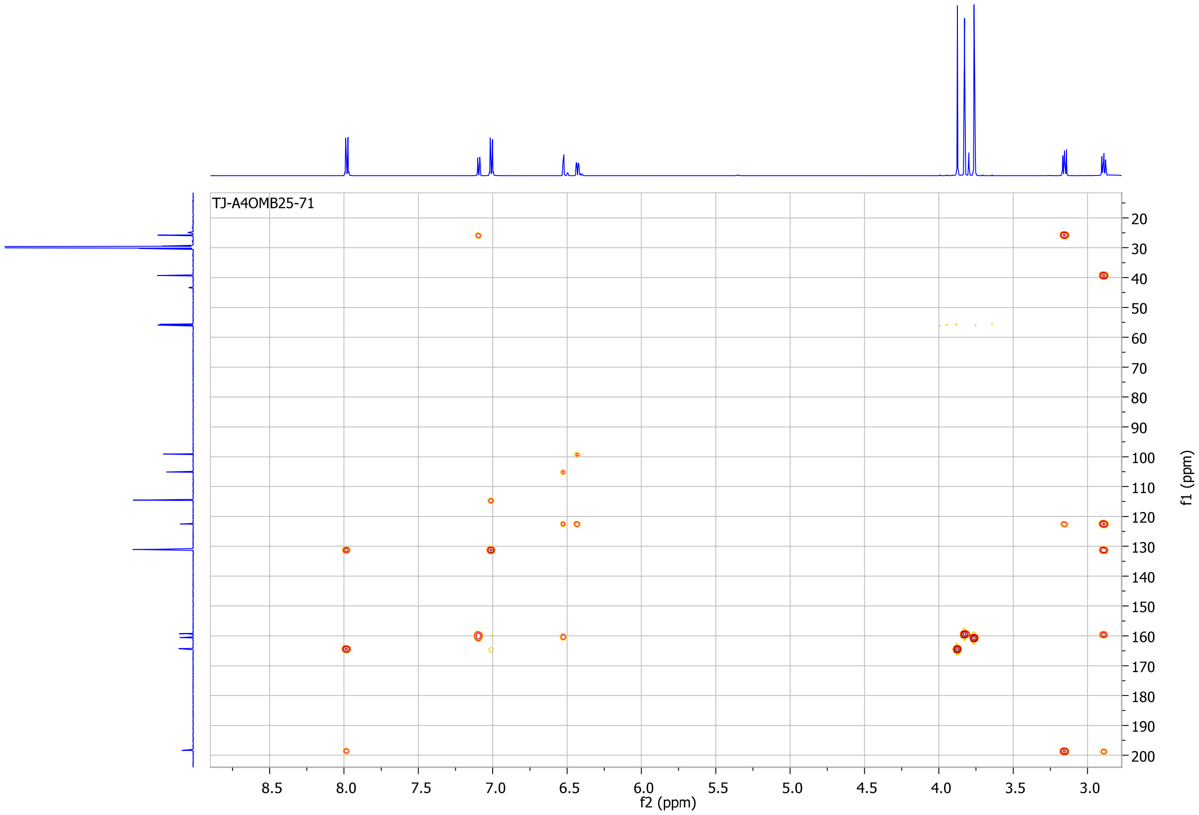

Figure S79 HMBC NMR spectrum of 2,5,4'-trimethoxydihydrochalcone (**5c**) (600 MHz; acetone-d_6).

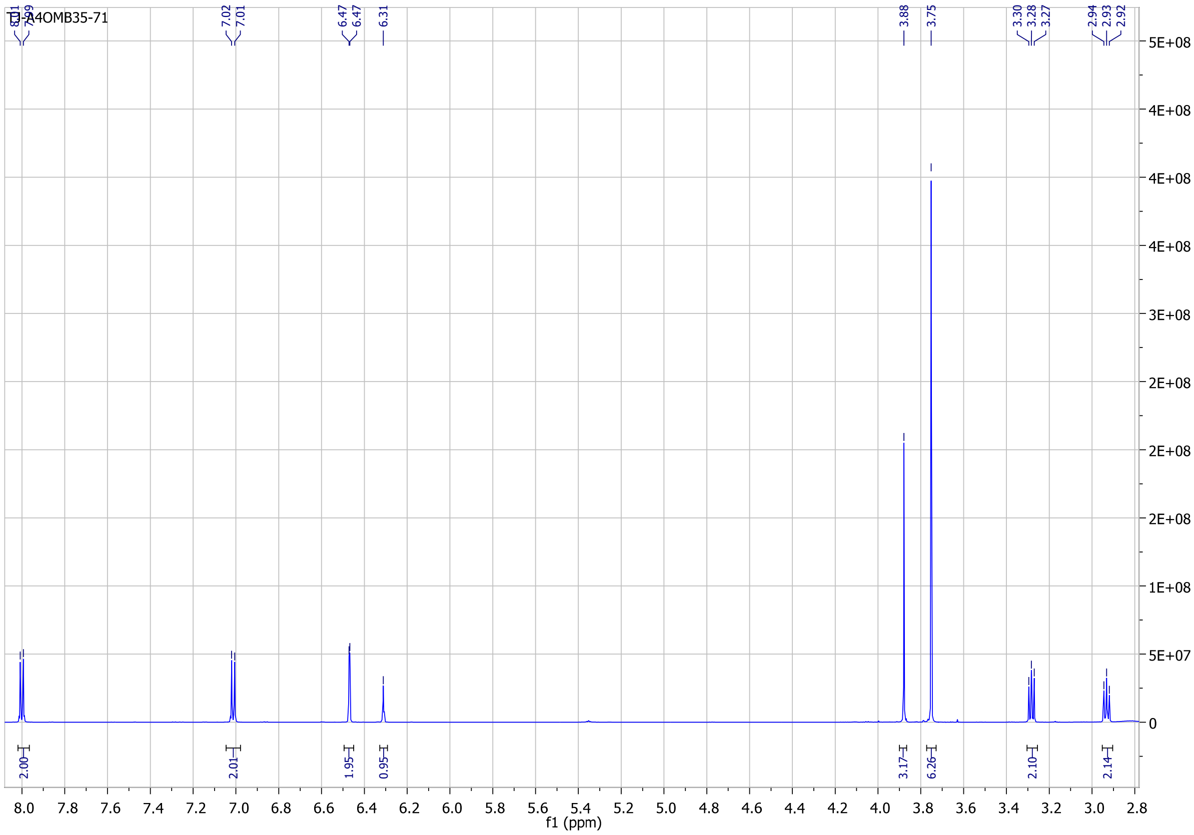

Figure S80 ^{1}H NMR spectrum of 3,5,4'-trimethoxydihydrochalcone (**6c**) (600 MHz; acetone-d_6).

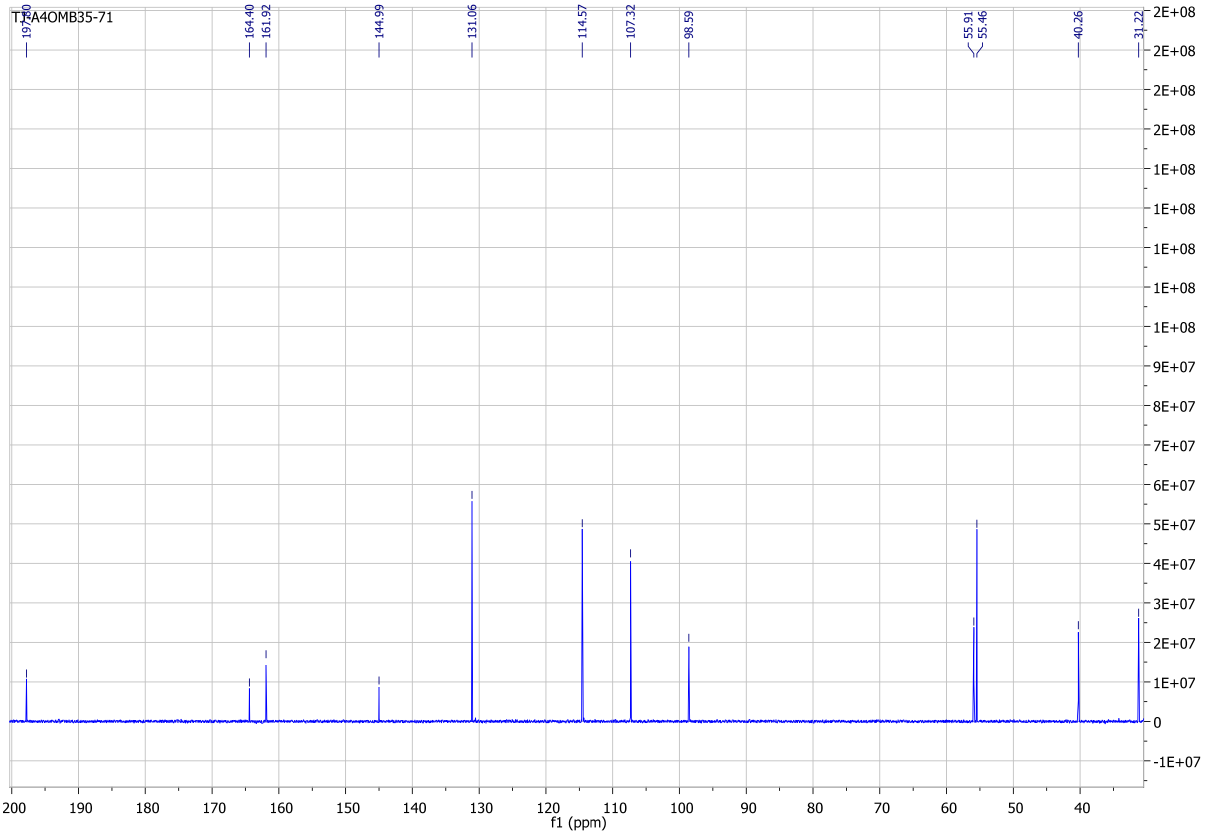

Figure S81 ^{13}C NMR spectrum of 3,5,4'-trimethoxydihydrochalcone (**6c**) (600 MHz; acetone-d_6).

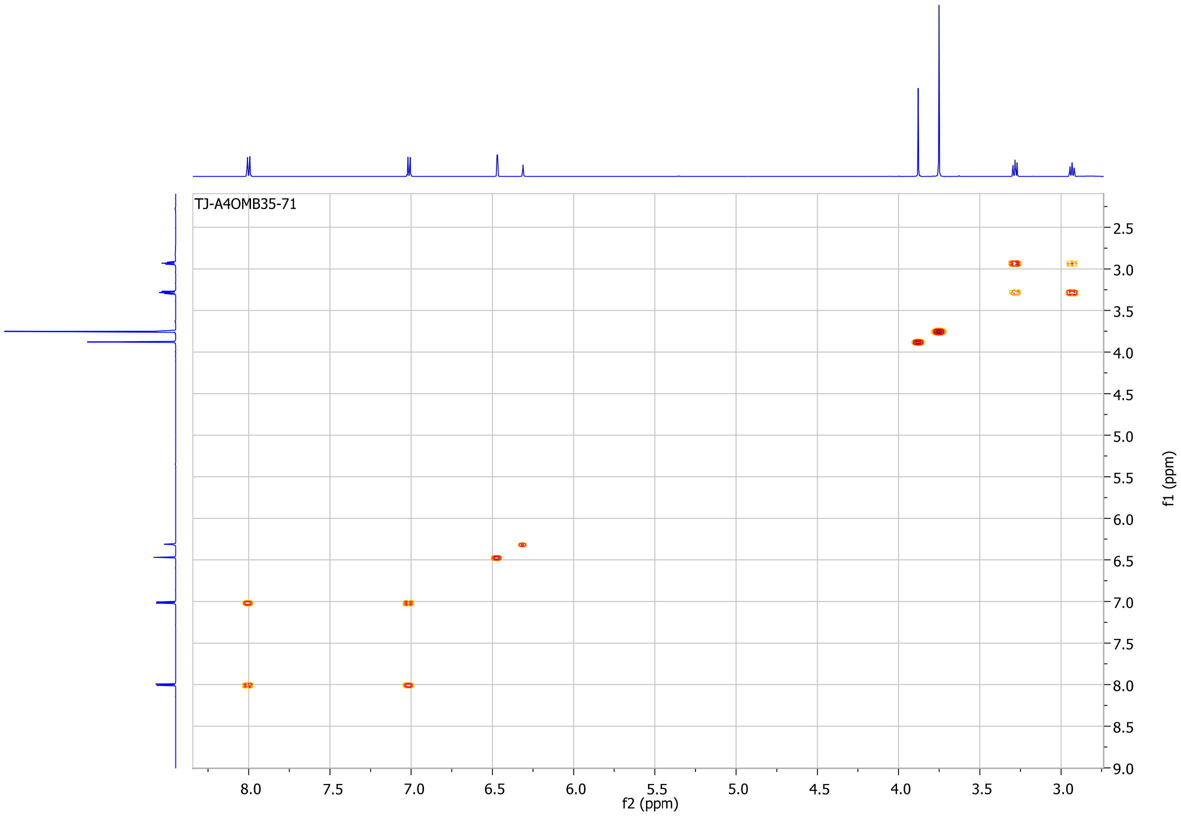

Figure S82 COSY NMR spectrum of 3,5,4'-trimethoxydihydrochalcone (**6c**) (600 MHz; acetone-d_6).

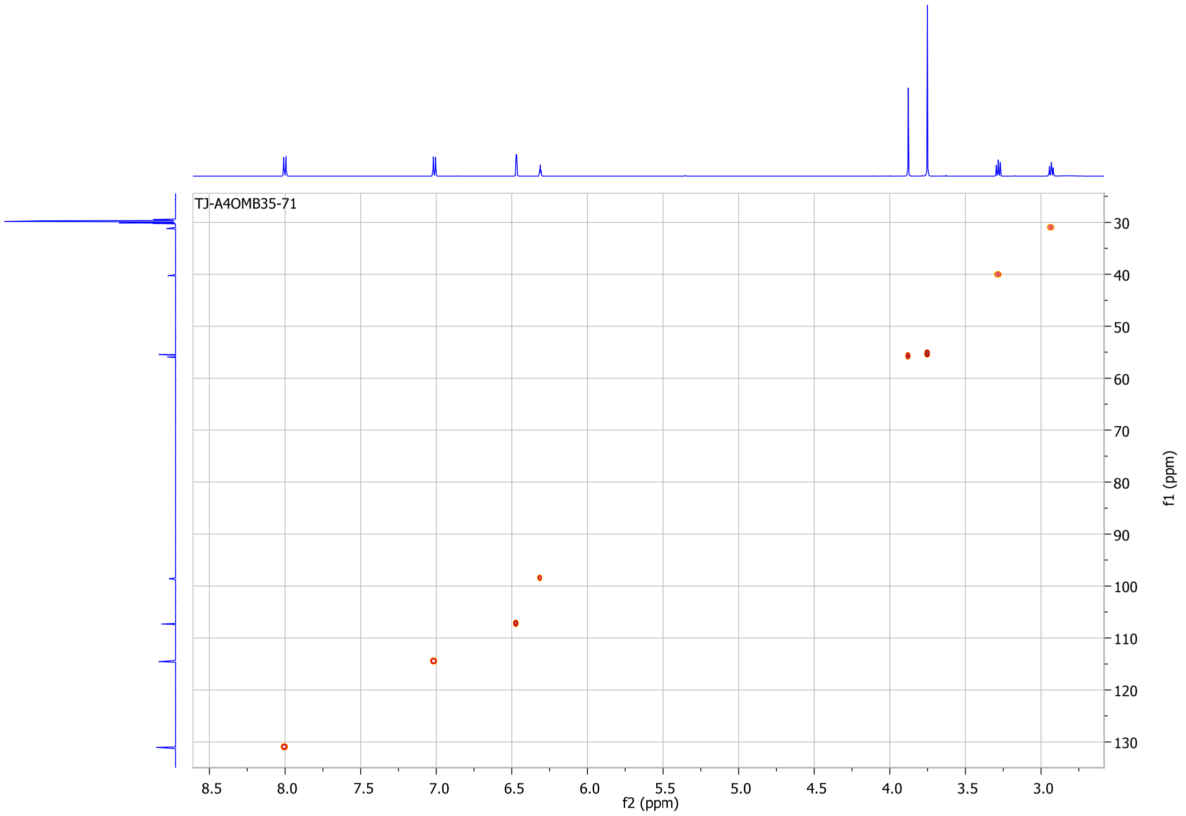

Figure S83 HSQC NMR spectrum of 3,5,4'-trimethoxydihydrochalcone (**6c**) (600 MHz; acetone-d6).

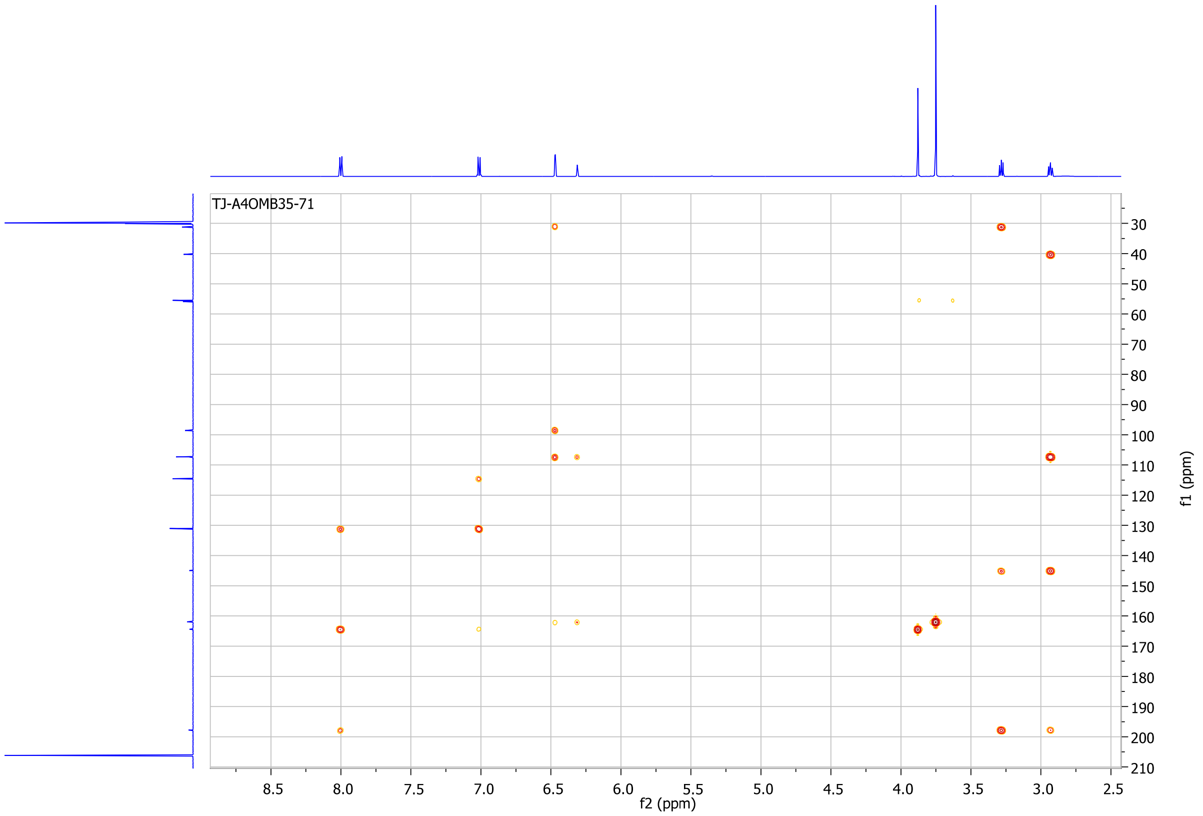

Figure S84 HMBC NMR spectrum of 3,5,4'-trimethoxydihydrochalcone (**6c**) (600 MHz; acetone-d6).

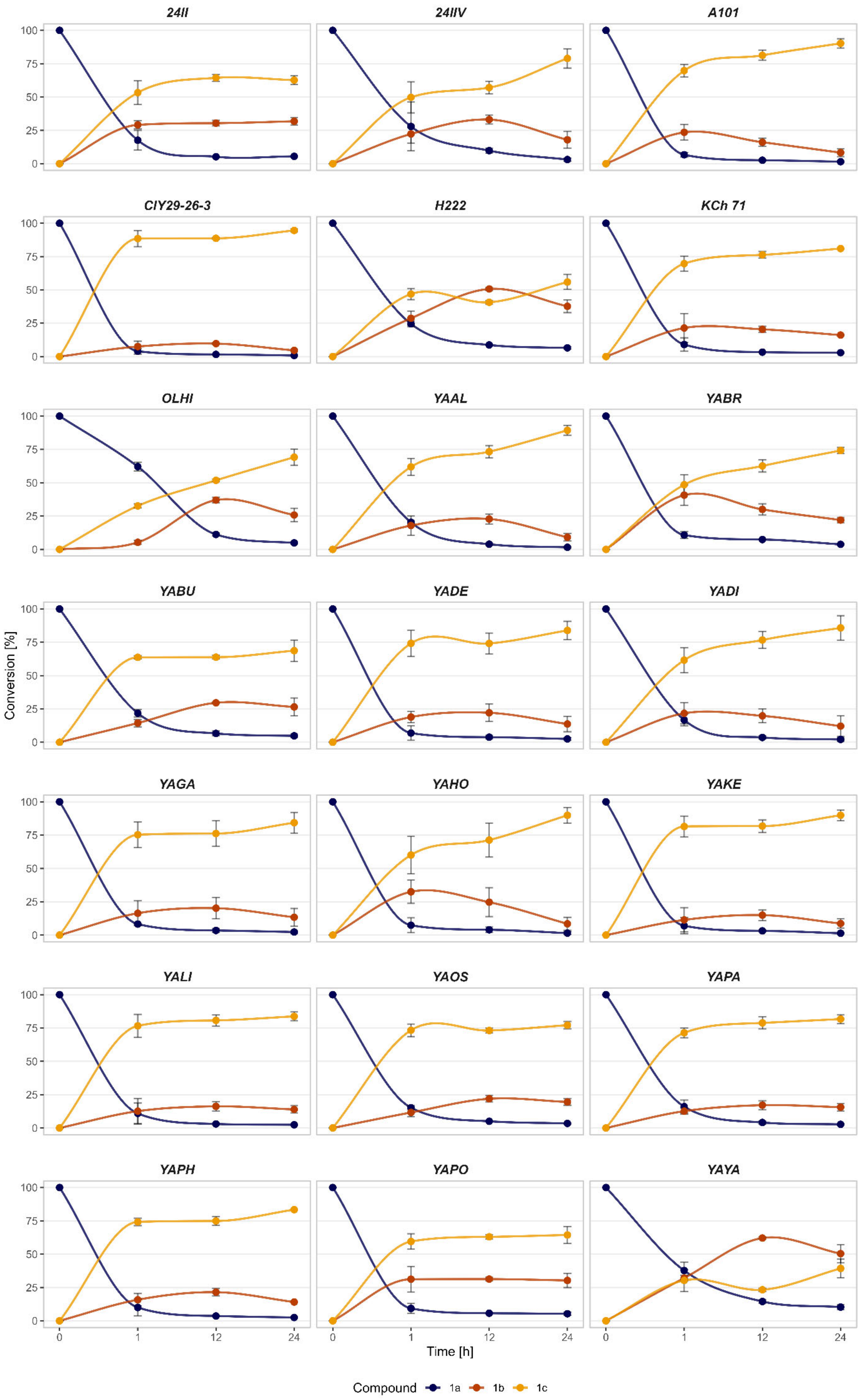

Figure S85 Time-course of the biotransformation of **1a** to **1c**. Compound **1b** represents a photo-induced isomer of **1a**, formed independently of microbial activity.

Table S1 Comparative analysis of identified *Yarrowia* OYE sequences retrieved from the WGS database, showing high structural conservation across the clade.

Sequence ID (Contig/Locus)	Organism / Reference	Identity [%]	Query Coverage [%]	E-value	Bit-score	Status
ULGU01000002.1	*Y. osloensis*	97.59	100	0.0	756	Ortholog
VJVY01000001.1	*Y. divulgata*	96.25	100	0.0	749	Ortholog
ULGS01000005.1	*Y. galli*	96.25	100	0.0	745	Ortholog
UTQH01000001.1	*Y. lipolytica*	96.25	100	0.0	745	Ortholog
ULGY01000004.1	*Y. deformans*	95.98	100	0.0	743	Ortholog
ULGX01000006.1	*Y. bubula*	84.72	100	0.0	660	Ortholog
ULGV01000003.1	*Y. hollandica*	83.91	100	0.0	654	Ortholog
ULGN01000003.1	*Y. alimentaria*	82.04	100	0.0	639	Ortholog
ULGX01000002.1	*Y. bubula* (H2)	57.61	98	9.67e-127	406	Paralog
